# Computations Underlying Social Hierarchy Learning: Distinct Neural Mechanisms for Updating and Representing Self-Relevant Information

**DOI:** 10.1016/j.neuron.2016.10.052

**Published:** 2016-12-07

**Authors:** Dharshan Kumaran, Andrea Banino, Charles Blundell, Demis Hassabis, Peter Dayan

**Affiliations:** 1 Google DeepMind, 5 New Street Square, London EC4A 3TW, UK; 2 Gatsby Computational Neuroscience Unit, 25 Howland Street, London W1T 4JG, UK; 3 Institute of Cognitive Neuroscience, University College London, 17 Queen Square, London WC1N 3AR, UK

**Keywords:** social hierarchy, memory, learning, prefrontal cortex, hippocampus, Bayesian, reinforcement learning

## Abstract

Knowledge about social hierarchies organizes human behavior, yet we understand little about the underlying computations. Here we show that a Bayesian inference scheme, which tracks the power of individuals, better captures behavioral and neural data compared with a reinforcement learning model inspired by rating systems used in games such as chess. We provide evidence that the medial prefrontal cortex (MPFC) selectively mediates the updating of knowledge about one’s own hierarchy, as opposed to that of another individual, a process that underpinned successful performance and involved functional interactions with the amygdala and hippocampus. In contrast, we observed domain-general coding of rank in the amygdala and hippocampus, even when the task did not require it. Our findings reveal the computations underlying a core aspect of social cognition and provide new evidence that self-relevant information may indeed be afforded a unique representational status in the brain.

## Highlights

• Social hierarchy learning accounted for by a Bayesian inference scheme
• Amygdala and hippocampus support domain-general social hierarchy learning
• Medial prefrontal cortex selectively updates knowledge about one’s own hierarchy
• Rank signals generated by these neural structures in absence of task demands

## Teaser

How do we learn the power structure within our social community? Kumaran et al. show that this involves the maintenance and updating of beliefs about individuals’ power, with the medial prefrontal cortex specifically supporting knowledge about one’s own social hierarchy.

## Introduction

Considerable evidence suggests that groups of humans, non-human primates, and a variety of other species are arranged in linear social dominance hierarchies that are stable over relatively long periods of time. Knowing these relative social ranks is critical for selecting advantageous allies and avoiding potentially dangerous conflicts (Cheney and Seyfarth, 1990, Rushworth et al., 2013). At least two sources of information may be used to guide judgments of social rank. One is the physical appearance of an individual (e.g., facial features and body posture) (Marsh et al., 2004, Todorov et al., 2008, Zink et al., 2008). Such perceptual cues are thought to be widely used in the animal kingdom to indicate rank (e.g., plumage) (Byrne and Bates, 2010) and may provide a relatively coarse heuristic with which to rapidly assess the threat posed by an unfamiliar individual in primates (e.g., an intruder) (Marsh et al., 2004, Todorov et al., 2008, Whalen, 1998). In contrast, the other source of information, namely, experience of previous encounters between (pairs of) individuals, is more robust—albeit potentially incomplete, if not all encounters arise. Research in animals, therefore, has emphasized that rank judgments depend critically on knowledge acquired through learning, coupled with a highly developed capacity for transitive inference (i.e., if A > B and B > C, then A > C) (Byrne and Bates, 2010, Cheney and Seyfarth, 1990, Grosenick et al., 2007, Paz-Y-Miño C et al., 2004).

Although existing work in humans (Kumaran et al., 2012), complemented by research in animals (Noonan et al., 2014, Rushworth et al., 2013), has provided evidence that the amygdala and anterior hippocampus are selectively involved in the acquisition and use of knowledge about a social (i.e., as opposed to a non-social (Kumaran et al., 2012) hierarchy, three important issues remain open. First, what are the neural computations that support the learning of social hierarchies? Although a recent line of research demonstrates that certain aspects of social learning (e.g., about traits, trust games, and theory of mind [ToM]) can be accounted for by reinforcement learning (RL) mechanisms (Behrens et al., 2009, Burke et al., 2010, Hackel et al., 2015, Hampton et al., 2008, King-Casas, 2005, Suzuki et al., 2012), a rich theoretical framework formalizes the acquisition of structured knowledge (e.g., about social hierarchies and networks) in Bayesian terms (Kemp and Tenenbaum, 2008, Tervo et al., 2016). Which accounts better for social hierarchy learning is not clear. Second, previous work has only examined the neural substrates underlying learning about social hierarchies composed exclusively of other individuals (Chiao et al., 2008, Kumaran et al., 2012, Zink et al., 2008). A key question, therefore, is whether learning about dominance relationships within one’s own hierarchy—arguably the most relevant type of knowledge in the real world—recruits similar or distinct neural mechanisms. Indeed, this has broader relevance for the fundamental question of whether self-related information is represented by distinct neural mechanisms (Amodio and Frith, 2006, Denny et al., 2012, Mitchell et al., 2006), an issue that has been difficult to answer definitively because of the natural intertwining of the self/other dimension with the richness and quantity of prior knowledge (e.g., in trait judgment tasks). Third, is there automatic generation of neural signals reflecting rank in a social hierarchy (hereafter termed “power” because it is considered a continuous dimension in our study) that was learned through experience, even when the task does not require it? Although previous work (Kumaran et al., 2012) demonstrated that neural signatures of power are generated when needed to perform an evaluation task, ecological evidence suggests that the power of others should be automatically represented, in an analogous fashion to perceptual signals relating to trustworthiness (Engell et al., 2007, Todorov et al., 2008, Winston et al., 2002).

**Figure 1:**
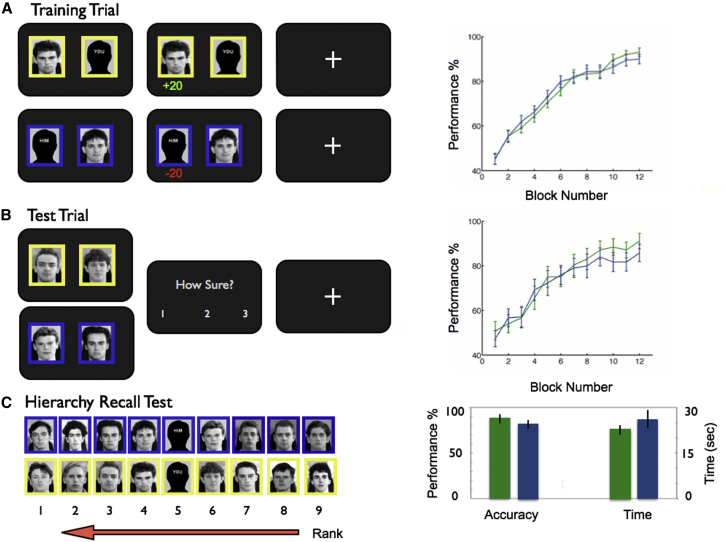
Learn Phase: Experimental Task and Behavioral Data (A) Training trials: timeline (left), behavioral data (right). Participants viewed adjacent items in the hierarchy: P4 versus P5 illustrated for Self condition (above, yellow border around faces) and Other condition (below, blue border around faces). Yellow or blue (counterbalanced) signified the logo color of the company to which individuals belonged. P5 was the participant or a close friend for the Self and Other conditions, respectively. Participants selected the item they thought had more power and received corrective feedback. Male participants saw only male individuals; female participants saw only female individuals. Right: training trial performance across all 12 experimental blocks, averaged across all eight training trial types (e.g., P1 versus P2, P2 versus P3) and participants (Self condition: green; Other condition: blue; error bars reflect SEM). (B) Test trials: timeline (left), behavioral data (right). Participants viewed non-adjacent items in the hierarchy (P3 versus P6 illustrated), inferred the higher ranking item, and rated their confidence in their choices; no feedback was provided. Right: performance over all 12 experimental blocks, averaged across all eight test trial types (four of which included the participant or his or her friend [P2 versus P5] and four of which did not [e.g., P3 versus P6]) and participants (Self condition: blue; Other condition: green; error bars reflect SEM). (C) Hierarchy recall test (debriefing session): pictures of the sets of people in the Self and Other hierarchy conditions were presented to participants, and they were asked to rank them in terms of their order in the hierarchy, with their performance timed. Example Self and Other hierarchies are illustrated (not shown to participants): members of the yellow- and blue-logo companies, respectively. Note that the allocation of person to rank position (1 = high rank, 9 = low rank) was randomized across participants, although P5 was always the participant or a close friend for the Self or Other condition. Right: performance (%) on hierarchy recall test and time taken (seconds) (Self condition: green; Other condition: blue).

To examine these issues, we developed a new experimental task that builds on a prior study (Kumaran et al., 2012) used to elucidate the neural substrates of hierarchy learning but incorporates extra features. In the “Learn” phase of the task, participants acquired knowledge of two social hierarchies in parallel. Although both hierarchies were comprised of unfamiliar members of two fictitious companies, they were distinguished by incorporating either the participants themselves (hereafter the Self hierarchy) or close friends of the participants (hereafter the Other hierarchy) (see Figure 1). Thus, our experimental design afforded us the opportunity to identify putative differences in the neural mechanisms that support learning about self-related information, decoupled as far as possible from many forms of preexisting and prior knowledge about the self that are inherent in other studies (Denny et al., 2012, Jenkins et al., 2008, Kelley et al., 2002, Macrae et al., 2004, Mitchell et al., 2005, Mitchell et al., 2006, Tamir and Mitchell, 2011, Tamir and Mitchell, 2012). We incorporated a direct test of the effectiveness of our Self/Other manipulation using a specifically tailored version of the classic implicit association test (IAT) (Greenwald et al., 1998, Mitchell et al., 2006) (see below and Supplemental Experimental Procedures). Finally, during a second scanning (“Categorization”) phase, participants viewed pictures of individuals from both hierarchies, allowing us to probe the underlying representations of the hierarchies learned in the previous phase and identify regions that automatically generate signals of power, even when the task does not require it.

In sum, our experiment was specifically set up to elucidate the computational mechanisms operating during social hierarchy learning, examine whether distinct neural processes support the learning and representation of self-relevant information (i.e., the power of individuals within one’s own, compared with another’s, social group), and determine whether signals reflecting individuals’ power are automatically generated even in the absence of task demands.

## Results

### Learn Phase

During the Learn phase, participants completed training trials in each of which a pair of adjacent people in the hierarchy was presented (e.g., P1 versus P2, where P = person; Figure 1): they were required to learn through trial and error which person had more power in each of two hierarchies relating to different companies signified by colored logos (i.e., yellow and blue; each company consisted exclusively of people of the same gender as the subject). One hierarchy included the subject himself or herself (the Self condition), and the other incorporated a close friend (the Other condition; see Supplemental Experimental Procedures for the elicitation procedure). Our experimental design incorporated a close friend in the Other condition, rather than an acquaintance or unfamiliar other individual, in order to render these conditions as similar as possible, thereby isolating the Self/Other dimension (e.g., Mitchell et al., 2006). Following each training trial block, participants completed test trials in which they were required to select the more powerful of the two items presented (e.g., P4 versus P6; Figure 1B) and rate their confidence in their decisions on a scale ranging from 1 (guess) to 3 (very sure). Test trials, therefore, differed from training trials in two critical ways: non-adjacent items in the hierarchy were presented during test trials (e.g., P4 versus P6), and no corrective feedback was issued (although subjects knew that they would ultimately be remunerated for correct answers). Confidence judgements did not attract compensation. Importantly, participants could not rely on memorization (i.e., of the item from each training pair associated with the positive outcome) to perform successfully during test trials but instead were required to deduce the correct item using knowledge of the underlying hierarchy.

### Behavioral Results

Participants improved their performance on training and test trials over the course of the Learn phase: no significant difference was found between Self and Other conditions in terms of reaction times (RTs) (Self: 1.49 [0.04] s and 1.52 [0.04] s; Other: 1.49 [0.04] s and 1.54 [0.05] s; training and test trials respectively, SEM in brackets), the correctness of choices, or confidence ratings (all p values > 0.2; Figures 1A and 1B). Following scanning, participants were also tested on their explicit knowledge of the hierarchy (hierarchy recall test; see Figure 1C and Experimental Procedures). They performed proficiently (Figure 1C), with no significant difference between conditions in terms of accuracy (Self versus Other: 92.1% [2.7%] versus 84.1% [4.4%], mean with SEM in brackets) or time taken (Self versus Other: 22.7 s [2.1 s] versus 25.6 s [3.1 s], mean with SEM in brackets) (both p values > 0.2).

**Figure 2:**
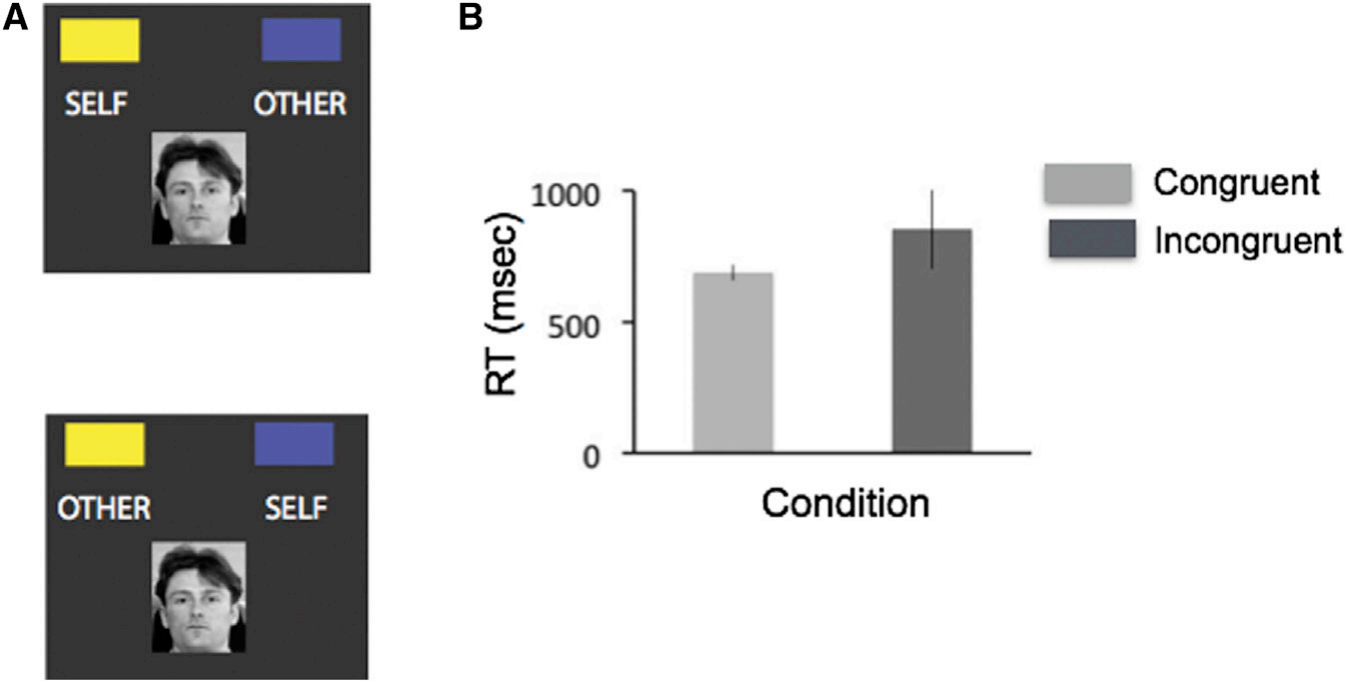
Implicit Association Test: Evidence that Participants Incorporated Themselves into the Self Hierarchy (A) Experimental design. Top: congruent condition: when the yellow logo (i.e., the color of the Self company logo in this example) is displayed on the same side as the word “Self” and when the blue logo (i.e., the color of the Other company) is displayed on the same side as the word “Other.” Bottom: incongruent condition: when the yellow logo is displayed on the side of the word “Other.” The rationale is that if participants have incorporated themselves into their own social group, they should be faster to categorize faces according to company membership in the congruent (cf. incongruent) condition, in which the word “Self” is on the same side as the color of the company logo (i.e., yellow). In contrast, RTs should be slower in the incongruent condition, in which the yellow logo is displayed on the side of the word “Other,” because of a Stroop-like effect (see Supplemental Experimental Procedures). Note that the words “Self” and “Other” were not presented to participants during the experiment: profile pictures were denoted by “you” and “him” or “her.” (B) Mean latencies for the congruent (light gray) and incongruent (dark gray) trials, averaged across participants (see Supplemental Experimental Procedures for details of analytic procedure). The IAT effect is the difference in response times between congruent trials and incongruent trials (error bars reflect SEM).

Following scanning, participants also completed a version of a classical psychological paradigm, the implicit association test (see Figures 2 and S1 and Supplemental Experimental Procedures) (Greenwald et al., 1998), which we adapted to probe the extent to which participants incorporated themselves into the Self hierarchy condition. There was a highly significant IAT effect, evidenced by speeding of RTs in congruent trials (congruent: 690 ms [152.8 ms]; incongruent: 856 ms [30.2 ms]; t_23_ = 5.33, p < 0.001; Figure 2; see Supplemental Experimental Procedures). This evidence was complemented by related subjective measures obtained through a debriefing questionnaire, in which participants reported that they incorporated themselves in the Self condition and their friends in the Other condition, to a similar degree (p > 0.1) (see Table S8). These results demonstrate the effectiveness of our experimental manipulation in the Self versus Other condition, consistent with previous evidence that merely assigning participants into different groups in an arbitrary fashion can have substantial effects on behavior (i.e., higher monetary sharing within a group, compared with across groups, in Volz et al., 2009).

### Computational Modeling

Existing models of transitive inference have been typically been restricted in being able to learn only relatively small hierarchies (i.e., six or fewer items) (Frank et al., 2003, von Fersen et al., 1991). We therefore developed two novel models capable of learning long hierarchies (i.e., here of nine items): one involving (approximate) Bayesian inference and the other involving reinforcement learning (see Supplemental Experimental Procedures).

According to the first model, subjects treat the powers of individuals as a hidden or latent variable, about which they make approximate Bayesian inferences. These inferences are based on the likelihood of observations (i.e., the outcomes of training trials that reveal which individual has more power). Given the finding that participants required approximately 200 trials to achieve proficiency (see Figures 1A and 1B), despite receiving deterministic feedback during training, we modeled them as having imperfect memory (as might, for instance, arise from a changing environment). For a concrete implementation of forgetful Bayesian inference, we used an example of a popular class of filtering algorithms known as sequential Monte Carlo (SMC) methods (Doucet et al., 2000). These aim to infer the underlying distribution of an evolving hidden variable, representing it through a set of notional samples or particles. Forgetfulness is straightforward to capture via a parameter (called σ) in the SMC model, which influences the tendency for particles to drift over time (see Supplemental Experimental Procedures). This prevents asymptotic certainty and slows learning.

The second method involved RL. Typical RL methods that assign values to items on the basis of their propensity to be associated with a rewarding outcome (e.g., the Rescorla-Wagner rule or Q learning; Watkins and Dayan, 1992) are known to fail in hierarchy learning tasks. This is because each item (apart from the top and bottom ranked) is equally associated with positive and negative outcomes during training trials. Consequently, in developing an RL account (hereafter termed RL-ELO) capable of successful hierarchy learning performance, we sought inspiration from algorithms used to update player ratings in games (e.g., Yliniemi and Tumer, 2013) (e.g., the ELO rating system in chess; see Supplemental Experimental Procedures), whose critical component is to increase or decrease the rating (i.e., power) of the winning or losing individual in a pairwise contest or trial as a function of the rating of one’s opponent (i.e., the winner has a relatively small update if the opponent was estimated to be much less powerful). This has been proved to work even in gargantuan problems.

A critical qualitative difference between the SMC and RL-ELO schemes concerns uncertainty. The SMC model inherently models the uncertainty in the estimation of power. In contrast, the RL-ELO, like temporal difference (TD) learning, maintains only a single scalar estimate of power for each individual (e.g., Niv et al., 2006, Schultz et al., 1997; although see Gershman, 2015). Furthermore, the models differ in the nature of the mechanism by which they update their estimates of the power of individuals within the hierarchy (see below).

One principal aim of this study was to determine which of these two models, the SMC mechanism or the RL-ELO mechanism, was better able to capture participants’ data. At a behavioral level, we quantified the fit of each of the SMC and RL-ELO models, as well as previously developed models (value transfer and Rescorla-Wagner), to participants’ choice behavior during training and test trials. Using a maximum likelihood estimation procedure to optimize a separate set of parameters for each participant (see Daw, 2011, Wimmer et al., 2012), we found strong evidence (see Table 1) in favor of the SMC model according to the log likelihood of each model and the corresponding Bayesian information criterion (BIC) measure (Raftery, 1995). We also examined a variant of the RL-ELO model, termed RL-ELO_F_, which incorporated an extra parameter (i.e., σ) to allow forgetting (i.e., through the addition of Gaussian noise at each time step); this did not significantly improve the fit of the RL model indexed by BIC scores (see Table 1).

Interestingly, the difference between our two primary models arose in the trials in the first half of the experiment (BIC = 185.0 versus 201.8 for SMC versus RL-ELO) rather than the second half (BIC = 109.3 versus 111.7, respectively), consistent with the notion that the SMC model captures behavior more effectively than the RL model when participants are more uncertain about the relative power of individuals during the early phase of learning. This is explained by a qualitative difference between the updating mechanisms of the models: the RL-ELO model only updates the values of the current items in a trial (i.e., unconstrained by the values of the other items). In contrast, the SMC model naturally updates the values of items not present in a given training trial, because it updates a posterior distribution over all items on a trial-by-trial basis, because each particle constitutes a particular belief about the values of all nine items in the hierarchy. The reason that the difference in model fit is greatest in the first half of the experiment is that this is the period when most learning and hierarchy updating is occurring (e.g., see Figure 1). This difference in updating mechanism makes the specific prediction that the RL-ELO model should be much more sensitive (i.e., in terms of influence on its predicted choices) than the SMC model to the particular reinforcement history of items and therefore trial order experienced. We provide evidence for this predicted difference (see Figure S2: a significantly greater effect of reinforcement history on negative log likelihood [NLL] for the RL [cf. SMC] model, *Z* = 24.8, p < 0.0001). Notably, the difference in updating mechanism also results in the SMC model’s being able to more rapidly converge on the correct rank ordering: the difference between model fits (i.e., in terms of negative log likelihood) showed a highly significant correlation with subjects’ performance, with higher performing subjects showing greater advantage for the SMC model (Pearson’s correlation: R = −0.75 and −0.79 [p < 0.0001] for the Self and Other conditions). Notably, the vast majority of subjects were better fit by the SMC model (22 and 23 of 28 subjects in the Self and Other conditions).

The task affords two additional measures by which the models can be compared: reaction times and confidence judgments. We expect these to be related to the uncertainty associated with the choices, captured by a trial-by-trial internal variable common to both SMC and RL-ELO models, namely, choice entropy (see Supplemental Experimental Procedures). A linear mixed-effects model demonstrated that the SMC model also provided a superior fit to participants’ reaction time data, compared with the RL-ELO model during both training (SMC, BIC = 8,492; RL-ELO, BIC = 8,699) and test (SMC, BIC = 4,491; RL, BIC = 4,520) trials, in which a choice entropy was entered as a fixed effect, and participant and condition (Self or Other) were entered as random effects (likelihood ratio compared with the null model, all p values < 1 × 10^−15^). The choice entropy from the SMC model also captured the confidence of participants in their responses during transitive test trials more proficiently (SMC, BIC = 8,687; RL-ELO, BIC = 8,733; likelihood ratio versus null model, all p values < 1 × 10^−15^).

### Neuroimaging Data

#### Neural Activity in the Amygdala and Anterior Hippocampus Correlates with SMC-Modeled Difference between the Power of Individuals during Test Trials

**Figure 3:**
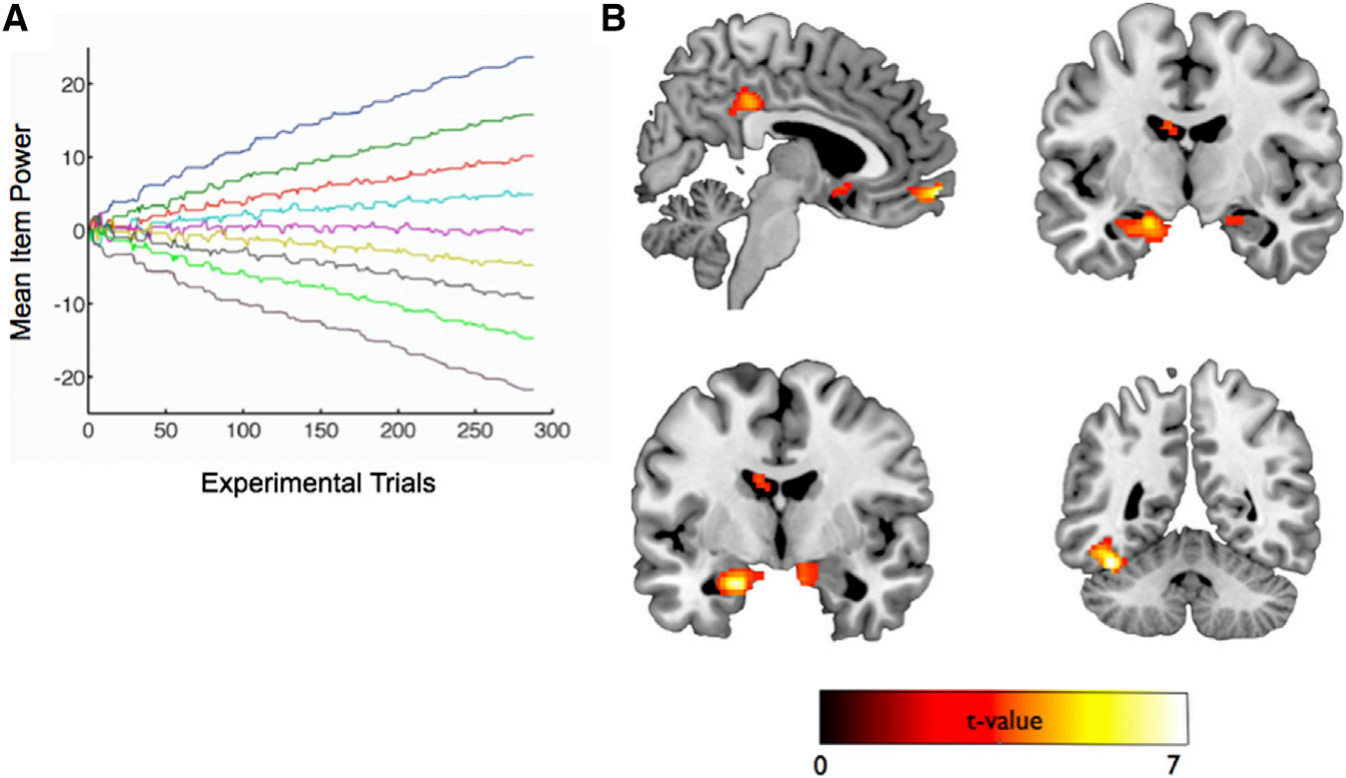
Learn Phase: Neural Activity in the Amygdala, Hippocampus, and vMPFC Correlates with SMC-Estimated Difference in Individuals’ Power during Test Trials in Self and Other Conditions (A) Illustrative plot from a participant showing the evolution over the experiment of the mean (i.e., expectation over the distribution of) power relating to each of the nine individuals in the hierarchy. (B) Activity in the bilateral amygdala (top right), vmPFC and posterior cingulate cortex (top left), bilateral anterior hippocampus (bottom left), and region proximate to the fusiform face area (bottom right) shows a significant correlation with SMC-modeled absolute difference between individuals’ power in test trials. Activations are thresholded at p < 0.005 uncorrected for display purposes but significant in all regions at p < 0.001 uncorrected and p < 0.05 whole-brain FWE corrected at peak or cluster level. See Table S1 for a full list of activations.

Given our finding that the SMC model best accounts for participants’ behavior (i.e., choices, RT, confidence data), we next probed the neural data using its key internal variables. We first focused on the test trial data, in which participants were presented with item pairs not seen during training trials (e.g., P3 versus P6), were not given corrective feedback, and were therefore required to use their estimates about individuals’ power. We sought to identify regions where neural activity tracked the (expectation over the) difference between the modeled distributions of the power of items in a given trial (see Figure 3A). To achieve this, we created participant-specific trial-by-trial parametric regressors reflecting the unsigned power difference between items which were used to regress against the fMRI data (see Supplemental Experimental Procedures).

We found a robust correlation between neural activity in the amygdala, hippocampus, ventromedial prefrontal cortex (vMPFC), and the difference between the power of individuals as estimated by the SMC model (see Figure 3B and Table S1). We also observed a tight correlation between neural activity and item power difference in a region proximate to the fusiform face area (FFA) (see Figure 3B). Results from an region-of-interest (ROI) analysis performed separately in the Self and Other conditions provide evidence that the left amygdala (Self: t_27_ = 1.95, p = 0.028; Other: t_27_ = 1.73, p = 0.044), ventromedial prefrontal cortex (Self: t_27_ = 2.27, p = 0.01; Other: t_27_ = 3.14, p = 0.0013), and FFA-proximate region (Self: t_27_ = 2.75, p = 0.005; Other: t_27_ = 3.33, p = 0.0011) code SMC-modeled differences in power that support performance during test trials in both conditions. Notably, these effects cannot be driven by differences in reaction times between trials given that an earlier regressor in the same general linear model captured such effects (see Experimental Procedures). Together with a previous study (Kumaran et al., 2012), these findings provide evidence that the anterior hippocampus and amygdala play a specific role in social rank judgements: indeed, activity in this region identified in this previous study to be associated with model-agnostic measures of hierarchy learning (i.e., social > non-social contrast, shown in Figure 2B of Kumaran et al., 2012; ROI defined at p < 0.005 uncorrected) showed a robust correlation with the SMC-modeled difference in power during test trials in the present study (t_27_ = 6.17, p < 0.000001).

#### Hierarchy Updating: The SMC Model Provides a Superior Fit to Neural Data Compared with the RL Model

**Figure 4:**
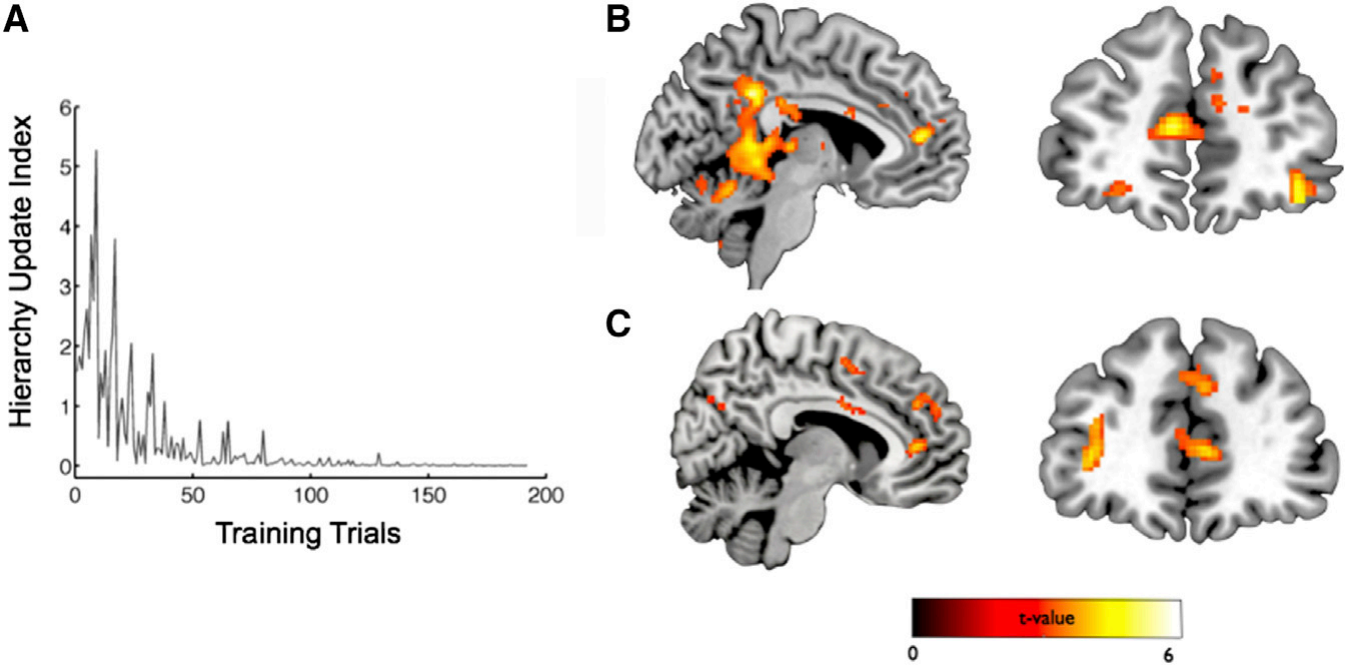
Learn Phase: MPFC Activity Correlates with SMC-Modeled Hierarchy Update Signal Selectively in Self Condition (A) Illustrative example from one subject showing profile of SMC-modeled hierarchy update index during training trials (see Supplemental Experimental Procedures for details). (B) Whole-brain analysis: significant correlation between activity in MPFC and hierarchy update index in Self condition (sagittal and coronal views: MNI x, y, and z coordinates −6, 46, and 12; *Z* = 4.22, p < 0.001 uncorrected and SVC p = 0.0040) (Table S3B). (C) Significantly greater correlation between MPFC activity and updating of objective measure of hierarchy knowledge (i.e., negative log likelihood of responding correctly on all possible pairs of individuals) in Self compared with Other condition: sagittal (left) and coronal (right) sections shown (MPFC x, y, and z coordinates 2, 44, and 8; *Z* = 3.87, p < 0.001 uncorrected and SVC p = 0.021). Display threshold is p < 0.005 uncorrected.

Previous results provide evidence for shared signals in the amygdala and hippocampus during performance of test trials, relating to both one’s own group (Self condition) and another’s group (Other condition). We next turned our focus to training trials, in which participants had the opportunity to update their beliefs about the power of individuals in the Self and Other hierarchies (i.e., on the basis of corrective feedback): this provided the basis of successful performance in test trials. To identify signatures of learning, we sought regions in which neural activity showed a correlation with an internal measure, termed the hierarchy update index (Figure 4A) (see Supplemental Experimental Procedures) reflecting the degree to which participants updated their estimates of the power of individuals from trial to trial. Specifically, for each pair of items (e.g., P1 versus P2) we computed the Kullback-Leibler divergence between the participants’ estimates of the probability of one item winning against the other before and after feedback, summing this quantity across all pairs (see Supplemental Experimental Procedures). Note that the correlation between the hierarchy update index and other measures (e.g., chosen power) was relatively low (∼0.2).

In a whole-brain analysis collapsed across both Self and Other conditions, we found a robust correlation between MPFC activity and the SMC-modeled hierarchy update index (MPFC: x, y, and z coordinates −8, 44, and 10; *Z* = 5.04, family-wise error peak level corrected p = 0.022) and also with the hippocampus and the FFA-proximate region (see Table S3A). We also identified significant correlations in a targeted ROI analysis in the MPFC (t_27_ = 1.97, p = 0.030), left amygdala (t_27_ = 1.73, p = 0.047), bilateral hippocampus (left: t_27_ = 3.70, p = 0.00048; right: t_27_ = 2.77, p = 0.005), and FFA-proximate region (t_27_ = 3.28, p = 0.0014) but not the vmPFC (p > 0.1). When the Self and Other conditions were considered separately, significant effects were present in the hippocampus (Self: left hippocampus t_27_ = 3.10, p = 0.0022, right hippocampus t_27_ = 2.94, p = 0.0033; Other: left hippocampus t_27_ = 1.93, p = 0.032).

Previously we showed that the SMC model fit the behavior more proficiently than the RL-ELO model and provided evidence of their qualitative difference in hierarchy-updating mechanism (e.g., see Figure S2). Here we compare the fit of these models to the neural data, using model-derived hierarchy update regressors, collapsed across Self and Other conditions, in regions of interest defined anatomically and functionally (see Supplemental Experimental Procedures) (Ashby and Waldschmidt, 2008, Niv et al., 2015, Wilson and Niv, 2015). The relative differences in BIC between models (Ashby and Waldschmidt, 2008, Raftery, 1995) provide strong support that the SMC model best captures neural activity in amygdala, hippocampus, and MPFC (see Table S2).

#### Activity in the MPFC Correlates with SMC-Modeled Hierarchy Update Signal in the Self Condition during Training Trials

We found that the correlation between MPFC activity and the hierarchy update index was driven by the Self condition (whole-brain analysis: MPFC: x, y, and z coordinates −6, 46, and 12; *Z* = 4.22, p < 0.001 uncorrected and small-volume corrected [SVC] p = 0.0040; MPFC ROI analysis, t_27_ = 2.73, p = 0.0055; Figure 4B and Table S3B). This finding remained robust when we restricted our analyses to just those trials in which participants updated their knowledge about other individuals in the hierarchy, excluding themselves and their friends (i.e., P4 versus P5, P5 versus P6; see Supplemental Experimental Procedures) (whole-brain analysis: Self: Montreal Neurological Institute [MNI] x, y, and z coordinates −6, 46, and 12; *Z* = 4.25, p < 0.001 uncorrected and SVC p = 0.0030; MPFC ROI analysis, t_27_ = 2.93, p = 0.0034). No significant correlations were observed in the MPFC in these analyses in the Other condition (ROI analyses: p values > 0.2). Further, an ROI analysis showed that there was a greater correlation between MPFC activity and the hierarchy update index in the Self compared with the Other condition (Self > Other: t_27_ = 1.83, p = 0.039), with no significant differences found in this analysis in other regions (i.e., hippocampus, amygdala, and FFA-proximate region: all p values > 0.1). No significant differences were found in the reverse contrast (i.e., Other > Self) in either a whole-brain analysis or ROI analysis (all p values > 0.2).

Furthermore, we found a robust between-subjects correlation between performance (i.e., averaged across this experimental phase) and the strength of the correlation between MPFC activity and the hierarchy update index in the Self condition. This was the case at the whole-brain level in the MPFC in the Self condition (x, y, and z coordinates −8, 42, and 8; p < 0.001 uncorrected and SVC p = 0.043) and in the MPFC ROI (t_27_ = 2.07, p = 0.024; trend in L amygdala ROI t_27_ = 1.61, p = 0.06). No such correlation was found in the MPFC ROI in the Other condition, with a significantly greater correlation in the Self condition (Self > Other: *Z* = 1.85, p = 0.032).

This hierarchy update analysis was based on participants’ subjective estimates of the power of individuals at a given moment: we also derived an analogous measure capturing the trial-by-trial change in participants’ objective knowledge of the ground truth hierarchy. Although this quantity cannot be directly computed by participants, it was highly correlated with the hierarchy update index (i.e., ∼0.8) and yielded robust differences between the Self and Other conditions in the MPFC in a whole-brain analysis (Self > Other: whole-brain analysis MPFC x, y, and z coordinates 2, 44, and 8; *Z* = 3.87, p < 0.001 uncorrected and SVC p = 0.021, see Figure 4C: MPFC ROI analysis t_27_ = 3.56, p = 0.00064). Of note, no significant correlations with this objective hierarchy update index were found in the MPFC in the Other condition (ROI analyses: p values > 0.2).

#### Selective Coupling between MPFC and Amygdala and Hippocampal Activity during Updating of Hierarchy Knowledge in the Self Condition

Our results show that neural activity in the MPFC specifically correlates with updating of hierarchy knowledge in the Self condition, with significant effects in the amygdala and hippocampus observed across both Self and Other conditions. We next asked whether neural activity in this part of the MPFC and the amygdala and hippocampus, regions that are thought to be anatomically connected (Beckmann et al., 2009, Carmichael and Price, 1995), exhibit greater functional coupling during updating of hierarchy knowledge in the Self compared with the Other condition.

To do this, we performed a psychophysiological (PPI) analysis (Friston et al., 1997, O’Reilly et al., 2012) (see Supplemental Experimental Procedures for details): this was specifically set up to ask in which brain regions the magnitude of functional coupling of neural activity with the MPFC shows a significantly greater correlation with the amount by which hierarchy knowledge changes in the Self compared with the Other condition, above and beyond that explained by differences in the basic correlation between the hierarchy update index in the Self and Other conditions (i.e., hierarchy update: Self > Other, as reported in the preceding analysis). We observed significant effects in the amygdala and hippocampus both in a whole-brain analysis (see Table S4) and in targeted ROI analyses (left hippocampus t_27_ = 2.35, p = 0.010, right hippocampus t_27_ = 1.65, p = 0.051; left amygdala t_27_ = 2.14, p = 0.020, right amygdala t_27_ = 2.00, p = 0.028). These results provide evidence for selective coupling between the MPFC and the amygdala and hippocampus during updating of hierarchy knowledge in the Self condition.

#### Correlation between Neural Activity in Amygdala, Hippocampus, and MPFC and SMC-Modeled Power of Chosen Individual during Training Trials

**Figure 5:**
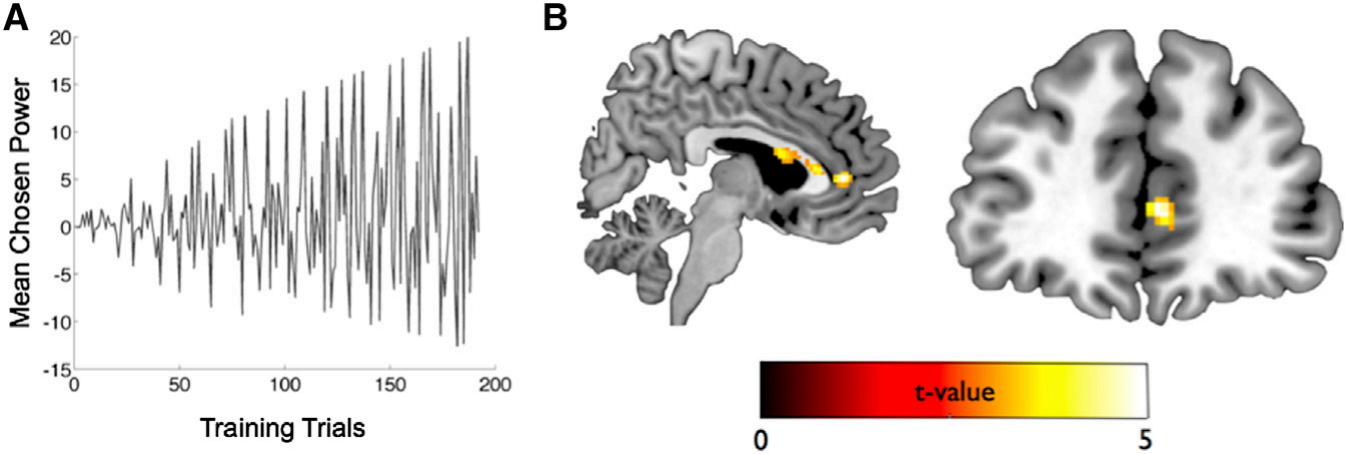
Learn Phase: Correlation between Neural Activity in the MPFC and SMC-Modeled Chosen Power during Training Trials: Self versus Other (A) SMC-modeled (expectation over) posterior distribution of chosen power for illustrative subject. (B) Results of whole-brain analysis: region of MPFC identified by correlation of neural activity with SMC modeled chosen power: Self > Other (MNI x, y, and z coordinates: 6, 42, and 4; *Z* = 4.23, significant at SVC p = 0.001 and p < 0.001 uncorrected; see Table S6D). Display threshold is p < 0.005 uncorrected.

Having established that neural activity in the MPFC correlates with updating of hierarchy knowledge in the Self condition, we next asked whether another internal variable of the SMC model, specifically, the expectation of the distribution (i.e., mean) of power of the chosen item (Figure 5A), was reflected in neural activity (see Supplemental Experimental Procedures, Table S5, for a separate analysis relating to another internal variable: the entropy over item pairs). We first performed an analysis collapsed across Self and Other conditions (i.e., main effect): ROI analyses provided evidence that chosen power was represented in the amygdala (left: t_27_ = 2.62, p = 0.0071; right: t_27_ = 1.75, p = 0.046) and ventromedial prefrontal cortex (t_27_ = 3.19, p = 0.0018) (see Table S6A for results of whole-brain analysis).

We also observed significant differences between the Self and Other conditions in terms of the correlation of neural activity with chosen power. In a whole-brain analysis, we found that neural activity, in a similar region of MPFC to that revealed by the hierarchy update index analysis above, was significantly correlated with trial-by-trial chosen power in the Self condition (MNI x, y, and z coordinates 4, 44, and 2; *Z* = 3.01, SVC p = 0.037; see Table S6B) but not the Other condition (see Table S6C). Moreover, there was a significant difference between the Self and Other conditions in the MPFC (see Figure 5B and Table S6D). Furthermore, this finding remained robust when we restricted our analyses to only those trials that did not involve the participant or his or her friend (Self > Other: MNI x, y, and z coordinates 6, 42, and 4; *Z* = 4.31, p < 0.001 uncorrected and SVC p = 0.001). No region showed significant differences in the reverse contrast (i.e., Other > Self).

Similarly, in an ROI analysis based on this region of the MPFC, we found significant differences between the Self and Other conditions during test trials that did not involve the participant or his or her friend (see Supplemental Experimental Procedures). Specifically, we found evidence for a difference in the correlation between neural activity and the trial-by-trial SMC-modeled difference in individual’s power within the MPFC ROI (Self > Other t_27_ = 1.69, p = 0.050), though not within amygdala, hippocampal, vmPFC, and FFA-proximate ROIs (all p values > 0.1). Together, these analyses, by focusing only on trials that did not involve the participants themselves, suggest that the MPFC supports the updating and representation of power information about other individuals (i.e., rather than only oneself), when these individuals are part of our own social group rather than another group (i.e., Self > Other) (see Discussion).

### Categorization Phase

#### Behavioral Results

**Figure 6:**
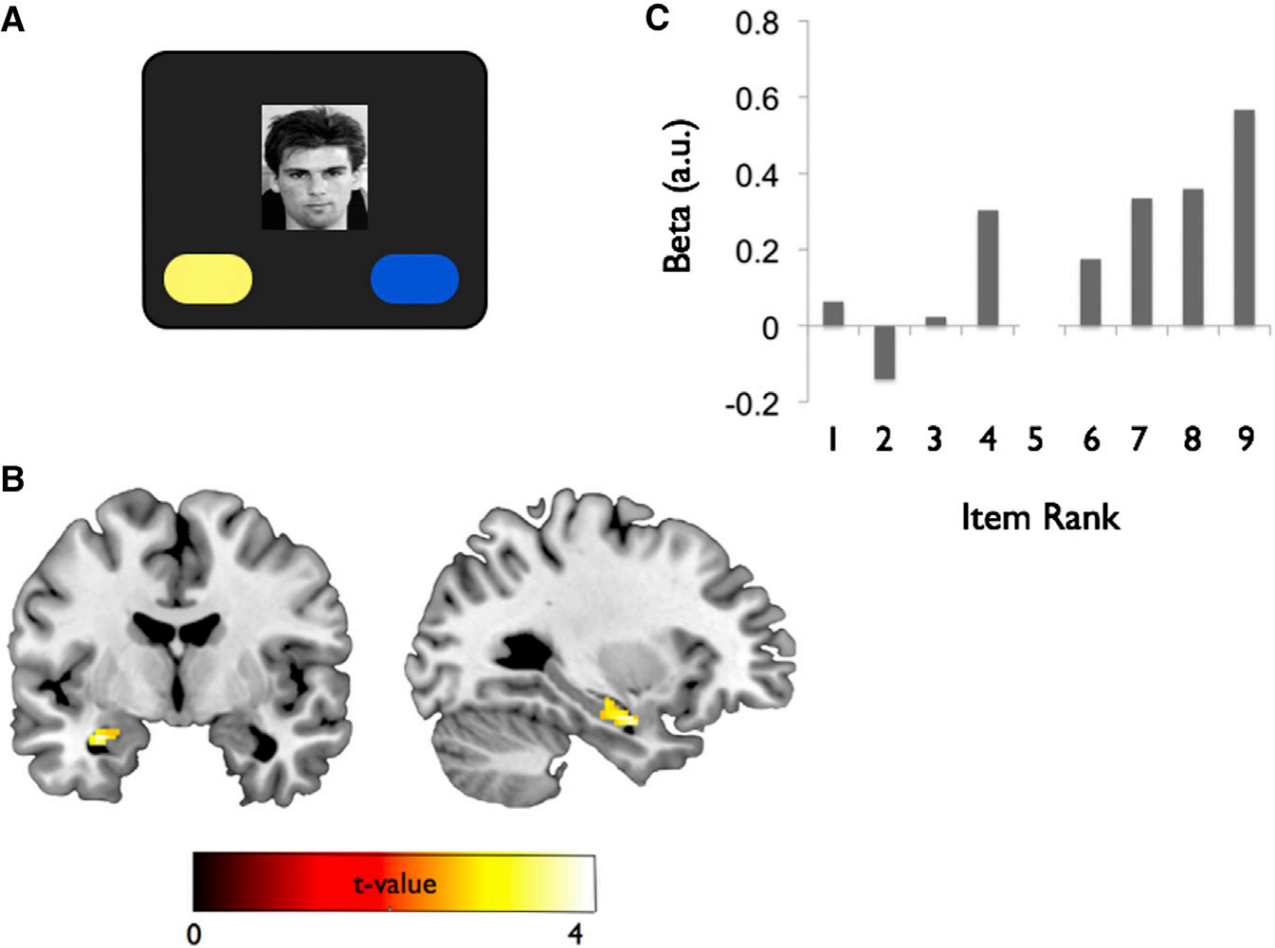
Categorization Phase: Amygdala and Anterior Hippocampus Automatically Generate Rank Signals: Linear Correlation with Neural Activity (A) Paradigm: during the Categorization phase, participants viewed individuals from the Self and Other hierarchies (with the exception of the profile pictures denoting themselves and their friends; each picture repeated four times) and categorized them according to the company to which they belonged (i.e., the company with the yellow or blue logo). (B) Activity in the left amygdala and anterior hippocampus shows a linear correlation with rank (main effect: Self and Other conditions). Display p < 0.005; significant at p < 0.001 uncorrected and L amygdala (SVC p = 0.025), and L hippocampus (SVC p = 0.034) (see Table S7). (C) Parameter estimates from peak voxel in main effect (i.e., collapsed across Self and Other conditions) L amygdala and hippocampus (see Table S7). Significant linear correlation evident, with lower ranks (i.e., where 9 = lowest) eliciting highest neural activity. Note that these plots were derived from an “illustrative” model (see Supplemental Experimental Procedures). Statistical inference, however, was based strictly on the parametric model (see Supplemental Experimental Procedures). Note that rank 5 is denoted by an empty slot, because the profile pictures denoting the participants themselves (Self condition) or their friends (Other condition) were not presented.

In the next phase of the experiment, we aimed to probe participants’ representations of the underlying hierarchies and to examine differences between those involving oneself versus a close friend. Participants completed an incidental categorization task that did not require the retrieval of information about power (Figure 6A), allowing us to ask whether signals relating to individuals’ power were automatically generated even in the absence of explicit task demands. Individual pictures of people in the Self and Other hierarchies (with the exception of the profile pictures of participants and close friends) were presented, and participants were required to determine to which company individuals belonged (i.e., the yellow or blue logo, assignment counterbalanced). Participants performed this categorization task accurately (Self: 81.8% [2.8%], Other 79.7% [2.96%]; Self reaction time: 0.80 s [0.010 s]; Other reaction time: 0.80 s [0.013 s]; all p values > 0.1). There was no difference in accuracy or RT as a function of rank (p > 0.1).

#### Neuroimaging Data

We first set up a parametric model to identify regions whose activity exhibited significant linear correlations with the rank of individual people in the true underlying hierarchy. We found that neural activity in the left amygdala and anterior hippocampus showed a significant positive correlation with rank across Self and Other conditions (Figures 6B and 6C; Table S7). This provides novel evidence that these neural structures automatically generate rank signals even when the task does not require it and complements studies showing obligatory processing of perceptual cues of trustworthiness (though not dominance), whereby less trustworthy faces elicit higher levels of amygdala activity (Todorov et al., 2011, Winston et al., 2002). No region showed a significant negative correlation with rank. The finding that less powerful (i.e., lower ranked) individuals elicited higher levels of activity is consistent with previous work examining signals relating to valence and dominance of faces based on physical appearance (Todorov et al., 2011), rather than associative learning (i.e., as in this study).

**Figure 7:**
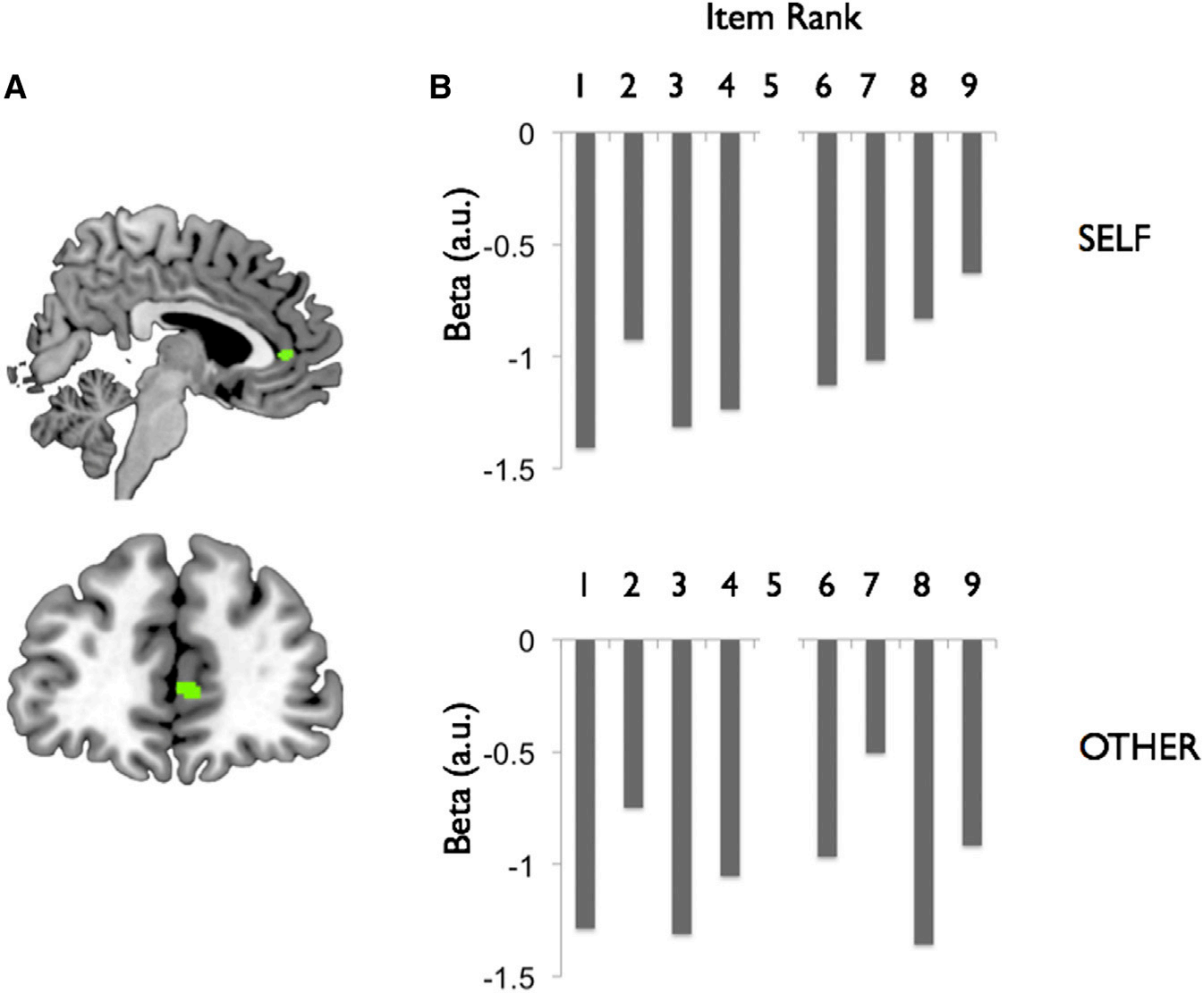
Activity in MPFC Shows a Linear Correlation with Rank in the Self Condition during the Categorization Phase (A) Region of MPFC defined on the basis of the results of a separate fMRI phase (i.e., learning; see Supplemental Experimental Procedures for details). (B) Parameter estimates averaged across MPFC ROI in the Self condition (above) and Other condition (below). A significant linear correlation between neural activity and rank in the Self but not the Other condition is evident. These plots were derived from an “illustrative” model; however, statistical inference was based strictly on the parametric model (see Supplemental Experimental Procedures). Note that rank 5 is denoted by an empty slot, because the profile pictures denoting the participants themselves (Self condition) or friend (Other condition) were not presented.

We also observed a significant linear correlation between rank and neural activity in the MPFC ROI derived from the Learn phase (t_27_ = 1.86, p = 0.036; Figure 7) in the Self condition, providing a parallel to the automatic valuation of items observed in the vmPFC (Lebreton et al., 2009). No such correlation was observed in the Other condition (p > 0.2). Although the difference between this linear correlation between Self and Other conditions was not significant in the MPFC ROI, we did observe a significant interaction when rank extremes were considered (i.e., Self/Other × top/bottom rank: t_27_ = 1.98, p = 0.029), reflecting the fact that MPFC activity distinguished between highest and lowest ranks selectively in the Self condition. No such effect was present in any of the other ROIs (amygdala, hippocampus, or vmPFC; all p values > 0.1).

## Discussion

Although social hierarchies are a fundamental organizing structure of primate social groups, little is understood about the computations underlying learning and also whether there are distinct neural mechanisms that support the ability of primates to judge the rank of others within their own social groups, compared with other social groups. To address these questions, we first developed two novel hierarchy learning models, one based on reinforcement learning (RL-ELO) and the other on approximate Bayesian inference (SMC). We showed that participants’ behavior and neural data were better captured by the Bayesian inference scheme, which inherently computes the uncertainty about estimates of power (i.e., rank in a continuous dimension), than by the uncertainty-insensitive RL model. We demonstrate that learning about one’s own social hierarchy, as opposed to that of a close friend, was associated with distinct correlations between internal variables of the SMC model and MPFC activity, while shared signals for both hierarchy types were present in the amygdala and hippocampus. Furthermore, we found that the MPFC was selectively engaged during updating of knowledge about one’s own hierarchy, a process that explained variance in participants’ performance, and involved functional interactions with the amygdala and hippocampus. Finally, we show that neural signals that automatically represent the power of individuals were generated by the hippocampus and amygdala even when the task did not require it, with power-related activity in the MPFC specific to self-relevant hierarchies.

Emerging evidence suggests work that RL models that do not maintain explicit estimates of uncertainty about decision variables are able to capture behavior and neural data in a wide range of settings, including experiments involving trust games (Hackel et al., 2015, King-Casas, 2005), theory of mind (Hampton et al., 2008), and inferring the preferences and actions of others (Burke et al., 2010, Suzuki et al., 2012). A recent study (Hackel et al., 2015) demonstrated that learning about the generosity of an individual on the basis of informative feedback reflecting his or her propensity to share a monetary endowment was well captured at the behavioral and neural levels by a reinforcement learning model. These findings raise the question of whether learning about the power of individuals in a social hierarchy—another trait-level characteristic like generosity, for which learning is typically feedback-based (i.e., ecologically through observation of the outcome of dyadic contests, mirrored experimentally by training trials in our task)—could also be mediated by an RL process maintaining scalar quantities. Our study, however, provides compelling evidence against this hypothesis. Specifically, we found that the SMC model provided a quantitatively better fit than the RL model across behavioral (i.e., choice, RT, and confidence data) and neural levels. Of course, this provides evidence for the key differentiating constructs underlying SMC, namely, forgetful, uncertainty-sensitive inference. Other approximate Bayesian implementations could lead to the same behavior and neural signals.

The current results dovetail with previous work that used a related paradigm to show that the anterior hippocampus and amygdala as identified here—regions that are coupled by massive bidirectional connectivity (Fanselow and Dong, 2010)—play a specific role in the acquisition of knowledge about social hierarchies (Kumaran et al., 2012). That study, however, was not able to investigate the computational mechanisms underlying learning; rather, neural activity in these regions was correlated directly with model-agnostic behavioral indices of learning. In contrast, our work implies that the anterior hippocampus and amygdala supports social hierarchy learning by maintaining and updating beliefs about the power of individuals through the operation of a probabilistic model, evidenced by significant correlations between neural activity in these structures and internal variables of the SMC model (e.g., the differences in power between individuals during test trials). Indeed, the higher order representation of the uncertainty in one’s beliefs naturally sustained by probabilistic models (compared with RL approaches) may be advantageous in deciding whether to approach or avoid an individual on the basis of estimated differences in power. Furthermore, our finding that the hippocampus supports such model-based representations of social hierarchies connects with the notion that these structures constitute a cardinal example of relational knowledge of the environment that can be flexibly accessed (e.g., for reasoning or recall) (Cohen and Eichenbaum, 1994, Eichenbaum, 2004). How this type of hippocampal representation for structural forms (i.e., hierarchies) accrued across multiple experiences is compatible with a key role for the hippocampus in supporting memory for individual episodes constitutes an important area for future research (Eichenbaum, 2004, Kumaran and McClelland, 2012, Zeithamova et al., 2012).

Our experimental design was specifically configured to address a key question that has not been explored in previous studies, namely, whether learning about a hierarchy of which one is a part recruits similar or distinct neural mechanisms than learning about a hierarchy that is composed entirely of other individuals. The difference had notable behavioral signatures, both in the congruency RT effect observed in the IAT and from information obtained at debriefing, which suggested that the participants found the scenario naturalistic. At a neural level, in contrast to the shared representations for hierarchies in the Self and Other condition in the amygdala and anterior hippocampus, we found signatures of MPFC activity that were selective to the Self condition, a finding that cannot be attributed to differences in performance, because this was comparable across conditions. Specifically, MPFC activity showed a robust correlation with the degree to which participants updated their knowledge of the hierarchy from trial to trial. The relevant area of the MPFC was the only region to show a significantly greater correlation between neural activity and this SMC model-derived hierarchy update signal in the Self condition than the Other condition (i.e., no significant difference was observed in the amygdala or hippocampus). Furthermore, we found evidence for a selective coupling between the MPFC and the amygdala and hippocampus during updating of hierarchy knowledge in the Self condition, consistent with the anatomical connectivity between these regions (Beckmann et al., 2009, Carmichael and Price, 1995). A nearby MPFC region also exhibited significantly greater correlation between neural activity and SMC model-estimated chosen power in the Self compared with the Other condition (i.e., in both training and test trials). Interestingly, this dissociation between the amygdala and anterior hippocampus and the MPFC was also evident under very different circumstances: when participants were performing an incidental categorization task (i.e., scanning phase 2: Figures 6 and 7). Even though information concerning the power of individuals was irrelevant in this setting, we observed specific coding of self-related information about power, and domain-general coding of power, in the MPFC and amygdala and anterior hippocampus, respectively.

Our results, therefore, align with several strands of research that have implicated a similar ventral region of the MPFC in the representation and processing of self-relevant information (Adolphs, 2009, Denny et al., 2012, Jenkins et al., 2008, Kelley et al., 2002, Kumaran and Maguire, 2005, Macrae et al., 2004, Mitchell et al., 2005, Mitchell et al., 2006, Mobbs et al., 2009, Ochsner et al., 2004, Tamir and Mitchell, 2011, Tamir and Mitchell, 2012, Wittmann et al., 2016). This evidence has come from a range of studies: experiments in which participants are asked to judge the applicability of traits to themselves compared with others (Denny et al., 2012, Kelley et al., 2002, Mitchell et al., 2006), work suggesting that items subjected to self-related processing are afforded privileged status in memory (Macrae et al., 2004), and research on constructing imagined scenarios involving either oneself or others (De Brigard et al., 2015, FeldmanHall et al., 2012, Hassabis et al., 2014, Schacter and Addis, 2007). A natural constraint of this previous body of work (see Denny et al., 2012, for a meta-analysis), however, is that MPFC activity elicited in relation to self-attributions (e.g., trait judgments such as “am I trustworthy?”), compared with other-related judgments (e.g., “is Bill Clinton trustworthy?”), could reflect either differences in prior knowledge about oneself or instead unique aspects of self-related representation and processing. Critically, our experimental design allowed us to effectively isolate the learning and representation of self-related information from prior knowledge in two ways. First, the power of individuals in the Self and Other hierarchies was arbitrary and needed to be learned “from scratch,” rendering prior knowledge concerning oneself irrelevant. Second, our paradigm allowed us to demonstrate that the MPFC effects reported were robust to the exclusion of trials involving the “you” and “him or her” profile pictures (i.e., trials in which the participant or his or her friend was directly involved, such as the P5 versus P6 training trial), providing evidence that the MPFC represents the power of individuals in one’s own hierarchy, a dimension that is known to exert significant behavioral influences (Chang et al., 2011, Cheney and Seyfarth, 1990). Hence, our results provide compelling evidence that neural mechanisms operating in the MPFC are distinctive with respect to self-related information and accordingly suggest that self-relevant information may indeed be uniquely represented in the brain, with consequent implications for the regulation of cooperative and competitive interactions (Apps et al., 2016, Crockett et al., 2014, Seyfarth and Cheney, 2012).

It is interesting to relate our work to evidence suggesting that the anterior cingulate cortex gyrus (ACCg), a region that contains Brodmann areas 24 a/b and 32 (Apps et al., 2016) and whose anterior portion overlaps with the MPFC region identified in our study, makes an important contribution to social cognition (Apps et al., 2016, Rudebeck, 2006). An emerging perspective suggests that the ACCg plays a key role in facilitating cooperative and competitive interactions, by tracking parameters such as the value and cost of options to others (Apps et al., 2016, Chang et al., 2013). As such, neuronal activity in ACCg has been referred to as situated in an Other-centric reference frame, with neurons in this region coding specifically for reward receipt by the other individual in a dictator game, contrasting with the self-centric coding (i.e., for reward receipt by self) of neurons in other regions such as the orbitofrontal cortex (Chang et al., 2013). On the face of it, evidence for other-referenced coding in the ACCg runs contrary to our MPFC findings; in fact, our results are entirely consistent with this perspective. Specifically, we show that the MPFC supports the learning and representation of power information about other individuals, when these individuals are part of our group rather than another group (i.e., Self > Other). As noted previously, our findings were robust to the exclusion of trials in which participants themselves were involved. As such, our results suggest that the ACCg/MPFC tracks the motivation of others through representations that not only code for the values and costs of reward to other individuals (Apps et al., 2016), but also incorporates rank information, particularly within one’s own social group, dovetailing with behavioral evidence concerning the influence of social dominance and familiarity on cooperative behavior (Chang et al., 2011, Molenberghs, 2013, Seyfarth and Cheney, 2012).

Our results, however, do provide an apparent contrast with recent work arguing that MPFC representations do not inherently distinguish between self-related and other-related information (i.e., are “agent independent”) (Garvert et al., 2015, Nicolle et al., 2012). Specifically, one study (Nicolle et al., 2012) argued that different MPFC regions may represent value in the context of a temporal discounting task as a function of whether this information is directly relevant to the choice to be executed or not, rather than whether it is self relevant or other relevant. Several factors may account for this discrepancy: one key difference is that in our study, the power of individuals in both the Self and Other hierarchies was arbitrary and needed to be learned through experience. Hence in our study, the emphasis was on learning new self-relevant information rather than on simulation, whereby another’s preferences could be simulated using one’s own preexisting preferences as an anchor (Garvert et al., 2015). Such a simulation would have been futile in our paradigm, because the power of individuals in the Other hierarchy could not be assessed using oneself as a template. One hypothesis, therefore, is that the learning and representation of recently experienced self-related information is subserved by a distinct ventral part of the MPFC, which if appropriate can be leveraged to simulate the preferences and behavior of others, a notion that aligns with the proposal that the simulation of other individuals similar to oneself recruits this region of MPFC (Jenkins et al., 2008, Mitchell et al., 2006).

### Conclusions

Linear hierarchies and related structures (e.g., trees) are pervasive throughout the social domain but are also of more general importance in organizing information in an efficient way to facilitate inductive inferences (Kemp and Tenenbaum, 2008). Our study reveals neural computations by which observations of pairwise “contests” are used to update estimates of individuals’ power within a hierarchy and provides compelling evidence that a Bayesian inference scheme, which has certain parallels with the Trueskill ratings system (Herbrich et al., 2006) used in large-scale multiplayer games, underlies this process, rather than a simpler RL mechanism. At the same time, our results, in ascribing a specific role to the MPFC in the learning of one’s own social hierarchy under tightly controlled experimental conditions, invigorate the debate concerning whether self-relevant information is indeed afforded a unique representational status in the brain.

## Experimental Procedures

See Supplemental Experimental Procedures for a full description of task, computational models, and fMRI analysis procedures.

### Phase 1: Learn, Scanned

In this phase of the experiment, participants acquired knowledge about the Self and Other hierarchies in parallel, with blocks of Self trials alternating with blocks of Other trials and training trial blocks alternating with test trial blocks (see Figure 1 and Supplemental Experimental Procedures for details of trial schedule).

### Phase 2: Categorization, Scanned

In this phase, participants were presented with individual face pictures from the Self and Other hierarchies (excluding the profile pictures depicting themselves in the Self condition or their friends in the Other condition) while performing an incidental categorization judgement (see Figure 6 and Supplemental Experimental Procedures).

## Author Contributions

D.K., A.B., and D.H. designed the study. A.B. and D.K. collected the data. D.K., C.B., and P.D. formulated the computational models. D.K. performed the model fitting. D.K. and A.B. analyzed the data. D.K. and P.D. wrote the paper together with C.B., D.H., and A.B. A.B. and C.B. contributed equally to the study.

## Figures and Tables

**Table 1:** Results of Behavioral Model Fitting

Model	Condition	−LL	BIC	α	β	σ	θ
SMC	Self	136	283	–	0.64 (0.12)	0.11 (0.03)	–
	Other	137	285	–	0.65 (0.15)	0.15 (0.04)	–
RL-ELO	Self	144	299	1.35 (0.12)	1.83 (0.22)	–	–
	Other	146	303	1.31 (0.15)	1.39 (0.08)	–	–
RL-ELO_F_	Self	141	299	1.16 (0.22)	1.22 (0.23)	0.11(0.02)	–
	Other	142	301	1.06 (0.20)	1.18 (0.22)	0.13 (0.03)	–
Value transfer	Self	160	331	0.11 (0.01)	0.13 (0.01)	–	0.22(0.02)
	Other	161	333	0.10 (0.01)	0.14 (0.01)	–	0.19 (0.02)
RW	Self	191	393	0.05 (0.01)	0.37 (0.03)	–	–
	Other	191	393	0.05 (0.01)	0.46 (0.08)	–	–
Base	Self	200	400	–	–	–	–
	Other	200	400	–	–	–	–

−LL, negative log likelihood. Average quantities reported. Models fit individually to participants. Mean (SEM) of best fitting parameters for each individual reported (see Supplemental Experimental Procedures for description of models and parameters).

## Supplemental Information

Document S1. Supplemental Experimental Procedures, Figures S1–S3, and Tables S1–S8

Document S2. Article plus Supplemental Information
