# Supplementary material for: Computations Underlying Social Hierarchy Learning: Distinct Neural Mechanisms for Updating and Representing Self-Relevant Information
Source: Neuron. 2016 Dec 7;92(5):1135–47. doi: 10.1016/j.neuron.2016.10.052 (PMC5158095; doi:10.1016/j.neuron.2016.10.052)
Supplement: Document S1. Supplemental Experimental Procedures, Figures S1–S3, and Tables S1–S8 [file mmc1.pdf]

Neuron, Volume *92*

Supplemental Information

Computations Underlying Social Hierarchy Learning: Distinct Neural Mechanisms for Updating and Representing Self-Relevant Information

Dharshan Kumaran, Andrea Banino, Charles Blundell, Demis Hassabis, and Peter Dayan

SUPPLEMENTAL INFORMATION.

Computations underlying Social Hierarchy Learning: Distinct Neural Mechanisms for Updating and Representing Self-Relevant Information

Dharshan Kumaran[1,3], Andrea Banino[1], Charles Blundell[1], Demis Hassabis[1,2], Peter Dayan[2]

[1]*Google DeepMind, 5 New Street Square, London EC4A 3TW.*

[2]*Gatsby Computational Neuroscience Unit, 25 Howland St, London W1T 4JG, UK.*

[3]*Institute of Cognitive Neuroscience, University College London, 17 Queen Square, WC1N 3AR, UK*

Contains:

3 Supplemental Figures

8 Supplemental Tables

Supplemental Experimental Procedures

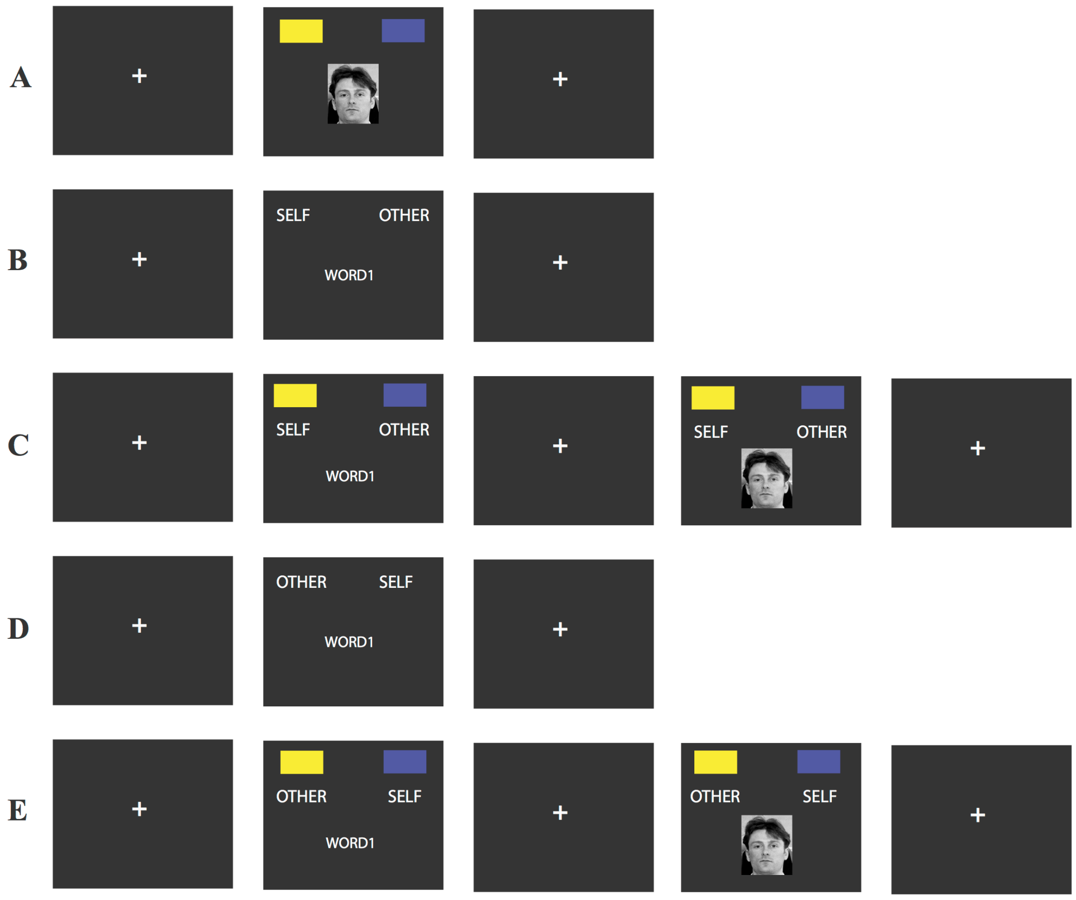

Supplemental Figure 1 (Related to Fig 2). Full Implicit Association Test (IAT) experimental protocol. Example of a sequence of IAT shown. The first step **(A)** introduced the target-concept discrimination. The second step **(B)** introduced the attribute discrimination. In the third step **(C)** congruent trials are presented. In the fourth step **(D)**, participants learned reversal attribute discrimination. In the fifth block **(E)** incongruent trials are presented. The fact of whether participants performed the congruent trials or the incongruent trials first was counterbalanced across participants. See Supplemental Experimental Procedures for details.

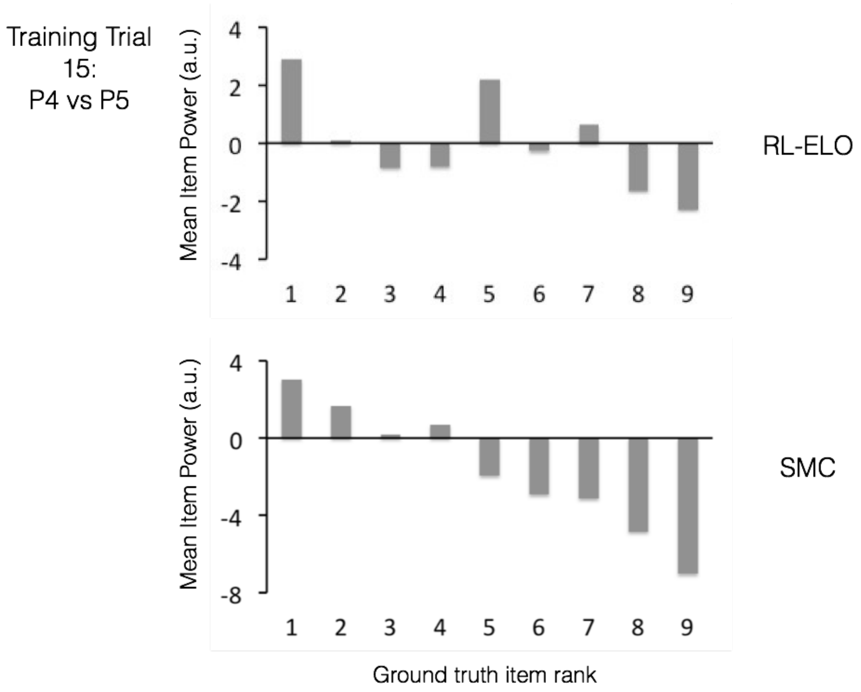

Figure S2A (Related to Fig 4): **Illustrative subject**, mean item powers shown at training trial 15 (i.e. first training trial block). For this case study we describe a high performing subject (average correct overall 92% on training/test trials) who showed a large difference in model fit between the SMC and RL-ELO models: negative log likelihood (NLL) for first ½ of experiment was 53 vs 75 respectively, and for second ½ experiment 23.6 vs 22.6, respectively. We focus on the first block of 16 training trials in the Self condition, where each of the 8 training pairs (e.g. P1 vs P2…P8 vs P9) occurs twice in pseudorandom order (different across subjects). We consider trial 15, which involves items P4 and P5, and the subject responds correctly (i.e. chooses P4). Due to the trial history, there is a transient imbalance in reinforcement associated with the 2 items: item P4 has been the winning item in 1 trial, P5 in 2 trials (i.e. imbalance = -1). The RL-ELO model, with parameters best fit to this subject/condition, is associated with a large NLL due to the imbalance in reinforcement (i.e. NLL for trial 15 =3.1). In contrast, the SMC NLL is 0.1. The mean power of each item at this point (i.e. prior to feedback on trial 15) is shown for the RL-ELO and SMC models (see Fig S2A): the SMC model mean item power already reflect the hierarchy largely correctly, whilst the RL-ELO item powers do not. Note that the SMC model is much less but not entirely insensitive to reinforcement imbalance (see below for systematic analysis): the incorrect ordering of item 3 reflects that this item was the winner in only one previous (P3 vs P4) trial, and was the loser in 2 trials (i.e. P2 vs P3).

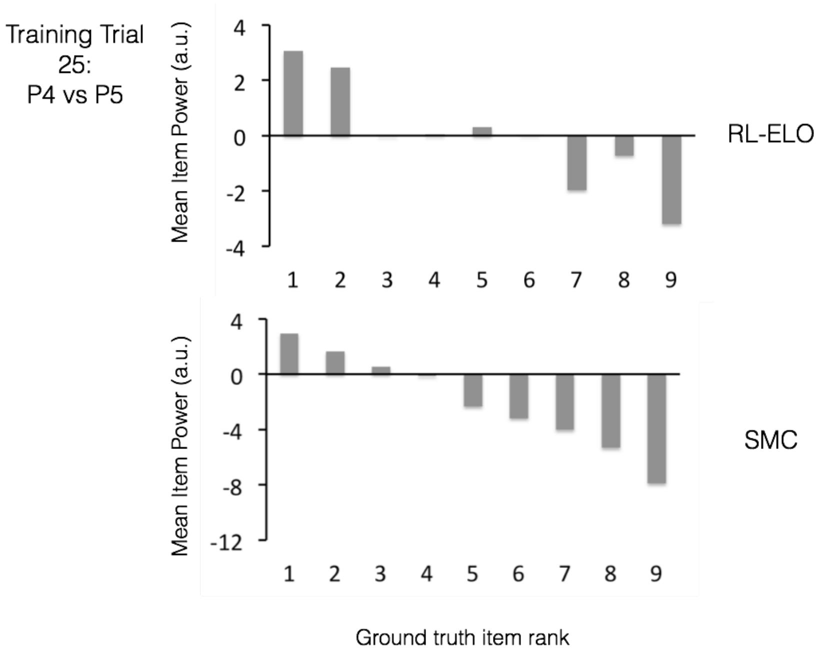

Figure S2B (Related to Fig 4): **Illustrative subject**, mean item powers shown at training trial 25 (i.e. second training trial block). This demonstrates the superior ability of the SMC model to converge on the true rank ordering with less experience. We next carried out a ***systematic analysis*** to assess the differential sensitivity of the SMC and RL-ELO models to transient imbalances in reinforcement history of individual items. To do this, we used the trial orders generated for the actual subjects and focussed on training trials where the reinforcement associated with each item is equated within each block: i.e. training pairs P2 vs P3, P3 vs P4, …., P7 vs P8 (i.e excluding the outer training pairs P1 vs P2 and P8 vs P9 since P1 is always positively reinforced and P9 negatively reinforced). We focussed on the first half of the experiment, and determined the set of parameters that best fit synthetic choice data consisting of all correct responses. We discarded the first set of 7 training trials since the correct response is unknowable at this stage (i.e. even by an "optimal" model). The SMC model NLL was 39.3 (average across Self and Other conditions), whilst the RL-ELO model NLL was 77.2. This large difference in NLL between the SMC and RL-ELO models in fitting this synthetic dataset demonstrates the superior ability of the SMC model to converge on the true rank ordering with less experience (e.g. by trial 25 in the illustrated subject in figure above). We then ran separate linear regression models to quantify the relationship between trial-by-trial reinforcement imbalance (independent variable) and NLL (dependent variable), for the SMC and RL models – where the data was combined across all simulated subjects, with trial type entered as additional independent variable. We ran separate regression analyses for the first and second quarters of the experiment. We found a significant effect of imbalance on NLL for the RL model in the first quarter of the experiment (slope coefficient $b=-0.24$, standard error (se) = 0.0076, $p<0.001$; adj-$R2 = 0.47$) – that was similar for the second quarter ($b=-0.197$, se 0.0072, $p<0.001$ adj-$R2=0.40$). We found a significant but much smaller effect of imbalance in the SMC model in the first quarter ($b=-0.073$, se = 0.0099, $p<0.001$; adj $R2 = 0.046$;) and second quarter ($b=-0.050$, se 0.003, $p<0.001$; adj $R2 = 0.041$). The RL model was associated with a significantly greater effect of imbalance on NLL: ($Z=24.8$, $p<0.0001$; across both quarters). Further, there was a significantly greater effect of imbalance of the SMC model in the first quarter as compared to the second quarter ($Z=2.5$ $p<0.01$). The SMC and RL-ELO models, therefore, have qualitative different updating mechanisms: during a given training trial, the SMC model updates its posterior distribution over item values based on all the available experience – rather than basing its update solely on the current value of the items presented in the trial as in the RL-ELO model. This difference leads the RL-ELO model to be much more sensitive to transient imbalances in the reinforcement history of individual items, as compared to the SMC model – which shows a small sensitivity to reinforcement imbalance that decreased with increasing experience. The difference in updating mechanism results in the SMC model being able to more

rapidly converge on the correct rank ordering: indeed, the difference between model fits (NLL) showed a highly significant correlation with subjects' performance, with high performing subject showing the greatest advantage for the SMC model (pearson's correlation: r = -0.75, -0.79; p<0.0001 for both self and other conditions). Notably, the vast majority of subjects were better fit by the SMC model (22 and 23 out of 28 subjects in Self/Other condition).

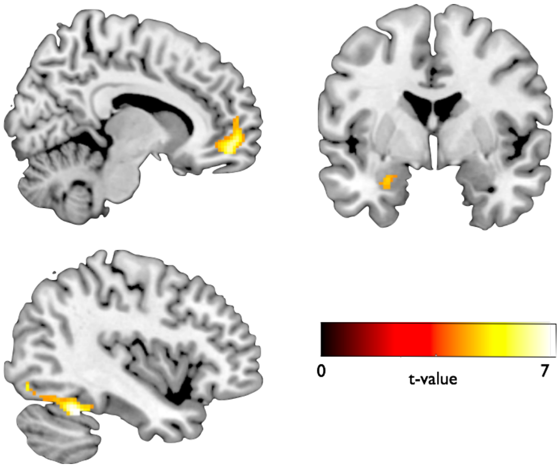

Figure S3A (Related to Fig 5)**:** Learn Phase: Brain regions where activity negatively correlates with SMC-modelled entropy during training trials (Main effect: Self and Other). In an analysis where RT was included in the GLM (correlation with entropy regressors ~ 0.25 across subjects), we found significant negative correlations with the entropy (i.e relating to participants' estimates of the probability of each item winning against the other) in the left amygdala/anterior hippocampus as well as FFA-proximate area and vMPFC. Whole brain analysis: significant correlation between activity in vmPFC (top left), amygdala (top right), FFA-proximate region evident (bottom left) (see Table S5A). Display p<0.005 corrected.

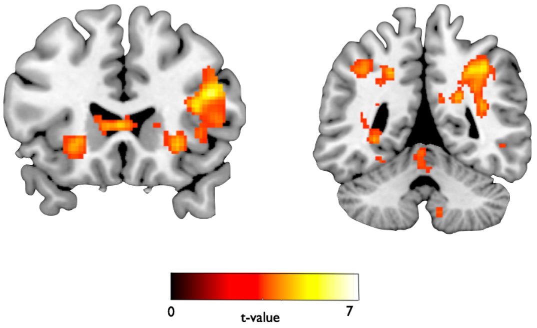

Figure S3B (Related to Fig 5)**:** Learn Phase: Brain regions where activity positively correlates with SMC-modelled entropy during training trials (Main effect: Self and Other). Positive correlations with entropy were found in the dorsolateral prefrontal cortex, insula, medial parietal cortex and intraparietal sulcus. Whole brain analysis: significant correlation between activity in DLPFC (left panel), insula (left panel), intraparietal sulcus (right panel) evident (see Table S5B). Display p<0.005 uncorrected.

Table S1 (Related to Fig 1)**:** Brain areas whose activity significantly correlated with the SMC modelled difference in power during test trials: Main effect across Self and Other conditions

Region	*x*	*y*	*z*	*z-score*	
Amygdala/Anterior HC	-18	-2	-24	5.44	FWE_p p=0.003
Ventromedial PFC	4	60	-10	5.10	FWE_p p=0.013
Posterior Cingulate	0	-32	36	4.45	FWE_c p=0.017
Fusiform	-40	-50	-22	5.78	FWE_p p=0.003
Orbitofrontal	-32	36	-14	4.13	p<0.001 unc

HC = hippocampus, PFC = prefrontal cortex. FWE_p is whole brain FWE corrected at peak-level, FWE_c is whole brain FWE corrected at cluster level (cluster threshold: p=0.001), SVC is small volume corrected (see Supplemental Experimental Procedures for details).

Table S2 (Related to Fig 4)**:** Results of Neural Model Comparison, Relative BIC differences between SMC and RL-ELO models.

**L AMY**	**R AMY**	**L HC**	**R HC**	**MPFC**	**FFA**	**vMPFC**
**41.6***	**38.4**	**46.4***	**43.8**	**32.0****	**17.0***	**3.2**

Numbers reflect relative BIC differences between models (i.e. BIC_RL-ELO – BIC_SMC). Values are summed across participants. Single set of parameters for each model across subjects (i.e. best fit to behavioural data). AMY = amygdala, HC = hippocampus, MPFC and vMFPC are functionally defined regions of medial and ventromedial prefrontal cortex; FFA is FFA-proximate region as referred to in main text, functionally defined (see Supplemental Experimental Procedures). Following previous work (Niv et al., 2015) we also report significance of the difference between the log likelihoods of SMC and RL-ELO, using permutation testing. *significant at p<0.05; ** significant at p<0.01. R HC p=0.068; vmPFC p>0.3.

Table S3A (Related to Fig 4)**:** Brain areas whose activity significantly correlated with the SMC modelled hierarchy update index during training trials: Main effect across Self and Other conditions.

Region	*x*	*y*	*z*	*z-score*	
MPFC	-8	44	10	5.04	FWE_p p=0.022
Hippocampus	-26	-22	-14	3.45	SVC p=0.041
Hippocampus	30	-36	-2	4.51	SVC p = 0.003
Orbitofrontal	30	36	-8	3.61	p<0.001 uncorr
Insula	-28	16	12	3.91	p<0.001 uncorr
Fusiform	-42	-50	-18	4.63	FWEc p=0.014

Table S3B (Related to Fig 4)**:** Brain areas whose activity significantly correlated with the SMC modelled hierarchy update index in Self condition during training trials.

Region	*x*	*y*	*z*	*z-score*	
MPFC	-6	46	12	4.22	SVC p=0.0040
Hippocampus	-24	-12	-16	3.32	SVC p=0.05
Posterior Cingulate	-6	-44	34	4.51	p<0.001 uncorr
Orbitofrontal	38	44	-14	4.21	p<0.001 uncorr

Table S4 (Related to Fig 4)**:** Brain areas whose activity shows significantly greater coupling with functionally defined MPFC seed region during updating of hierarchy knowledge in the Self, as compared to Other, condition.

Region	*x*	*y*	*z*	*z-score*	
Hippocampus	-26	-24	-14	3.25	SVC p=0.042
Amygdala	-18	0	-22	3.04	SVC p=0.040
Orbitofrontal	34	32	-16	3.62	p<0.001 uncorr
Orbitofrontal	-24	38	-8	3.25	p<0.001 uncorr

Table S5A (Related to Fig 5)**:** Brain areas whose activity showed a negative correlation with entropy over item pairs (i.e. probability of item being correct) during training trials: Main effect Self and Other.

Region	*x*	*y*	*z*	*z-score*	
Amygdala/anterior HC	-22	-8	-24	3.51	SVC p=0.032
Amygdala/anterior HC	24	-12	-26	3.42	p= 0.001 uncorr
Fusiform	-44	-52	-24	5.47	FWE_p p<0.003
vmPFC	14	50	-6	3.98	FWE_c p<0.002

Table S5B (Related to Fig 5)**:** Brain areas whose activity showed a positive correlation with entropy over item pairs (i.e. probability of item being correct) during training trials: Main effect Self and Other.

Region	*x*	*y*	*z*	*z-score*	
Dorsolateral PFC	44	22	22	4.65	FWE_c p<0.001
Insula	-26	24	-4	3.92	p<0.001 uncorr
Insula	26	20	-6	4.14	p<0.001 uncorr
Parietal cortex	2	-66	44	5.71	FWE_p p<0.001
Intraparietal sulcus	30	-54	46	5.12	FWE_p p<0.001

Table S6A (Related to Fig 5)**:** Brain areas whose activity significantly correlated with the SMC modelled chosen power during training trials: *main effect of Self and Other conditions*

Region	*x*	*y*	*z*	*z-score*	
vmPFC	-8	52	-12	3.74	SVC p=0.005
Hippocampus	16	-16	20	3.91	p<0.001 uncorr
Hippocampus	-24	-40	18	3.51	p<0.001 uncorr
Amygdala	-18	-4	20	3.13	p<0.001 uncorr
Striatum	14	2	-16	3.71	p<0.001 uncorr
Striatum	-22	10	-6	3.42	p<0.001 uncorr
Orbitofrontal	18	42	-8	3.91	p<0.001 uncorr

Table S6B (Related to Fig 5)**:** Brain areas whose activity significantly correlated with the SMC modelled chosen power during training trials in the *Self* condition

Region	*x*	*y*	*z*	*z-score*	
MPFC	4	44	2	3.01	SVC p=0.037
Striatum	16	8	-12	3.82	p<0.001 uncorr
Striatum	-8	10	-12	4.25	p<0.001 uncorr

Table S6C (Related to Fig 5)**:** Brain areas whose activity significantly correlated with the SMC modelled chosen power during training trials in the *Other* condition

Region	*x*	*y*	*z*	*z-score*	
vmPFC	-12	52	-10	3.86	SVC p=0.016

Table S6D (Related to Fig 5)**:** Brain areas whose activity significantly correlated with the SMC modelled chosen power during training trials: *Self* > *Other* condition

Region	*x*	*y*	*z*	*z-score*	
MPFC	6	42	4	4.23	SVC p=0.001

Table S7 (Related to Fig 6)**:** Brain areas whose activity showed a linear correlation with person rank in Categorization phase: Main effect Self and Other.

Region	*x*	*y*	*z*	*z-score*	
Amygdala	-32	-4	-22	3.72	SVC p=0.025
Hippocampus (anterior)	-32	-8	-20	3.64	SVC p=0.034

Scores	Mean	SD
**Power Explicit Scores:** Can you tell me – use your gut instinct – how powerful each of these individuals feels to you? [a]		
Top Ranked Individuals in the Self Hierarchy	9.07	1.60
Top Ranked Individuals in the Other Hierarchy	8.60	2.90
Bottom Ranked Individuals in the Self Hierarchy	2.28	1.99
Bottom Ranked Individuals in the Self Hierarchy	2.28	2.28
**Social Realism Scores** [a]		
**Top:** How realistic does it feel when you see "X" that he/she is the head of the company/top dog?		
Top Ranked Individuals in the Self Hierarchy	6.55	2.74
Top Ranked Individuals in the Other Hierarchy	6.63	2.43
**Bottom:** How realistic does it feel when you see "X" that he/she is the loser/lowest person in the company?		
Bottom Ranked Individuals in the Self Hierarchy	6.10	2.74
Bottom Ranked Individuals in the Self Hierarchy	6.52	2.43
**Explicit Identification Scores** [a]		
How much did you feel YOU were part of the company in the YOU trials?	5.57	2.34
How much did you feel your FRIEND was part of the company in the HIM/HER trials?	5.33	2.18
How much did you feel YOU were part of the company in the HIM/HER trials?	2.03	2.97
How much did you feel that you FRIEND was part of the company in the YOU trials?	0.83	1.53
**Friend Information** [b]		
How much do you like them?	9.57	0.72
How similar would you say they are to you in terms of overall perspective on life, personality, hobbies?	6.80	1.60

[a] on a scale from 0 (low) to 10 (high)

[b] on a scale from 1 (a little) to 10 (a lot)

Table S8 (Related to Fig 2)**: Results of debriefing session performed at end of experiment.** See Supplemental Experimental Procedures for details.

Supplemental Experimental Procedures.

Participants. Thirty healthy, right-handed individuals who were currently undertaking or had recently completed a university degree, participated in this experiment (age range 19-29; 18 female). Two of these participants performed at chance levels and were therefore excluded from the fMRI analyses. All participants gave informed written consent to participation in accordance with the local research ethics committee.

Stimuli: Faces. Face pictures were obtained from a widely used database (Stirling database: http://pics.stir.ac.uk): pictures are rendered in grayscale and depict male individuals sitting on a chair, with a neutral expression. Images were cropped below the chin line and resized, though hair was retained to preserve the naturalistic properties of the stimuli. Male participants learnt about hierarchies comprised of male individuals, and vice versa for female participants. To represent the participant themselves (i.e. Self condition) and a close friend (i.e. Other condition – see below for procedure for eliciting the friend), two profile pictures were created in Adobe Photoshop CS5. The profile pictures depicted a black silhouette, with the same background, size and colour of the other face pictures. Profile pictures incorporated the pronoun "You", and the pronoun "Him" or "Her" (i.e. for male/female participants, respectively: see Figure 1).

Hierarchies. Self and Other hierarchies were each comprised of 9 items (i.e. P1-P2-P3-P4-P5-P6-P7-P8-P9; where P=person and 1 is the highest ranking person and 9 the lowest ranking - Figure 1). The participant themself, denoted by the "You" profile picture – and their friend, denoted by "Him" or "Her" profile picture – always occupied the middle position in the hierarchy (i.e. P5). This was done to ensure that the rank of the participant and their friend was equated, and allowed us to create a controlled set of transitive inference test trials (see below). Apart from the profile pictures, the allocation of individual pictures to position in the hierarchy was randomized across the group of participants. No significant correlation was found between post-scanning ratings of attractiveness or dominance and rank order (ps > 0.1).

Prior to each scanning session, participants briefly performed a simple 1-back task in which they viewed each individual face three times – a procedure which is known to minimize stimulus novelty effects during scanning based on previous data (e.g. (Johnson et al., 2008)). Examples of faces used are shown in Figure 1.

Tasks and Procedures. Participants were first asked the name of a close friend of theirs that fulfilled two requirements: the friend had to be of the same sex and they had to have known them for more than three months. Participants were asked to imagine that they and their friend had recently joined two different companies. They were informed that the individuals within each company were distinct (i.e. no individual belonged to both companies), and that each company had a distinct coloured logo (i.e. either yellow or blue, assignment to Self or Other condition counterbalanced). As such, the coloured border (e.g. yellow) surrounding a face picture would indicate which company the individual belonged to (i.e. Self or Other). Notably, our experimental design incorporated a close friend in the Other condition – rather than an acquaintance or unfamiliar other individual – in order to render these conditions as similar as possible, thereby isolating the self/other dimension (e.g. see (Mitchell et al., 2006)).

They were informed that there would be two parts to the experiment: in the first phase ("Learn" phase) they would need to learn which individuals have more power within each company. In phase two ("Categorization" phase), they were told that they would need to use knowledge acquired during phase 1 to make judgements about individuals. Participants were told that they would be renumerated based on their performance in the Learn and Categorization phases. Our aim, therefore, was to develop a naturalistic experimental scenario in which subjects would develop knowledge of a social hierarchy that either involved themselves (i.e. Self condition) or a close friend (i.e. Other condition).

Phase 1 (Learn) In this phase of the experiment participants acquired knowledge about the Self and Other hierarchies in parallel.

Training trials (Figure 1). During a training trial, participants viewed adjacent individuals in each of the Self and Other hierarchies displayed on either side of the screen (i.e. 8 training pairs in Self and Other

conditions: i.e. P1 vs P2, P2 vs P3, P3 vs P4, P4 vs P5, P5 vs P6, P6 vs P7, P8 vs P9). The left-right position of an item on the screen was randomized across trials. They had 3 seconds in which to choose, via button press (i.e. left or right, index or middle finger of right hand respectively), the item which had "more power". After 3 seconds, a feedback screen appeared (2 second duration): this consisted of white circle below the chosen stimulus together with either "+20 points" or "-20 points", for a correct or incorrect response (in green/red color, respectively). A fixation cross of 1.5 seconds duration preceded the onset of the next trial. The renumeration received by participants for this phase of the experiment was determined directly from the number of points won.

Test trials (Figure 1B). During test trials, participants viewed pairs of non-adjacent individuals in the hierarchy (i.e. 8 inference pairs: P2 vs. P4, P2 vs. P5, P3 vs. P5, P3 vs. P6, P4 vs. P6, P4 vs. P7, P5 vs. P7, P5 vs. P8). Note that 4 of the inference pairs included the participant or their friend (e.g. P2 vs P5), and 4 did not (e.g. P3 vs P6). As in training trials, participants had 3 seconds in which to choose, via button press (i.e. left or right), the person which they thought had more power in either the hierarchy of which they were a part (i.e. Self condition), or that incorporated their friend (i.e. Other condition). Importantly, however, no feedback was presented during test trials, though participants were instructed that their choices would still count towards their final payout. Instead, after 3 seconds, a screen appeared which required participants to rate (on a scale of 1 to 3) their confidence in their decision: participants were carefully instructed to enter a "1" response if they were guessing entirely, a "2" response if they were "had some idea but were not sure" about their choice, and to reserve a "3" response until they were "more than 90% certain" that their choice was the correct one. Participants were told that though their confidence responses would not count towards their final payout, they should still answer as accurately as possible.

Schedule of trial presentation.
Blocks of Self trials alternated with blocks of Other trials, with block order (i.e. whether Self or Other condition appeared as the first block) counterbalanced across subjects. Each block was comprised of a 16 trial miniblock made up of each of the 8 training trial types repeated twice, followed by a 8 trial miniblock of each of the test trial types. The order of training and test trials was pseudorandomized and varied across blocks. The start of each miniblock was preceded with the relevant instruction which was presented for 2 seconds (i.e. "You Training trials", "Him" or "Her" Test trials). At the end of each training and test block, participants received cumulative feedback indicating their performance during that block (2 seconds).

In total, there were 12 blocks for each of the Self and Other conditions – i.e. 192 training trials, and 92 test trials, in each condition. Phase one consisted of three sessions of approximately 20 minutes each, separated by a 1 minute break during which time participants remained inside the MRI scanner.

Phase 2: Categorization (scanned)
In this phase, participants were presented with individual face pictures from the Self and Other hierarchies (excluding the profile picture depicting themself in the Self condition, or the friend in the Other condition). Each picture was repeated 4 times, duration 2.3 seconds with 0.8 seconds fixation cross between stimuli. Participants had to make a categorization judgment i.e. to determine whether the person belonged to the company with the yellow or blue logo. Presentation order was pseudorandom. 64 trials were divided over 2 experimental sessions lasting approximately minutes each. Participants had a 1 minute break between sessions during which time they remained inside the scanner.

Implicit Association Test (IAT: not scanned).
Following the end of the scanning part of the experiment, participants completed a version of the IAT test tailored to address our question of interest (Greenwald et al., 1998; Greenwald & Farnham, 2000) to probe the effectiveness of our experimental manipulation: i.e. the extent to which subjects incorporated themselves and their friend into the hierarchies.

We describe the task in detail below. Briefly, the rationale behind this paradigm is as follows and is broadly analogous to the Stroop effect (e.g. (Cohen et al., 1990)): consider that participants have actually incorporated themselves into the Self hierarchy, and that this company has a yellow logo (note color counterbalanced across participants). When participants view face pictures, they should be faster to

categorize in the **congruent** condition: when the yellow logo is displayed on the *same* side as the word "self" and when the blue logo is displayed on the *same* side as the word "other". In contrast, RTs should be slower in the **incongruent** condition: where the yellow logo is displayed above the word "other". Note that the words used in the IAT test – "self" and "other" were not used in other parts of the experiment (e.g. "You" trials and "Him" or "Her" trials in the Learning phase).

IAT Stimuli: The stimuli used during the IAT test consisted of the face pictures used in the main experiment, and also word stimuli**.**
Word stimuli**:** A set of 8 English pronouns were used: 4 of these pronouns – me, mine, myself, my – are known to be associated with the self concept and 4 pronouns – theirs, they, them, themselves – are known to be associated with the concept of another person. These pronouns were selected based on a pilot study to equate behavioural performance across both the Self and the Other conditions.

Trial Blocks (Figure below) As in the typical IAT test, the task was divided into several distinct blocks – 5 in our case.

The first block (Figure S1A) introduced the so-called "target-concept discrimination"; in this block participants viewed two rectangles in the top of the screen – one on the left, and one on the right – representing the respective colours of the logo of their company and their friend's company. The left-right position of the Self-colour and Other-colour logos was counterbalanced between participants. Participants viewed pictures of individuals from the Self and Other companies (with the exception of the profile pictures), and had 3 seconds to respond by button press (either Q or P, corresponding to left or right response) whether the individual was a member of the blue or the yellow company. A fixation cross, presented for 1 second, preceded the presentation of the next trial. There were 64 trials in this block: each picture presented 4 times. Order of stimulus presentation was pseudo-randomised across participants, in all blocks.

The starting position of the Self-colour (e.g. yellow) and Other-colour (e.g. blue) logos were counterbalanced between participants. This, in combination with the counterbalanced allocation of color (yellow/blue) to Self/Other hierarcy, allowed us to counterbalance whether participants performed the congruent trials – i.e. the word "self" and "other" positioned on the *same* side of the Self company colour logo as experienced during the first hierarchy learning phase – or the incongruent trials first.

The second block introduced the "attribute discrimination" (Figure S1B). Participants were presented with the word "self" on the left and the word "other" on the right side of the screen. In this section, participants were presented with one of the 8 word stimuli – pronouns (see above) – and had 3 seconds to respond as to whether they were related to the concept of the self or another person. A fixation cross, presented for 1 second, preceded the presentation of the next trial. 16 trials in this block, each pronoun repeated twice.

In the third block (Figure S1C) both the target concepts (i.e. colored logos) and the attributes (i.e. "self" or "other") were presented on the screen. The words ("self" and "other") and logos (yellow/blue) were presented on the same sides as in the preceding blocks (i.e. blocks 1 and 2). Participants viewed alternating pronouns and face pictures, and were required to respond according to whether they related to self/other concept, or yellow/blue logos, respectively. 96 trials in this block: 48 pronouns (each pronoun repeated 3 times) and 48 pictures (each picture repeated 3 times).

In the fourth block (Figure S1D) participants performed the same task as in block 2, but the position of the words "self" and "other" were reversed. 16 trials in this block, each pronoun repeated twice.

Finally, in the fifth block (Figure S1D) participants performed the same task as in block 3 with the exception that the position of the words "self" and "other" were as in block 4, and therefore swapped in side compared to block 3. 96 trials in this block: 48 pronouns (each pronoun repeated 3 times) and 48 pictures (each picture repeated 3 times).

Post-Experimental Debriefing (after completion of IAT test). Participants were carefully debriefing following the end of the IAT test. Included in this assessment was a test assessing participants' declarative knowledge of the hierarchy: pictures of the two sets of people were presented to participants, and they were asked to rank them in terms of their order in the hierarchy, with their performance timed.

Debriefing scores: Participants were also asked to evaluate how "real" the social rank dimension seemed – and to rate how much they felt part of the Self hierarchy, and how much they felt their friend was part of the Other hierarchy (see Table S8 for full details: appended below).
Then participants responded to a question that assessed their feelings about the level of power of the first-ranked and last-ranked individuals in both hierarchies, the Power Explicit Scores: "Can you tell me – use your gut instinct – how powerful each of these individuals feels to you on a scale from 0-low to 10-high". After that, they were also asked to evaluate the level of realism of the social rank, the Social Realism Scores: 1) "How realistic does it feel when you see this person – the two individuals at the top of the hierarchies presented – that he/she is the head of the company/top dog on scale from 0 to 10 (10=realistic, 0 = unrealistic) and 2) "How realistic does it feel when you see this person – the two individuals at the bottom of the hierarchies presented – that he/she is the loser/lowest person on a scale from 0 to 10 (10=realistic, 0 = unrealistic)". Then, we obtained four explicit measures on how much participants identified themselves and their friend with the respective hierarchies, the Explicit Identification Scores: 1) "How much on a scale from 0-not at all to 10-a lot did you feel you were part of the company in the YOU trials", 2) "How much on a scale from 0-not at all to 10-a lot did you feel your friend was part of the company in the HIM/HER trials", 3) "How much on a scale from 0-not at all to 10-a lot did you feel YOU were part of the company in the HIM/HER trials", and 4) "How much on a scale from 0-not at all to 10-a lot, did you feel your FRIEND was part of the company in the YOU trials". Next, we asked some general questions about their friend: "How much do you like them? (on a scale 1 to 10, 1 is a little, 10 is a lot), and "How similar would you say they are to you in terms of overall perspective on life, personality, hobbies ? (from 1 to 10)". Finally, participants rated all the face stimuli according to three traits: dominance, trustworthiness, attractiveness – i.e. How "trait" is this person? (1=not at all, 9=extremely), use your gut feelings.

"In phase 2, when you saw a picture of an individual who was more highly ranked in the company, how "real" did it seem that they were more highly ranked or had more power in the company etc? Please rate this on a scale of 1-10 (10 = a lot, 1 = not at all)- as an example, if when you saw the most highly ranked guy you thought to yourself that's the topdog/head-guy, then your answer is likely to be nearer the 10 end of the scale"

"How much on a scale from 0-not at all to 10-a lot did you feel you were part of the hierarchy in the YOU trials?

"How much on a scale from 0-not at all to 10-a lot did you feel your friend was part of the company in the hierarchy in the HIM/HER trials?"

Behavioral analyses. Analyses were conducted using SPSS software (www.spss.com), Matlab 7.0 (www.mathworks.com/products/matlab).
Implicit Association Test. Before analysing the data from the IAT, we applied the reduction-data procedure describe by (Greenwald et al., 2003). Specifically we followed these steps: 1) subjects for whom more than 10% of trials had a latency less than 300ms were eliminated; 2) subjects for whom more than 20% of trials were error trials were eliminated; 3) the means of only the correct latencies for block 3 and block 5 were computed; 4) the pooled standard deviation for all trials in block 3 and block 5 was computed; 5) Each error latency in block 3 and 5 was replaced by the block mean computed at point 3 plus 600ms; 6) The difference between the mean latencies of block 3 and block 5 was computed; 7) The difference computed in step 6 was divided by the pooled standard deviation calculated in step 4 to obtain the correct IAT measure – the D measure – used in all the analyses. Following this procedure, 6 participants were excluded from the analysis.

Computational Models.

Sequential Monte-Carlo (SMC) model. SMC models (Doucet et al., 2000)are a type of state-space inference model that aims to infer the underlying state of an evolving dynamic system: in this case the level of power of a set of individuals. These hidden states (i.e. power levels) diffuse across training trials according to a Gaussian random-walk model (a non-zero variance for which models forgetting). Dominance decisions are assumed to be generated according to an observation process – here a random choice based on a sigmoid of the discrepancy in power between the individuals. The SMC method, which is a form of Bayesian filter, captures the way that a participant can use information about the hierarchy that is acquired over the course of the experiment to make inferences about these powers. We consider an on-line filter, according to which estimates of the powers are based on past data up to the current trial.

SMC models – also known as particle filters – relax the conventional assumption for linear, Gaussian, Kalman filters, that the probability density function (pdf) of the inferred variables (i.e. power) is a normal distribution, thereby extending their flexibility in modelling complex multi-modal distributions in a range of domains (e.g (Doucet et al., 2000)). In the SMC model, each particle (here, *N*=10,000) contains one set of values for the hidden state variable (i.e. power). Hence a particle can be viewed as representing a hypothesis about the rank ordering of items within the hierarchy: the population of particles, therefore, constitutes a multimodal (i.e. 9-dimensional, given 9 items in each hierarchy) pdf of rank. Particles are initialized with equal weight (see below), with their weights being updated on each training trial depending on the likelihood of the trial outcome given the hypothesis concerning rank ordering they represent. A particle resampling step ensures that the density of particles is highest in regions of the (9-dimensional) space that are likely given the history of observed data (i.e. have highest weights), by tending to replace conditionally unlikely particles (i.e. with low weights) with new, more appropriate, ones.

Prior model: the state variable (i.e. $\vec{x}_0$, denoting power) is initialized a normal distribution with fixed initial variance σ_0^2 (=10), and zero mean (Eq 1). The state process is described as a Gaussian random walk with evolution variance σ^2 (free parameter)(Eqn 2) – this instantiates a form of imperfect memory (i.e. forgetting), in order to account for participants needing ~ 200 trials to achieve proficiency on the task. The observation model (Eqn 3) is a sigmoid function of the difference between the distributions of the two items presented in a given training trial *t*: parameterized by beta i.e. the item with current highest expected value (a_t) and that with the lower value (b_t). y_t = 1 denotes the situation when the highest valued item is the correct response.

$$\vec{x}_0 \sim \mathcal{N}\left(\vec{0}, \sigma_0^2 \mathbf{I}\right) \qquad (1)$$

$$\vec{x}_t | \vec{x}_{t-1} \sim \mathcal{N}\left(\vec{x}_{t-1}, \sigma^2 \mathbf{I}\right) \qquad (2)$$

$$g(y_t = 1 | a_t, b_t, x_t) = \frac{1}{\left(1 + e^{-\beta * (x_{a_t t} - x_{b_t t})}\right)} \qquad (3)$$

Particle filter: Let i index particles, of which there are N $(= 10{,}000)$. Particles are initialized as samples from a normal distribution, with zero mean and variance σ_0^2 (Eqn 4), each with an equal weight $(\widetilde{w}^{(i)})$, which are then normalized (Eqn 5). State process equation for the particles – note since no feedback was provided during test trials, we assumed that this process only occurred during training trials (Eqn 6). The unnormalized weights $(w_{t+1}^{(i)})$ of the particles are updated using the observation model and the normalized weights from the previous timestep (Eqn 7).

$$\vec{x}_0{}^{(i)} \sim \mathcal{N}(0, \sigma_0^2 \mathbf{I}) \qquad (4)$$

$$\widetilde{w}^{(i)}{}_0 = \frac{1}{N} \qquad (5)$$

$$\vec{x}_t^{(i)} \sim \mathcal{N}\left(\vec{x}_{t-1}^{(i)}, \sigma^2 \mathbf{I}\right) \quad (6)$$

$$w_{t+1}^{(i)} = g\left(y_t \middle| a_t, b_t, \vec{x}_t^{(i)}\right) \widetilde{w}_t^{(i)} \quad (7)$$

N_{eff} determines the threshold for resampling (Eqn 8).

$$N_{eff} = \frac{1}{\sum_{i=1}^{N} \widetilde{w}_t^{(i)} \cdot \widetilde{w}_t^{(i)}} \quad < 0.25N \quad (8)$$

If $N_{eff} \quad < 0.25N$

then resample (with replacement):

$$a_t^{(i)} \sim \text{Categorical}\left(\left\{\frac{w_t^{(i)}}{\sum_{i=1}^{N} w_t^{(i)}}\right\}_{i=1}^{N}\right) \quad (9)$$

$$x_t^{(i)} = x_t^{(a_t^{(i)})} \quad (10)$$

$$\widetilde{w}_t^{(i)} = \frac{1}{N} \quad (11)$$

$$\textit{Otherwise}\ \widetilde{w}_t^{(i)} = w_t^{(i)} \quad (12)$$

The estimate of marginal likelihood is:

$$\hat{p}(y_{1:T}) \quad = \prod_{t=1}^{T} \frac{1}{N} \sum_{i=1}^{N} w_t^{(i)} \quad (13)$$

Note: an internal variable of the model used to interrogate the fMRI data is the *SMC model choice entropy*: $H(y_t) = -p \log p - (1-p)\log(1-p)$; where $p = P(y_t = 1)$ the probability of selecting the correct option, given the weighted set of particles at the current trial.

RL model (RL-ELO).
In the RL-ELO model, rather than updating based on the difference between trial outcome and current value (as in Rescorla Wagner), the value update is a function of the difference in current values between the two items (i.e. indexed by their positions, left and right, which was randomized on every trial): $V_{L,t}$ & $V_{R,t}$
We term this model RL-ELO because of its relationship to algorithms used to update rankings (termed "ELO") in multiagent scenarios (e.g. chess) – though it should be noted that it can be shown to be a version of a policy gradient algorithm (Williams, 1992).

Initialize all ranks at V_0=0
free parameters: α = learning rate; β=temperature
Training trial at time *t* with items on left and right sides of screen
Probability of choosing left item, and right item:

$$p_{L,t} = \frac{1}{\left(1+e^{-\beta*(V_{L,t} - V_{R,t})}\right)} \quad (1)$$

$$p_{R,t} = 1 - p_{L,t} \quad (2)$$

if L item is correct choice:

$$p_{win,t} = p_{L,t} \quad (3)$$

$$I_{L,t} = 1, I_{R,t} = -1 \quad (4)$$

if R item is correct choice:

$$p_{win,t} = p_{R,t} \quad (5)$$

$$I_{L,t} = -1, I_{R,t} = 1 \quad (6)$$

Update values of both items:

$$V_{L,t+1} = \alpha * I_{L,t} * (1 - p_{win,t}) + V_{L,t} \quad (7)$$

$$V_{R,t+1} = \alpha * I_{R,t} * (1 - p_{win,t}) + V_{R,t} \quad (8)$$

Note that we also examined a variant of the model, termed RL-ELO$_F$, which incorporated an extra free parameter (σ) that controlled the amount of Gaussian noise (i.e. mean = 0 , SD = σ) that was added to the values at each timestep. This parameter instantiated a form of forgetting, akin to the role of the evolution variance parameter in the SMC model.

Value Transfer model (von Fersen et al., 1991)
This model incorporates the standard update term from Rescorla Wagner, but also includes an indirect component: the incorrect item in a training trial has its value updated with a proportion (i.e. theta %) of the correct item.
Trial outcomes are +1 for correct choice, and -1 for incorrect choice.
3 free parameters: α = learning rate; β=temperature ; θ = transfer factor
Training trial at time *t* with items on left and right sides of screen
Probability of choosing left item, and right item:

$$p_{L,t} = \frac{1}{\left(1 + e^{-\beta * (V_{L,t} - V_{R,t})}\right)} \quad (1)$$

$$p_{R,t} = 1 - p_{L,t} \quad (2)$$

if L item is correct choice, trial outcome & indicator variable (for indirect update):

$$O_{L,t} = 1, O_{R,t} = -1 \quad (3)$$

$$I_{L,t} = 0, I_{R,t} = 1 \quad (4)$$

If R item is correct choice, trial outcome & indicator variable:

$$O_{L,t} = -1, O_{R,t} = 1 \quad (5)$$

$$I_{L,t} = 1, I_{R,t} = 0 \quad (6)$$

Calculate direct component of update:

$$\delta V\_direct_{L,t} = (O_{L,t} - V_{L,t}) * \alpha \quad (7)$$

$$\delta V\_direct_{R,t} = (O_{R,t} - V_{R,t}) * \alpha \quad (8)$$

Calculate indirect component of update:

$$\delta V\_indirect_{L,t} = V_{R,t} * I_{L,t} * \theta \quad (9)$$

$$\delta V\_indirect_{R,t} = V_{L,t} * I_{R,t} * \theta \quad (10)$$

Total update:

$$V_{L,t+1} = \delta V\_direct_{L,t} + \delta V\_indirect_{L,t} \quad (11)$$

$$V_{R,t+1} = \delta V\_direct_{R,t} + \delta V\_indirect_{R,t} \quad (12)$$

Rescorla Wagner. As for Value transfer model, where theta parameter is set to zero.

Computational model fitting. We quantified the fit of all models and a base model (random choice) – to participant's choice behavior during training and test trials. We used a maximum likelihood estimation procedure and optimized a separate set of parameters for each participant (Wimmer et al., 2012). We report the negative log likelihood of each model, and the corresponding BIC measure which penalizes more complex models.

FMRI Design & Analysis.
fMRI design. The temporal pattern of stimulus presentation was designed to maximise statistical efficiency whilst preserving psychological validity, in line with established procedure (Frackowiak et al., 2004; Friston et al., 1998; Josephs and Henson, 1999). Importantly, the haemodynamic response to events that occur a few seconds apart is explicitly modelled (via a haemodynamic response function), and therefore can be estimated separately for each event type by implementing the general linear model as is standard when using statistical parametric mapping software (SPM8) (www.fil.ion.ucl.ac.uk/SPM) (also see below) (Friston et al., 1998).
Functional imaging acquisition parameters. T2 weighted gradient-echo planar images (EPI) with BOLD (blood oxygen level dependent) contrast were acquired on a 3.0 tesla Siemens Allegra MRI scanner using a specialized sequence to acquire whole brain coverage, whilst minimizing signal dropout in the medial temporal lobe and ventromedial prefrontal cortex(Weiskopf et al., 2006). We used the following scanning parameters to achieve whole brain coverage: 48 oblique axial slices angled at 30^0 in the anterior-posterior axis, TR 2.88 seconds, TE 30ms, 2mm thickness (1mm gap), in-plane resolution 3x3 mm, z-shim -0.4mT/m*ms, negative phase encoding direction. High-resolution (1x1x1mm) T1-weighted structural MRI scan were also acquired for each participant after functional scanning. These were coregistered to the functional EPIs, and averaged across participants to aid localization.

fMRI data preprocessing. Images were analyzed in a standard manner using the statistical parametric mapping software SPM8 (www.fil.ion.ucl.ac.uk/SPM). After the first six "dummy volumes" were discarded to permit T1 relaxation, EPI images were spatially realigned and unwarped using fieldmaps,

followed by spatial normalization to a standard EPI template. Normalized images were smoothed using a gaussian kernel with full width at half maximum of 8mm.

Phase 1 (Learn) fMRI data analysis. Following preprocessing, the event-related fMRI data were analyzed in SPM8 using the general linear model (GLM) following established procedures (Frackowiak et al., 2004; Friston et al., 1998). We targeted our analyses to detect brain regions whose activation pattern during test and training trials significantly correlated with participant-specific trial-by-trial parametric regressors: obtained from key hidden variables of the relevant computational models (SMC, RL-ELO). Note that following previous work (Daw et al., 2006; Wimmer et al., 2012) suggesting that using individually optimized parameters to analyse the fMRI data tends to lead to noisy fitting, we used a single set of parameters for the SMC, and the RL-ELO model (i.e. best fit parameters across the group).

Specification of first-level design matrix.
Test trials. As a first step, the 5 second period during which item pair and confidence rating were displayed during test trials was modeled as a boxcar function and convolved with the canonical haemodynamic response function (HRF) to create regressors of interest. All test trial types (i.e. 8 pairs: see above) were modeled within these regressors, with one regressor for the Self condition and one for the Other condition. These participant-specific parametric regressors were also convolved with the HRF, leading to the height of the HRF for a given event being modulated accordingly. Thus, these parametric regressors model BOLD signal changes that covary with a specific internal variable of the SMC model on a given trial (i.e. over and above non-specific effects captured by earlier parametric regressors such as RT). Further, participant-specific movement parameters were included as regressors of no interest. A high pass filter with a cutoff of 180 seconds was employed. Temporal autocorrelation was modelled using an AR(1) process.

In the first model, the parametric modulator was the difference between SMC model estimates of item power: this was a trial-by-trial variable obtained from the SMC model, by taking the expectation over the difference between estimated power of the items presented.

Training trials. As a first step, the 5 second period during which item pair and outcome was displayed during training trials was modeled as a boxcar function and convolved with the canonical haemodynamic response function (HRF) to create regressors of interest. All training trial types (i.e. 8 pairs: P1 vs P2, P2 vs P3....) were modeled within these regressors, with one regressor for the Self condition and one for the Other condition.

In this model, the parametric regressor included was termed the **hierarchy update index**: trial-by-trial estimates derived from the SMC model, to capture the change in hierarchy knowledge consequent on feedback on a given training trial.

For each pair of items (e.g. 1 & 2) we computed a trial-by-trial KL divergence measure, with respect to the probability of one item winning against the other (i.e. before and after feedback).

$$D_{KL}(p \mid q) = \sum_{i=1}^{2} p(i) \log \frac{p(i)}{q(i)}$$

where p(1) is the probability of item_1 winning against item_2 following updating after feedback, and q(1) is that before feedback; where the probability of winning was calculated as elsewhere using a sigmoid function parameterized by beta. Note p(2) = 1 – p(1), and similarly q(2) = 1 – q(1). Then the KL divergence was summed across all 36 pairs. This analysis, therefore, for set up to identify regions where neural activity correlated positively with a change in participants' hierarchy knowledge. Note that since no feedback was provided, there was no change in hierarchy knowledge during test trials.We also set up a model to identify neural regions that showed a correlation with the chosen power – computed from the SMC model as the expectation over distribution of the item power – during training trials. SMC estimated chosen power was the parametric modulator entered into the design matrix.

Neural model comparison: Following previous work (Ashby and Waldschmidt, 2008; Niv et al., 2015; Wilson and Niv, 2015), we ran separate GLMs for the SMC and RL-ELO models using using parametric regressors relating to the hierarchy update index (i.e. using a single set of parameters for each model best fit to the group behavioral data). The negative log likelihood (NLL) of each model, calculated separately for each region of interest (e.g. amygdala) was:

$$NLL = -n * (ln\,(\sqrt{2\pi\sigma^2} + 0.5)$$

where σ^2 is the variance of the residuals from the GLM, and n is the number of scans. We then calculated the relative difference in BIC between models: for completeness, and also following previous work (Niv et al., 2015) we tested whether the difference between model NLL was significantly different from zero using permutation testing.

Phase 2 (Categorization). fMRI data analysis. Following preprocessing, the event-related fMRI data were analyzed in SPM8 using the general linear model (GLM) following established procedures (Frackowiak et al., 2004; Friston et al., 1998). We set up a parametric model to detect brain regions whose activation pattern exhibited a significant linear correlation with the rank of person in the Self and Other hierarchies.

Specification of first-level design matrix. The 2 second trial period during which the face image was displayed on the screen and participants made their response, was modeled as a boxcar function and convolved with the canonical haemodynamic response function (HRF) to create regressors of interest.

Parametric model. Separate regressors were included for Self and Other conditions. **Rank**, from 1 to 9, was included as a parametric modulator in the GLM: linear and quadratic components were modeled. Note that rank 5 was not included in the model since participants did not view the profile pictures of themselves or their friends during this phase. Thus, these regressors model BOLD signal changes that covary with specific indices on a given trial (e.g. the rank of a person).

"Separate rank" model: This model was used for two purposes: i) firstly, it was used as an illustrative model to graphically represent the linear relationship between neural activity in a given brain region (e.g. amygdala) and person rank (see Figure 6; also see(Winston et al., 2002) & Kumaran et al 2012 for a similar useage). In this case, the parametric model specified above was used for statistical inference- i.e. to ask which brain regions show a significant linear correlation between the amplitude of neural activity and person rank. ii) this model was also used to ask whether activity in the MPFC ROI, defined based on orthogonal selection contrast (i.e. from during a separate scanning phase) distinguished rank extremes

Model Estimation. Model estimation proceeded in two stages. In the first stage, condition-specific experimental effects (parameter estimates, or regression coefficients, pertaining to the height of the canonical HRF) were obtained via the GLM in a voxel-wise manner for each participant. In the second (random-effects) stage, participant-specific linear contrasts of these parameter estimates, collapsed across the 2 sessions, were entered into a series of one-sample t tests [as is standard when using SPM(Frackowiak et al., 2004)], each constituting a group-level statistical parametric map.

Statistical inference.
Voxel-based analyses. ***Voxel-based analyses***. We report results in a priori regions of interest - the hippocampus, amygdala, vmPFC and MPFC - where activations are significant at $p<0.001$ uncorrected for multiple comparisons, and survive small volume correction (SVC) for multiple comparisons (at $p<0.05$ corrected) using SPM8. For the SVC procedure we used anatomical masks, for the bilateral hippocampus and amygdala. For the vmPFC, and MPFC we used 6mm spheres centred on coordinates (MNI x, y, z -4,

52, -14 and 6, 48, 4) derived from previous related studies: (Kumaran et al., 2012; Kumaran and Maguire, 2005).

Activations in other brain regions were only considered significant if they were significant at a level of $p<0.001$ uncorrected, and additionally survived whole brain FWE correction at the at the peak level or cluster level ($p<0.05$ corrected, with cluster threshold defined at $p<0.001$), in line with established procedures(Frackowiak et al., 2004).Reported voxels conform to MNI (Montreal Neurological Institute) coordinate space. Right side of the brain is displayed on the right side.

Region of Interest (ROI) analyses. We performed anatomically defined ROI analysis (using the MarsBar SPM toolbox: http://marsbar.sourceforge.net/) in the amygdala, hippocampus, and vMPFC (defined in the same way as for the SVC procedure outlined above). Further, we defined a functional MPFC ROI (Figure 7) based on the results of an analysis during the Learn phase (i.e. significantly greater correlation with chosen power during Self (cf Other) condition) that was subsequently used in a different scanning phase (i.e. the Categorization phase). ROI defined at a level of $p<0.001$ uncorrected. We also defined a ROI proximate to the fusiform face area based on a constrast defined on the categorization data (i.e. specifying the onset of face events vs implicit baseline, at $p<0.001$ uncorrected).

It is important to note that these analyses treat data from a ROI as if it was from a single voxel and hence no correction for multiple comparisons is necessary. Results, therefore, were considered statistically significant where they pass a threshold of $p<0.05$.

Selection contrast is unbiased with respect to contrasts of interest. ROI analyses are widely held to be a powerful tool for affording additional insights, above and beyond that provided by univariate fMRI analysis(Kriegeskorte et al., 2009). Recent work has highlighted potential shortcomings of previous work, and established a theoretically principled approach for carrying out an ROI analysis. Importantly, our analyses fulfil the criteria outlined by (Kriegeskorte et al., 2009): the definition of these ROI is *unbiased* – either based on a different portion of the data (i.e. training trials vs test trials), or on a different scanning phase (i.e. Learn phase vs Categorization phase) – and therefore statistically independent from the effects we examine.

Psychophysiological Interaction (PPI) Analysis
A PPI analysis is employed to identify the presence of functional coupling between different brain regions, by showing that activity in a distant region can be accounted for by an interaction between the influence of a source region and an experimental parameter (Friston et al., 1997). We followed established procedures (O'Reilly et al., 2012) to perform the PPI analysis by creating a GLM that included regressors capturing i) the physiological effect (here, the time series of activity in the MPFC seed region) ii) the psychological contrast of interest: here, hierarchy update: Self > Other (i.e. designed to identify regions showing a greater correlation with hierarchy update index in Self, as compared to Other, condition (see Main Text). iii) psychophysiological interaction term (i.e. physiological effect x psychological contrast of interest)

Specifically: we used SPM8 to first extract the time series (i.e. physiological effect) for the peak voxel in the MPFC (i.e., 6 mm sphere centered on peak coordinate in the group analysis x, y, z, = -6, 46, 12), identified in the correlation of training trial related activity in the Learn phase with the hierarchy update index, collapsed across both Self and Other conditions (see Figure 4B and Table S3A). Next, we calculated the psychological contrast of interest (i.e. hierarchy update: Self > Other). Finally, we calculated the product of the first two signals. The physiological effect and psychophysiological interaction were entered as regressors within the GLM: in addition, we entered a regressor capturing the psychological contrast of interest (i.e. hierarchy update: Self > Other) *without* the interaction with physiological effect. The effect of the psychophysiological interaction term was assessed for each participant and entered into a second level group-level analysis.
The configuration of the PPI GLM, therefore, allows us to ask in which brain regions the magnitude of functional coupling of neural activity with the MPFC seed region shows a significantly greater correlation with the amount by which hierarchy knowledge changes in the Self, as compared to the Other condition – *above and beyond that* explained by differences in the correlation between the hierarchy update index in the Self and Other conditions.

Supplemental References

Ashby, F.G., Waldschmidt, J.G., 2008. Fitting computational models to fMRI. Behav. Res. Methods 40, 713–21.

Cohen, J.D., Dunbar, K., McClelland, J.L., 1990. On the control of automatic processes: a parallel distributed processing account of the Stroop effect. Psychol. Rev. 97, 332–61.

Daw, N.D., O'Doherty, J.P., Dayan, P., Dolan, R.J., Seymour, B., 2006. Cortical substrates for exploratory decisions in humans. Nature 441, 876–9.

Doucet, A., Godsill, S., Andrieu, C., 2000. On sequential Monte Carlo sampling methods for Bayesian filtering. Stat. Comput. 197–208.

Frackowiak, R. S. 2004. Human brain function. K. J. Friston, C. D. Frith, R. J. Dolan, C. J. Price, S. Zeki, J. T. Ashburner, & W. D. Penny (Eds.). Academic press.

Friston, K.J., Buechel, C., Fink, G.R., Morris, J., Rolls, E., Dolan, R.J., 1997. Psychophysiological and modulatory interactions in neuroimaging. Neuroimage 6, 218–229.

Friston, K. J., Fletcher, P., Josephs, O., Holmes, A. N. D. R. E. W., Rugg, M. D., & Turner, R. ,1998. Event-related fMRI: characterizing differential responses. Neuroimage, 7(1), 30-40.

Greenwald, A.G., Farnham, S.D., 2000. Using the Implicit Association Test to measure self-esteem and self-concept. J. Pers. Soc. Psychol. 79, 1022–1038.

Greenwald, A.G., McGhee, D.E., Schwartz, J.L., 1998. Measuring individual differences in implicit cognition: the implicit association test. J. Pers. Soc. Psychol. 74, 1464–80.

Greenwald, A.G., Nosek, B. a., Banaji, M.R., 2003. Understanding and using the Implicit Association Test: I. An improved scoring algorithm. J. Pers. Soc. Psychol. 85, 197–216.

Johnson, J.D., Muftuler, L.T., Rugg, M.D., 2008. Multiple repetitions reveal functionally and anatomically distinct patterns of hippocampal activity during continuous recognition memory. Hippocampus 18, 975–980.

Josephs, O., & Henson, R. N. ,1999. Event-related functional magnetic resonance imaging: modelling, inference and optimization. Philosophical Transactions of the Royal Society B: Biological Sciences, 354(1387), 1215-1228.

Kriegeskorte, N., Simmons, W.K., Bellgowan, P.S., Baker, C.I., 2009. Circular analysis in systems neuroscience: the dangers of double dipping. Nat Neurosci 12, 535–540.

Mitchell, J.P., Macrae, C.N., Banaji, M.R., 2006. Dissociable Medial Prefrontal Contributions to Judgments of Similar and Dissimilar Others. Neuron 50, 655–663.

Niv, Y., Daniel, R., Geana, A., Gershman, S.J., Leong, Y.C., Radulescu, A., Wilson, R.C., 2015. Reinforcement learning in multidimensional environments relies on attention mechanisms. J. Neurosci. 35, 8145–8157.

O'Reilly, J.X., Woolrich, M.W., Behrens, T.E.J., Smith, S.M., Johansen-Berg, H., 2012. Tools of the trade: Psychophysiological interactions and functional connectivity. Soc. Cogn. Affect. Neurosci. 7, 604–609.

von Fersen, L., Wynne, C.D., Delius, J.D., Staddon, J.E., 1991. Transitive inference formation in pigeons. J. Exp. Psychol. Anim. Behav. Process. 17, 334–341.

Weiskopf, N., Hutton, C., Josephs, O., & Deichmann, R., 2006. Optimal EPI parameters for reduction of susceptibility-induced BOLD sensitivity losses: a whole-brain analysis at 3 T and 1.5 T. Neuroimage, 33(2), 493-504.

Williams, R.J., 1992. Simple statistical gradient-following algorithms for connectionist reinforcement learning. Mach. Learn. 8, 229–256.

Wilson, R.C., Niv, Y., 2015. Is Model Fitting Necessary for Model-Based fMRI? PLoS Comput. Biol. 11.

Wimmer, G.E., Daw, N.D., Shohamy, D., 2012. Generalization of value in reinforcement learning by humans. Eur. J. Neurosci. 35, 1092–1104.

Winston, J.S., Strange, B. a, O'Doherty, J., Dolan, R.J., 2002. Automatic and intentional brain responses during evaluation of trustworthiness of faces. Nat. Neurosci. 5, 277–283.
