# Supplementary material for: Computations Underlying Social Hierarchy Learning: Distinct Neural Mechanisms for Updating and Representing Self-Relevant Information
Source: Neuron. 2016 Dec 7;92(5):1135–47. doi: 10.1016/j.neuron.2016.10.052 (PMC5158095; doi:10.1016/j.neuron.2016.10.052)
Supplement: Document S2. Article plus Supplemental Information [file mmc2.pdf]

Article

Neuron

Computations Underlying Social Hierarchy Learning: Distinct Neural Mechanisms for Updating and Representing Self-Relevant Information

Highlights

- Social hierarchy learning accounted for by a Bayesian inference scheme
- Amygdala and hippocampus support domain-general social hierarchy learning
- Medial prefrontal cortex selectively updates knowledge about one's own hierarchy
- Rank signals generated by these neural structures in absence of task demands

Authors

Dharshan Kumaran, Andrea Banino, Charles Blundell, Demis Hassabis, Peter Dayan

Correspondence

dkumaran@google.com

In Brief

How do we learn the power structure within our social community? Kumaran et al. show that this involves the maintenance and updating of beliefs about individuals' power, with the medial prefrontal cortex specifically supporting knowledge about one's own social hierarchy.

CrossMark

Kumaran et al., 2016, Neuron *92*, 1135–1147
December 7, 2016 © 2016 The Authors. Published by Elsevier Inc.
http://dx.doi.org/10.1016/j.neuron.2016.10.052

CellPress

Computations Underlying Social Hierarchy Learning: Distinct Neural Mechanisms for Updating and Representing Self-Relevant Information

Dharshan Kumaran,[1,3,4,*] Andrea Banino,[1] Charles Blundell,[1] Demis Hassabis,[1,2] and Peter Dayan[2]
[1]Google DeepMind, 5 New Street Square, London EC4A 3TW, UK
[2]Gatsby Computational Neuroscience Unit, 25 Howland Street, London W1T 4JG, UK
[3]Institute of Cognitive Neuroscience, University College London, 17 Queen Square, London WC1N 3AR, UK
[4]Lead Contact
*Correspondence: dkumaran@google.com
http://dx.doi.org/10.1016/j.neuron.2016.10.052

SUMMARY

Knowledge about social hierarchies organizes human behavior, yet we understand little about the underlying computations. Here we show that a Bayesian inference scheme, which tracks the power of individuals, better captures behavioral and neural data compared with a reinforcement learning model inspired by rating systems used in games such as chess. We provide evidence that the medial prefrontal cortex (MPFC) selectively mediates the updating of knowledge about one's own hierarchy, as opposed to that of another individual, a process that underpinned successful performance and involved functional interactions with the amygdala and hippocampus. In contrast, we observed domain-general coding of rank in the amygdala and hippocampus, even when the task did not require it. Our findings reveal the computations underlying a core aspect of social cognition and provide new evidence that self-relevant information may indeed be afforded a unique representational status in the brain.

INTRODUCTION

Considerable evidence suggests that groups of humans, nonhuman primates, and a variety of other species are arranged in linear social dominance hierarchies that are stable over relatively long periods of time. Knowing these relative social ranks is critical for selecting advantageous allies and avoiding potentially dangerous conflicts (Cheney and Seyfarth, 1990; Rushworth et al., 2013). At least two sources of information may be used to guide judgments of social rank. One is the physical appearance of an individual (e.g., facial features and body posture) (Marsh et al., 2004; Todorov et al., 2008; Zink et al., 2008). Such perceptual cues are thought to be widely used in the animal kingdom to indicate rank (e.g., plumage) (Byrne and Bates, 2010) and may provide a relatively coarse heuristic with which to rapidly assess the threat posed by an unfamiliar individual in primates (e.g., an intruder) (Marsh et al., 2004; Todorov et al., 2008; Whalen, 1998). In contrast, the other source of information, namely, experience of previous encounters between (pairs of) individuals, is more robust—albeit potentially incomplete, if not all encounters arise. Research in animals, therefore, has emphasized that rank judgments depend critically on knowledge acquired through learning, coupled with a highly developed capacity for transitive inference (i.e., if A > B and B > C, then A > C) (Byrne and Bates, 2010; Cheney and Seyfarth, 1990; Grosenick et al., 2007; Paz-Y-Miño C et al., 2004).

Although existing work in humans (Kumaran et al., 2012), complemented by research in animals (Noonan et al., 2014; Rushworth et al., 2013), has provided evidence that the amygdala and anterior hippocampus are selectively involved in the acquisition and use of knowledge about a social (i.e., as opposed to a non-social (Kumaran et al., 2012) hierarchy, three important issues remain open. First, what are the neural computations that support the learning of social hierarchies? Although a recent line of research demonstrates that certain aspects of social learning (e.g., about traits, trust games, and theory of mind [ToM]) can be accounted for by reinforcement learning (RL) mechanisms (Behrens et al., 2009; Burke et al., 2010; Hackel et al., 2015; Hampton et al., 2008; King-Casas, 2005; Suzuki et al., 2012), a rich theoretical framework formalizes the acquisition of structured knowledge (e.g., about social hierarchies and networks) in Bayesian terms (Kemp and Tenenbaum, 2008; Tervo et al., 2016). Which accounts better for social hierarchy learning is not clear. Second, previous work has only examined the neural substrates underlying learning about social hierarchies composed exclusively of other individuals (Chiao et al., 2008; Kumaran et al., 2012; Zink et al., 2008). A key question, therefore, is whether learning about dominance relationships within one's own hierarchy—arguably the most relevant type of knowledge in the real world—recruits similar or distinct neural mechanisms. Indeed, this has broader relevance for the fundamental question of whether self-related information is represented by distinct neural mechanisms (Amodio and Frith, 2006; Denny et al., 2012; Mitchell et al., 2006), an issue that has been difficult to answer definitively because of the natural intertwining of the self/other dimension with the richness and quantity of prior knowledge (e.g., in trait judgment tasks). Third, is there automatic

This is an open access article under the CC BY license (http://creativecommons.org/licenses/by/4.0/).

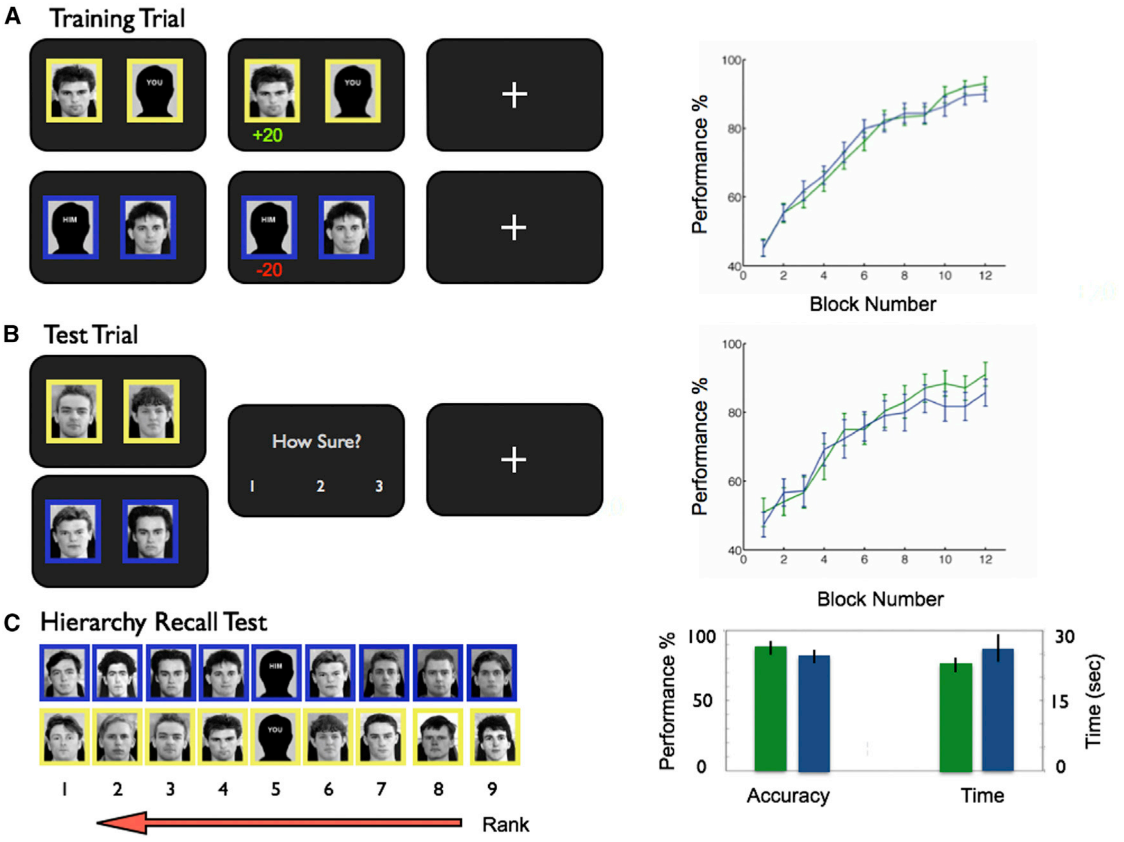

Figure 1. Learn Phase: Experimental Task and Behavioral Data

(A) Training trials: timeline (left), behavioral data (right). Participants viewed adjacent items in the hierarchy: P4 versus P5 illustrated for Self condition (above, yellow border around faces) and Other condition (below, blue border around faces). Yellow or blue (counterbalanced) signified the logo color of the company to which individuals belonged. P5 was the participant or a close friend for the Self and Other conditions, respectively. Participants selected the item they thought had more power and received corrective feedback. Male participants saw only male individuals; female participants saw only female individuals. Right: training trial performance across all 12 experimental blocks, averaged across all eight training trial types (e.g., P1 versus P2, P2 versus P3) and participants (Self condition: green; Other condition: blue; error bars reflect SEM).

(B) Test trials: timeline (left), behavioral data (right). Participants viewed non-adjacent items in the hierarchy (P3 versus P6 illustrated), inferred the higher ranking item, and rated their confidence in their choices; no feedback was provided. Right: performance over all 12 experimental blocks, averaged across all eight test trial types (four of which included the participant or his or her friend [P2 versus P5] and four of which did not [e.g., P3 versus P6]) and participants (Self condition: blue; Other condition: green; error bars reflect SEM).

(C) Hierarchy recall test (debriefing session): pictures of the sets of people in the Self and Other hierarchy conditions were presented to participants, and they were asked to rank them in terms of their order in the hierarchy, with their performance timed. Example Self and Other hierarchies are illustrated (not shown to participants): members of the yellow- and blue-logo companies, respectively. Note that the allocation of person to rank position (1 = high rank, 9 = low rank) was randomized across participants, although P5 was always the participant or a close friend for the Self or Other condition. Right: performance (%) on hierarchy recall test and time taken (seconds) (Self condition: green; Other condition: blue).

generation of neural signals reflecting rank in a social hierarchy (hereafter termed "power" because it is considered a continuous dimension in our study) that was learned through experience, even when the task does not require it? Although previous work (Kumaran et al., 2012) demonstrated that neural signatures of power are generated when needed to perform an evaluation task, ecological evidence suggests that the power of others should be automatically represented, in an analogous fashion to perceptual signals relating to trustworthiness (Engell et al., 2007; Todorov et al., 2008; Winston et al., 2002).

To examine these issues, we developed a new experimental task that builds on a prior study (Kumaran et al., 2012) used to elucidate the neural substrates of hierarchy learning but incorporates extra features. In the "Learn" phase of the task, participants acquired knowledge of two social hierarchies in parallel. Although both hierarchies were comprised of unfamiliar members of two fictitious companies, they were distinguished by incorporating either the participants themselves (hereafter the Self hierarchy) or close friends of the participants (hereafter the Other hierarchy) (see Figure 1). Thus, our experimental design afforded us the opportunity to identify putative differences in the neural mechanisms that support learning about self-related information, decoupled as far as possible from many forms of preexisting and prior knowledge about the self that are inherent in other studies (Denny et al., 2012; Jenkins et al., 2008; Kelley et al., 2002; Macrae et al., 2004; Mitchell et al., 2005, 2006; Tamir

and Mitchell, 2011, 2012). We incorporated a direct test of the effectiveness of our Self/Other manipulation using a specifically tailored version of the classic implicit association test (IAT) (Greenwald et al., 1998; Mitchell et al., 2006) (see below and Supplemental Experimental Procedures). Finally, during a second scanning (''Categorization'') phase, participants viewed pictures of individuals from both hierarchies, allowing us to probe the underlying representations of the hierarchies learned in the previous phase and identify regions that automatically generate signals of power, even when the task does not require it.

In sum, our experiment was specifically set up to elucidate the computational mechanisms operating during social hierarchy learning, examine whether distinct neural processes support the learning and representation of self-relevant information (i.e., the power of individuals within one's own, compared with another's, social group), and determine whether signals reflecting individuals' power are automatically generated even in the absence of task demands.

RESULTS

Learn Phase

During the Learn phase, participants completed training trials in each of which a pair of adjacent people in the hierarchy was presented (e.g., P1 versus P2, where P = person; Figure 1): they were required to learn through trial and error which person had more power in each of two hierarchies relating to different companies signified by colored logos (i.e., yellow and blue; each company consisted exclusively of people of the same gender as the subject). One hierarchy included the subject himself or herself (the Self condition), and the other incorporated a close friend (the Other condition; see Supplemental Experimental Procedures for the elicitation procedure). Our experimental design incorporated a close friend in the Other condition, rather than an acquaintance or unfamiliar other individual, in order to render these conditions as similar as possible, thereby isolating the Self/Other dimension (e.g., Mitchell et al., 2006). Following each training trial block, participants completed test trials in which they were required to select the more powerful of the two items presented (e.g., P4 versus P6; Figure 1B) and rate their confidence in their decisions on a scale ranging from 1 (guess) to 3 (very sure). Test trials, therefore, differed from training trials in two critical ways: non-adjacent items in the hierarchy were presented during test trials (e.g., P4 versus P6), and no corrective feedback was issued (although subjects knew that they would ultimately be remunerated for correct answers). Confidence judgements did not attract compensation. Importantly, participants could not rely on memorization (i.e., of the item from each training pair associated with the positive outcome) to perform successfully during test trials but instead were required to deduce the correct item using knowledge of the underlying hierarchy.

Behavioral Results

Participants improved their performance on training and test trials over the course of the Learn phase: no significant difference was found between Self and Other conditions in terms of reaction times (RTs) (Self: 1.49 [0.04] s and 1.52 [0.04] s; Other: 1.49 [0.04] s and 1.54 [0.05] s; training and test trials respectively, SEM in brackets), the correctness of choices, or confidence ratings (all p values > 0.2; Figures 1A and 1B). Following scanning, participants were also tested on their explicit knowledge of the hierarchy (hierarchy recall test; see Figure 1C and Experimental Procedures). They performed proficiently (Figure 1C), with no significant difference between conditions in terms of accuracy (Self versus Other: 92.1% [2.7%] versus 84.1% [4.4%], mean with SEM in brackets) or time taken (Self versus Other: 22.7 s [2.1 s] versus 25.6 s [3.1 s], mean with SEM in brackets) (both p values > 0.2).

Following scanning, participants also completed a version of a classical psychological paradigm, the implicit association test (see Figures 2 and S1 and Supplemental Experimental Procedures) (Greenwald et al., 1998), which we adapted to probe the extent to which participants incorporated themselves into the Self hierarchy condition. There was a highly significant IAT effect, evidenced by speeding of RTs in congruent trials (congruent: 690 ms [152.8 ms]; incongruent: 856 ms [30.2 ms]; $t_{23} = 5.33$, $p < 0.001$; Figure 2; see Supplemental Experimental Procedures). This evidence was complemented by related subjective measures obtained through a debriefing questionnaire, in which participants reported that they incorporated themselves in the Self condition and their friends in the Other condition, to a similar degree ($p > 0.1$) (see Table S8). These results demonstrate the effectiveness of our experimental manipulation in the Self versus Other condition, consistent with previous evidence that merely assigning participants into different groups in an arbitrary fashion can have substantial effects on behavior (i.e., higher monetary sharing within a group, compared with across groups, in Volz et al., 2009).

Computational Modeling

Existing models of transitive inference have been typically been restricted in being able to learn only relatively small hierarchies (i.e., six or fewer items) (Frank et al., 2003; von Fersen et al., 1991). We therefore developed two novel models capable of learning long hierarchies (i.e., here of nine items): one involving (approximate) Bayesian inference and the other involving reinforcement learning (see Supplemental Experimental Procedures).

According to the first model, subjects treat the powers of individuals as a hidden or latent variable, about which they make approximate Bayesian inferences. These inferences are based on the likelihood of observations (i.e., the outcomes of training trials that reveal which individual has more power). Given the finding that participants required approximately 200 trials to achieve proficiency (see Figures 1A and 1B), despite receiving deterministic feedback during training, we modeled them as having imperfect memory (as might, for instance, arise from a changing environment). For a concrete implementation of forgetful Bayesian inference, we used an example of a popular class of filtering algorithms known as sequential Monte Carlo (SMC) methods (Doucet et al., 2000). These aim to infer the underlying distribution of an evolving hidden variable, representing it through a set of notional samples or particles. Forgetfulness is straightforward to capture via a parameter (called σ) in the SMC model, which influences the tendency for particles to drift

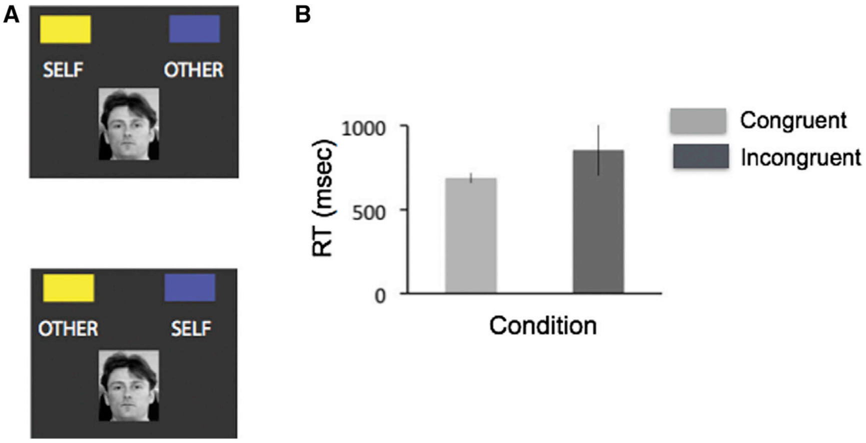

Figure 2. Implicit Association Test: Evidence that Participants Incorporated Themselves into the Self Hierarchy

(A) Experimental design. Top: congruent condition: when the yellow logo (i.e., the color of the Self company logo in this example) is displayed on the same side as the word "Self" and when the blue logo (i.e., the color of the Other company) is displayed on the same side as the word "Other." Bottom: incongruent condition: when the yellow logo is displayed on the side of the word "Other." The rationale is that if participants have incorporated themselves into their own social group, they should be faster to categorize faces according to company membership in the congruent (cf. incongruent) condition, in which the word "Self" is on the same side as the color of the company logo (i.e., yellow). In contrast, RTs should be slower in the incongruent condition, in which the yellow logo is displayed on the side of the word "Other," because of a Stroop-like effect (see Supplemental Experimental Procedures). Note that the words "Self" and "Other" were not presented to participants during the experiment: profile pictures were denoted by "you" and "him" or "her."

(B) Mean latencies for the congruent (light gray) and incongruent (dark gray) trials, averaged across participants (see Supplemental Experimental Procedures for details of analytic procedure). The IAT effect is the difference in response times between congruent trials and incongruent trials (error bars reflect SEM).

over time (see Supplemental Experimental Procedures). This prevents asymptotic certainty and slows learning.

The second method involved RL. Typical RL methods that assign values to items on the basis of their propensity to be associated with a rewarding outcome (e.g., the Rescorla-Wagner rule or Q learning; Watkins and Dayan, 1992) are known to fail in hierarchy learning tasks. This is because each item (apart from the top and bottom ranked) is equally associated with positive and negative outcomes during training trials. Consequently, in developing an RL account (hereafter termed RL-ELO) capable of successful hierarchy learning performance, we sought inspiration from algorithms used to update player ratings in games (e.g., Yliniemi and Tumer, 2013) (e.g., the ELO rating system in chess; see Supplemental Experimental Procedures), whose critical component is to increase or decrease the rating (i.e., power) of the winning or losing individual in a pairwise contest or trial as a function of the rating of one's opponent (i.e., the winner has a relatively small update if the opponent was estimated to be much less powerful). This has been proved to work even in gargantuan problems.

A critical qualitative difference between the SMC and RL-ELO schemes concerns uncertainty. The SMC model inherently models the uncertainty in the estimation of power. In contrast, the RL-ELO, like temporal difference (TD) learning, maintains only a single scalar estimate of power for each individual (e.g., Niv et al., 2006; Schultz et al., 1997; although see Gershman, 2015). Furthermore, the models differ in the nature of the mechanism by which they update their estimates of the power of individuals within the hierarchy (see below).

One principal aim of this study was to determine which of these two models, the SMC mechanism or the RL-ELO mechanism, was better able to capture participants' data. At a behavioral level, we quantified the fit of each of the SMC and RL-ELO models, as well as previously developed models (value transfer and Rescorla-Wagner), to participants' choice behavior during training and test trials. Using a maximum likelihood estimation procedure to optimize a separate set of parameters for each participant (see Daw, 2011; Wimmer et al., 2012), we found strong evidence (see Table 1) in favor of the SMC model according to the log likelihood of each model and the corresponding Bayesian information criterion (BIC) measure (Raftery, 1995). We also examined a variant of the RL-ELO model, termed RL-ELO_F, which incorporated an extra parameter (i.e., σ) to allow forgetting (i.e., through the addition of Gaussian noise at each time step); this did not significantly improve the fit of the RL model indexed by BIC scores (see Table 1).

Interestingly, the difference between our two primary models arose in the trials in the first half of the experiment (BIC = 185.0 versus 201.8 for SMC versus RL-ELO) rather than the second half (BIC = 109.3 versus 111.7, respectively), consistent with the notion that the SMC model captures behavior more effectively than the RL model when participants are more uncertain about the relative power of individuals during the early phase of learning. This is explained by a qualitative difference between the updating mechanisms of the models: the RL-ELO model only updates the values of the current items in a trial (i.e., unconstrained by the values of the other items). In contrast, the SMC model naturally updates the values of items not present in a given training trial, because it updates a posterior distribution over all items on a trial-by-trial basis, because each particle constitutes a particular belief about the values of all nine items in the hierarchy. The reason that the difference in model fit is greatest in the first half of the experiment is that this is the period when most learning and hierarchy updating is occurring (e.g., see Figure 1). This difference in updating mechanism makes the specific prediction that the RL-ELO model should be much more sensitive (i.e., in terms of influence on its predicted choices) than the SMC model to the particular reinforcement history of items and therefore trial order experienced. We provide evidence for this predicted difference (see Figure S2: a significantly greater effect of reinforcement history on negative log likelihood [NLL] for the RL [cf. SMC] model, $Z = 24.8$, $p < 0.0001$). Notably, the difference in updating mechanism also results in the SMC model's being able to more rapidly converge on the correct

Table 1. Results of Behavioral Model Fitting

Model	Condition	−LL	BIC	α	β	σ	θ
SMC	Self	136	283	–	0.64 (0.12)	0.11 (0.03)	–
	Other	137	285	–	0.65 (0.15)	0.15 (0.04)	–
RL-ELO	Self	144	299	1.35 (0.12)	1.83 (0.22)	–	–
	Other	146	303	1.31 (0.15)	1.39 (0.08)	–	–
RL-ELO$_F$	Self	141	299	1.16 (0.22)	1.22 (0.23)	0.11(0.02)	–
	Other	142	301	1.06 (0.20)	1.18 (0.22)	0.13 (0.03)	–
Value transfer	Self	160	331	0.11 (0.01)	0.13 (0.01)	–	0.22(0.02)
	Other	161	333	0.10 (0.01)	0.14 (0.01)	–	0.19 (0.02)
RW	Self	191	393	0.05 (0.01)	0.37 (0.03)	–	–
	Other	191	393	0.05 (0.01)	0.46 (0.08)	–	–
Base	Self	200	400	–	–	–	–
	Other	200	400	–	–	–	–

−LL, negative log likelihood. Average quantities reported. Models fit individually to participants. Mean (SEM) of best fitting parameters for each individual reported (see Supplemental Experimental Procedures for description of models and parameters).

rank ordering: the difference between model fits (i.e., in terms of negative log likelihood) showed a highly significant correlation with subjects' performance, with higher performing subjects showing greater advantage for the SMC model (Pearson's correlation: $R = -0.75$ and -0.79 [$p < 0.0001$] for the Self and Other conditions). Notably, the vast majority of subjects were better fit by the SMC model (22 and 23 of 28 subjects in the Self and Other conditions).

The task affords two additional measures by which the models can be compared: reaction times and confidence judgments. We expect these to be related to the uncertainty associated with the choices, captured by a trial-by-trial internal variable common to both SMC and RL-ELO models, namely, choice entropy (see Supplemental Experimental Procedures). A linear mixed-effects model demonstrated that the SMC model also provided a superior fit to participants' reaction time data, compared with the RL-ELO model during both training (SMC, BIC = 8,492; RL-ELO, BIC = 8,699) and test (SMC, BIC = 4,491; RL, BIC = 4,520) trials, in which a choice entropy was entered as a fixed effect, and participant and condition (Self or Other) were entered as random effects (likelihood ratio compared with the null model, all p values $< 1 \times 10^{-15}$). The choice entropy from the SMC model also captured the confidence of participants in their responses during transitive test trials more proficiently (SMC, BIC = 8,687; RL-ELO, BIC = 8,733; likelihood ratio versus null model, all p values $< 1 \times 10^{-15}$).

Neuroimaging Data

Neural Activity in the Amygdala and Anterior Hippocampus Correlates with SMC-Modeled Difference between the Power of Individuals during Test Trials

Given our finding that the SMC model best accounts for participants' behavior (i.e., choices, RT, confidence data), we next probed the neural data using its key internal variables. We first focused on the test trial data, in which participants were presented with item pairs not seen during training trials (e.g., P3 versus P6), were not given corrective feedback, and were therefore required to use their estimates about individuals' power. We sought to identify regions where neural activity tracked the (expectation over the) difference between the modeled distributions of the power of items in a given trial (see Figure 3A). To achieve this, we created participant-specific trial-by-trial parametric regressors reflecting the unsigned power difference between items which were used to regress against the fMRI data (see Supplemental Experimental Procedures).

We found a robust correlation between neural activity in the amygdala, hippocampus, ventromedial prefrontal cortex (vMPFC), and the difference between the power of individuals as estimated by the SMC model (see Figure 3B and Table S1). We also observed a tight correlation between neural activity and item power difference in a region proximate to the fusiform face area (FFA) (see Figure 3B). Results from an region-of-interest (ROI) analysis performed separately in the Self and Other conditions provide evidence that the left amygdala (Self: $t_{27} = 1.95$, $p = 0.028$; Other: $t_{27} = 1.73$, $p = 0.044$), ventromedial prefrontal cortex (Self: $t_{27} = 2.27$, $p = 0.01$; Other: $t_{27} = 3.14$, $p = 0.0013$), and FFA-proximate region (Self: $t_{27} = 2.75$, $p = 0.005$; Other: $t_{27} = 3.33$, $p = 0.0011$) code SMC-modeled differences in power that support performance during test trials in both conditions. Notably, these effects cannot be driven by differences in reaction times between trials given that an earlier regressor in the same general linear model captured such effects (see Experimental Procedures). Together with a previous study (Kumaran et al., 2012), these findings provide evidence that the anterior hippocampus and amygdala play a specific role in social rank judgements: indeed, activity in this region identified in this previous study to be associated with model-agnostic measures of hierarchy learning (i.e., social > non-social contrast, shown in Figure 2B of Kumaran et al., 2012; ROI defined at $p < 0.005$ uncorrected) showed a robust correlation with the SMC-modeled difference in power during test trials in the present study ($t_{27} = 6.17$, $p < 0.000001$).

Hierarchy Updating: The SMC Model Provides a Superior Fit to Neural Data Compared with the RL Model

Previous results provide evidence for shared signals in the amygdala and hippocampus during performance of test trials, relating

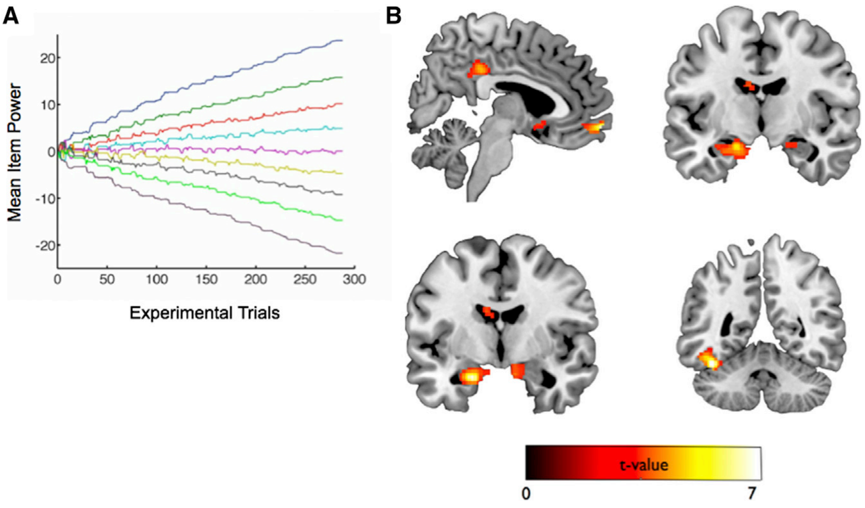

Figure 3. Learn Phase: Neural Activity in the Amygdala, Hippocampus, and vMPFC Correlates with SMC-Estimated Difference in Individuals' Power during Test Trials in Self and Other Conditions

(A) Illustrative plot from a participant showing the evolution over the experiment of the mean (i.e., expectation over the distribution of) power relating to each of the nine individuals in the hierarchy.

(B) Activity in the bilateral amygdala (top right), vmPFC and posterior cingulate cortex (top left), bilateral anterior hippocampus (bottom left), and region proximate to the fusiform face area (bottom right) shows a significant correlation with SMC-modeled absolute difference between individuals' power in test trials. Activations are thresholded at $p < 0.005$ uncorrected for display purposes but significant in all regions at $p < 0.001$ uncorrected and $p < 0.05$ whole-brain FWE corrected at peak or cluster level. See Table S1 for a full list of activations.

to both one's own group (Self condition) and another's group (Other condition). We next turned our focus to training trials, in which participants had the opportunity to update their beliefs about the power of individuals in the Self and Other hierarchies (i.e., on the basis of corrective feedback): this provided the basis of successful performance in test trials. To identify signatures of learning, we sought regions in which neural activity showed a correlation with an internal measure, termed the hierarchy update index (Figure 4A) (see Supplemental Experimental Procedures) reflecting the degree to which participants updated their estimates of the power of individuals from trial to trial. Specifically, for each pair of items (e.g., P1 versus P2) we computed the Kullback-Leibler divergence between the participants' estimates of the probability of one item winning against the other before and after feedback, summing this quantity across all pairs (see Supplemental Experimental Procedures). Note that the correlation between the hierarchy update index and other measures (e.g., chosen power) was relatively low (~0.2).

In a whole-brain analysis collapsed across both Self and Other conditions, we found a robust correlation between MPFC activity and the SMC-modeled hierarchy update index (MPFC: x, y, and z coordinates −8, 44, and 10; $Z = 5.04$, family-wise error peak level corrected $p = 0.022$) and also with the hippocampus and the FFA-proximate region (see Table S3A). We also identified significant correlations in a targeted ROI analysis in the MPFC ($t_{27} = 1.97$, $p = 0.030$), left amygdala ($t_{27} = 1.73$, $p = 0.047$), bilateral hippocampus (left: $t_{27} = 3.70$, $p = 0.00048$; right: $t_{27} = 2.77$, $p = 0.005$), and FFA-proximate region ($t_{27} = 3.28$, $p = 0.0014$) but not the vmPFC ($p > 0.1$). When the Self and Other conditions were considered separately, significant effects were present in the hippocampus (Self: left hippocampus $t_{27} = 3.10$, $p = 0.0022$, right hippocampus $t_{27} = 2.94$, $p = 0.0033$; Other: left hippocampus $t_{27} = 1.93$, $p = 0.032$).

Previously we showed that the SMC model fit the behavior more proficiently than the RL-ELO model and provided evidence of their qualitative difference in hierarchy-updating mechanism (e.g., see Figure S2). Here we compare the fit of these models to the neural data, using model-derived hierarchy update regressors, collapsed across Self and Other conditions, in regions of interest defined anatomically and functionally (see Supplemental Experimental Procedures) (Ashby and Waldschmidt, 2008; Niv et al., 2015; Wilson and Niv, 2015). The relative differences in BIC between models (Ashby and Waldschmidt, 2008; Raftery, 1995) provide strong support that the SMC model best captures neural activity in amygdala, hippocampus, and MPFC (see Table S2).

Activity in the MPFC Correlates with SMC-Modeled Hierarchy Update Signal in the Self Condition during Training Trials

We found that the correlation between MPFC activity and the hierarchy update index was driven by the Self condition (whole-brain analysis: MPFC: x, y, and z coordinates −6, 46, and 12; $Z = 4.22$, $p < 0.001$ uncorrected and small-volume corrected [SVC] $p = 0.0040$; MPFC ROI analysis, $t_{27} = 2.73$, $p = 0.0055$; Figure 4B and Table S3B). This finding remained robust when we restricted our analyses to just those trials in which participants updated their knowledge about other individuals in the hierarchy, excluding themselves and their friends (i.e., P4 versus P5, P5 versus P6; see Supplemental Experimental Procedures) (whole-brain analysis: Self: Montreal Neurological Institute [MNI] x, y, and z coordinates −6, 46, and 12; $Z = 4.25$, $p < 0.001$ uncorrected and SVC $p = 0.0030$; MPFC ROI analysis, $t_{27} = 2.93$, $p = 0.0034$). No significant correlations were observed in the MPFC in these analyses in the Other condition (ROI analyses: p values > 0.2). Further, an ROI analysis showed that there was a greater correlation between MPFC activity and the hierarchy update index in the Self compared with the Other condition (Self > Other: $t_{27} = 1.83$, $p = 0.039$), with no significant differences found in this analysis in other regions (i.e., hippocampus, amygdala, and FFA-proximate region: all p values > 0.1). No significant differences were found in the reverse contrast (i.e., Other > Self) in either a whole-brain analysis or ROI analysis (all p values > 0.2).

Furthermore, we found a robust between-subjects correlation between performance (i.e., averaged across this experimental phase) and the strength of the correlation between MPFC activity and the hierarchy update index in the Self condition. This was the

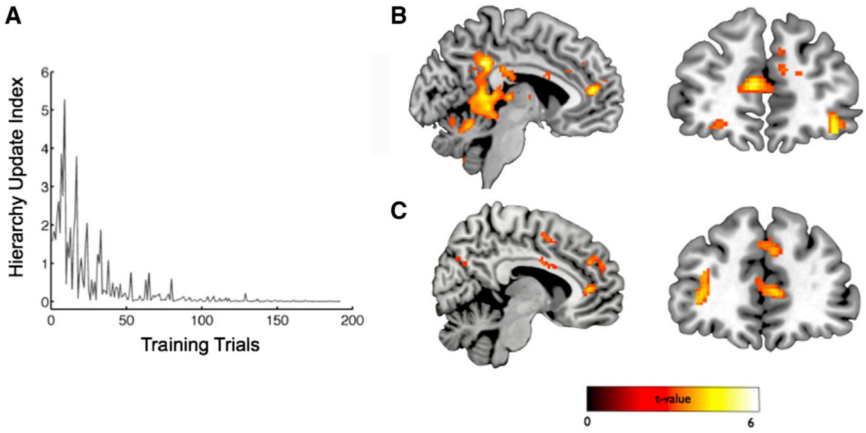

Figure 4. Learn Phase: MPFC Activity Correlates with SMC-Modeled Hierarchy Update Signal Selectively in Self Condition

(A) Illustrative example from one subject showing profile of SMC-modeled hierarchy update index during training trials (see Supplemental Experimental Procedures for details).

(B) Whole-brain analysis: significant correlation between activity in MPFC and hierarchy update index in Self condition (sagittal and coronal views: MNI x, y, and z coordinates −6, 46, and 12; $Z = 4.22$, $p < 0.001$ uncorrected and SVC $p = 0.0040$) (Table S3B).

(C) Significantly greater correlation between MPFC activity and updating of objective measure of hierarchy knowledge (i.e., negative log likelihood of responding correctly on all possible pairs of individuals) in Self compared with Other condition: sagittal (left) and coronal (right) sections shown (MPFC x, y, and z coordinates 2, 44, and 8; $Z = 3.87$, $p < 0.001$ uncorrected and SVC $p = 0.021$). Display threshold is $p < 0.005$ uncorrected.

case at the whole-brain level in the MPFC in the Self condition (x, y, and z coordinates −8, 42, and 8; $p < 0.001$ uncorrected and SVC $p = 0.043$) and in the MPFC ROI ($t_{27} = 2.07$, $p = 0.024$; trend in L amygdala ROI $t_{27} = 1.61$, $p = 0.06$). No such correlation was found in the MPFC ROI in the Other condition, with a significantly greater correlation in the Self condition (Self > Other: $Z = 1.85$, $p = 0.032$).

This hierarchy update analysis was based on participants' subjective estimates of the power of individuals at a given moment: we also derived an analogous measure capturing the trial-by-trial change in participants' objective knowledge of the ground truth hierarchy. Although this quantity cannot be directly computed by participants, it was highly correlated with the hierarchy update index (i.e., ∼0.8) and yielded robust differences between the Self and Other conditions in the MPFC in a whole-brain analysis (Self > Other: whole-brain analysis MPFC x, y, and z coordinates 2, 44, and 8; $Z = 3.87$, $p < 0.001$ uncorrected and SVC $p = 0.021$, see Figure 4C: MPFC ROI analysis $t_{27} = 3.56$, $p = 0.00064$). Of note, no significant correlations with this objective hierarchy update index were found in the MPFC in the Other condition (ROI analyses: p values > 0.2).

Selective Coupling between MPFC and Amygdala and Hippocampal Activity during Updating of Hierarchy Knowledge in the Self Condition

Our results show that neural activity in the MPFC specifically correlates with updating of hierarchy knowledge in the Self condition, with significant effects in the amygdala and hippocampus observed across both Self and Other conditions. We next asked whether neural activity in this part of the MPFC and the amygdala and hippocampus, regions that are thought to be anatomically connected (Beckmann et al., 2009; Carmichael and Price, 1995), exhibit greater functional coupling during updating of hierarchy knowledge in the Self compared with the Other condition.

To do this, we performed a psychophysiological (PPI) analysis (Friston et al., 1997; O'Reilly et al., 2012) (see Supplemental Experimental Procedures for details): this was specifically set up to ask in which brain regions the magnitude of functional coupling of neural activity with the MPFC shows a significantly greater correlation with the amount by which hierarchy knowledge changes in the Self compared with the Other condition, above and beyond that explained by differences in the basic correlation between the hierarchy update index in the Self and Other conditions (i.e., hierarchy update: Self > Other, as reported in the preceding analysis). We observed significant effects in the amygdala and hippocampus both in a whole-brain analysis (see Table S4) and in targeted ROI analyses (left hippocampus $t_{27} = 2.35$, $p = 0.010$, right hippocampus $t_{27} = 1.65$, $p = 0.051$; left amygdala $t_{27} = 2.14$, $p = 0.020$, right amygdala $t_{27} = 2.00$, $p = 0.028$). These results provide evidence for selective coupling between the MPFC and the amygdala and hippocampus during updating of hierarchy knowledge in the Self condition.

Correlation between Neural Activity in Amygdala, Hippocampus, and MPFC and SMC-Modeled Power of Chosen Individual during Training Trials

Having established that neural activity in the MPFC correlates with updating of hierarchy knowledge in the Self condition, we next asked whether another internal variable of the SMC model, specifically, the expectation of the distribution (i.e., mean) of power of the chosen item (Figure 5A), was reflected in neural activity (see Supplemental Experimental Procedures, Table S5, for a separate analysis relating to another internal variable: the entropy over item pairs). We first performed an analysis collapsed across Self and Other conditions (i.e., main effect): ROI analyses provided evidence that chosen power was represented in the amygdala (left: $t_{27} = 2.62$, $p = 0.0071$; right: $t_{27} = 1.75$, $p = 0.046$) and ventromedial prefrontal cortex ($t_{27} = 3.19$, $p = 0.0018$) (see Table S6A for results of whole-brain analysis).

We also observed significant differences between the Self and Other conditions in terms of the correlation of neural activity with chosen power. In a whole-brain analysis, we found that neural activity, in a similar region of MPFC to that revealed by the hierarchy update index analysis above, was significantly correlated with trial-by-trial chosen power in the Self condition (MNI x, y, and z coordinates 4, 44, and 2; $Z = 3.01$, SVC $p = 0.037$; see Table S6B) but not the Other condition (see Table S6C). Moreover, there was a significant difference between the Self and Other conditions in

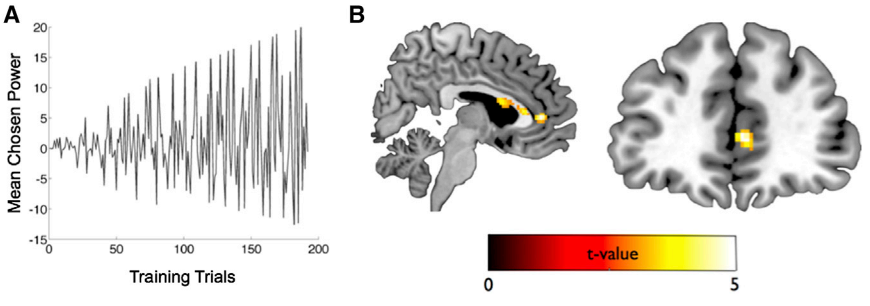

Figure 5. Learn Phase: Correlation between Neural Activity in the MPFC and SMC-Modeled Chosen Power during Training Trials: Self versus Other

(A) SMC-modeled (expectation over) posterior distribution of chosen power for illustrative subject.

(B) Results of whole-brain analysis: region of MPFC identified by correlation of neural activity with SMC modeled chosen power: Self > Other (MNI x, y, and z coordinates: 6, 42, and 4; $Z = 4.23$, significant at SVC $p = 0.001$ and $p < 0.001$ uncorrected; see Table S6D). Display threshold is $p < 0.005$ uncorrected.

the MPFC (see Figure 5B and Table S6D). Furthermore, this finding remained robust when we restricted our analyses to only those trials that did not involve the participant or his or her friend (Self > Other: MNI x, y, and z coordinates 6, 42, and 4; $Z = 4.31$, $p < 0.001$ uncorrected and SVC $p = 0.001$). No region showed significant differences in the reverse contrast (i.e., Other > Self).

Similarly, in an ROI analysis based on this region of the MPFC, we found significant differences between the Self and Other conditions during test trials that did not involve the participant or his or her friend (see Supplemental Experimental Procedures). Specifically, we found evidence for a difference in the correlation between neural activity and the trial-by-trial SMC-modeled difference in individual's power within the MPFC ROI (Self > Other $t_{27} = 1.69$, $p = 0.050$), though not within amygdala, hippocampal, vmPFC, and FFA-proximate ROIs (all p values > 0.1). Together, these analyses, by focusing only on trials that did not involve the participants themselves, suggest that the MPFC supports the updating and representation of power information about other individuals (i.e., rather than only oneself), when these individuals are part of our own social group rather than another group (i.e., Self > Other) (see Discussion).

Categorization Phase

Behavioral Results

In the next phase of the experiment, we aimed to probe participants' representations of the underlying hierarchies and to examine differences between those involving oneself versus a close friend. Participants completed an incidental categorization task that did not require the retrieval of information about power (Figure 6A), allowing us to ask whether signals relating to individuals' power were automatically generated even in the absence of explicit task demands. Individual pictures of people in the Self and Other hierarchies (with the exception of the profile pictures of participants and close friends) were presented, and participants were required to determine to which company individuals belonged (i.e., the yellow or blue logo, assignment counterbalanced). Participants performed this categorization task accurately (Self: 81.8% [2.8%], Other 79.7% [2.96%]; Self reaction time: 0.80 s [0.010 s]; Other reaction time: 0.80 s [0.013 s]; all p values > 0.1). There was no difference in accuracy or RT as a function of rank ($p > 0.1$).

Neuroimaging Data

We first set up a parametric model to identify regions whose activity exhibited significant linear correlations with the rank of individual people in the true underlying hierarchy. We found that neural activity in the left amygdala and anterior hippocampus showed a significant positive correlation with rank across Self and Other conditions (Figures 6B and 6C; Table S7). This provides novel evidence that these neural structures automatically generate rank signals even when the task does not require it and complements studies showing obligatory processing of perceptual cues of trustworthiness (though not dominance), whereby less trustworthy faces elicit higher levels of amygdala activity (Todorov et al., 2011; Winston et al., 2002). No region showed a significant negative correlation with rank. The finding that less powerful (i.e., lower ranked) individuals elicited higher levels of activity is consistent with previous work examining signals relating to valence and dominance of faces based on physical appearance (Todorov et al., 2011), rather than associative learning (i.e., as in this study).

We also observed a significant linear correlation between rank and neural activity in the MPFC ROI derived from the Learn phase ($t_{27} = 1.86$, $p = 0.036$; Figure 7) in the Self condition, providing a parallel to the automatic valuation of items observed in the vmPFC (Lebreton et al., 2009). No such correlation was observed in the Other condition ($p > 0.2$). Although the difference between this linear correlation between Self and Other conditions was not significant in the MPFC ROI, we did observe a significant interaction when rank extremes were considered (i.e., Self/Other × top/bottom rank: $t_{27} = 1.98$, $p = 0.029$), reflecting the fact that MPFC activity distinguished between highest and lowest ranks selectively in the Self condition. No such effect was present in any of the other ROIs (amygdala, hippocampus, or vmPFC; all p values > 0.1).

DISCUSSION

Although social hierarchies are a fundamental organizing structure of primate social groups, little is understood about the computations underlying learning and also whether there are distinct neural mechanisms that support the ability of primates to judge the rank of others within their own social groups, compared with other social groups. To address these questions, we first developed two novel hierarchy learning models, one based on reinforcement learning (RL-ELO) and the other on approximate Bayesian inference (SMC). We showed that participants' behavior and neural data were better captured by the Bayesian inference scheme, which inherently computes the uncertainty about estimates of power (i.e., rank in a continuous dimension), than by the uncertainty-insensitive RL model. We demonstrate

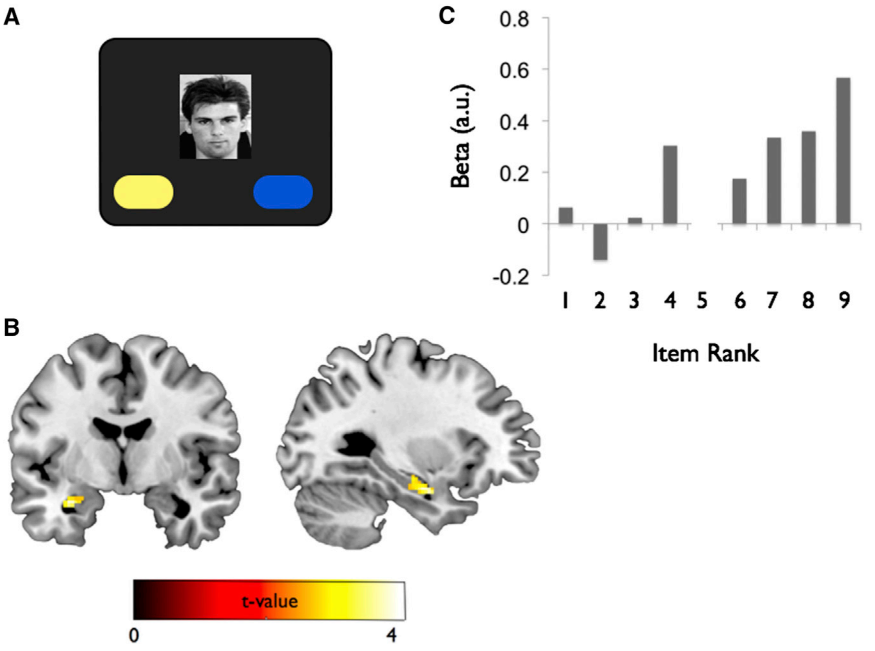

Figure 6. Categorization Phase: Amygdala and Anterior Hippocampus Automatically Generate Rank Signals: Linear Correlation with Neural Activity

(A) Paradigm: during the Categorization phase, participants viewed individuals from the Self and Other hierarchies (with the exception of the profile pictures denoting themselves and their friends; each picture repeated four times) and categorized them according to the company to which they belonged (i.e., the company with the yellow or blue logo).

(B) Activity in the left amygdala and anterior hippocampus shows a linear correlation with rank (main effect: Self and Other conditions). Display $p < 0.005$; significant at $p < 0.001$ uncorrected and L amygdala (SVC $p = 0.025$), and L hippocampus (SVC $p = 0.034$) (see Table S7).

(C) Parameter estimates from peak voxel in main effect (i.e., collapsed across Self and Other conditions) L amygdala and hippocampus (see Table S7). Significant linear correlation evident, with lower ranks (i.e., where 9 = lowest) eliciting highest neural activity. Note that these plots were derived from an "illustrative" model (see Supplemental Experimental Procedures). Statistical inference, however, was based strictly on the parametric model (see Supplemental Experimental Procedures). Note that rank 5 is denoted by an empty slot, because the profile pictures denoting the participants themselves (Self condition) or their friends (Other condition) were not presented.

that learning about one's own social hierarchy, as opposed to that of a close friend, was associated with distinct correlations between internal variables of the SMC model and MPFC activity, while shared signals for both hierarchy types were present in the amygdala and hippocampus. Furthermore, we found that the MPFC was selectively engaged during updating of knowledge about one's own hierarchy, a process that explained variance in participants' performance, and involved functional interactions with the amygdala and hippocampus. Finally, we show that neural signals that automatically represent the power of individuals were generated by the hippocampus and amygdala even when the task did not require it, with power-related activity in the MPFC specific to self-relevant hierarchies.

Emerging evidence suggests work that RL models that do not maintain explicit estimates of uncertainty about decision variables are able to capture behavior and neural data in a wide range of settings, including experiments involving trust games (Hackel et al., 2015; King-Casas, 2005), theory of mind (Hampton et al., 2008), and inferring the preferences and actions of others (Burke et al., 2010; Suzuki et al., 2012). A recent study (Hackel et al., 2015) demonstrated that learning about the generosity of an individual on the basis of informative feedback reflecting his or her propensity to share a monetary endowment was well captured at the behavioral and neural levels by a reinforcement learning model. These findings raise the question of whether learning about the power of individuals in a social hierarchy—another trait-level characteristic like generosity, for which learning is typically feedback-based (i.e., ecologically through observation of the outcome of dyadic contests, mirrored experimentally by training trials in our task)—could also be mediated by an RL process maintaining scalar quantities. Our study, however, provides compelling evidence against this hypothesis. Specifically, we found that the SMC model provided a quantitatively better fit than the RL model across behavioral (i.e., choice, RT, and confidence data) and neural levels. Of course, this provides evidence for the key differentiating constructs underlying SMC, namely, forgetful, uncertainty-sensitive inference. Other approximate Bayesian implementations could lead to the same behavior and neural signals.

The current results dovetail with previous work that used a related paradigm to show that the anterior hippocampus and amygdala as identified here—regions that are coupled by massive bidirectional connectivity (Fanselow and Dong, 2010)—play a specific role in the acquisition of knowledge about social hierarchies (Kumaran et al., 2012). That study, however, was not able to investigate the computational mechanisms underlying learning; rather, neural activity in these regions was correlated directly with model-agnostic behavioral indices of learning. In contrast, our work implies that the anterior hippocampus and amygdala supports social hierarchy learning by maintaining and updating beliefs about the power of individuals through the operation of a probabilistic model, evidenced by significant correlations between neural activity in these structures and internal variables of the SMC model (e.g., the differences in power between individuals during test trials). Indeed, the higher order representation of the uncertainty in one's beliefs naturally sustained by probabilistic models (compared with RL approaches) may be advantageous in deciding whether to approach or avoid an individual on the basis of estimated differences in power. Furthermore, our finding that the hippocampus supports such model-based representations of social hierarchies connects with the notion that these structures constitute

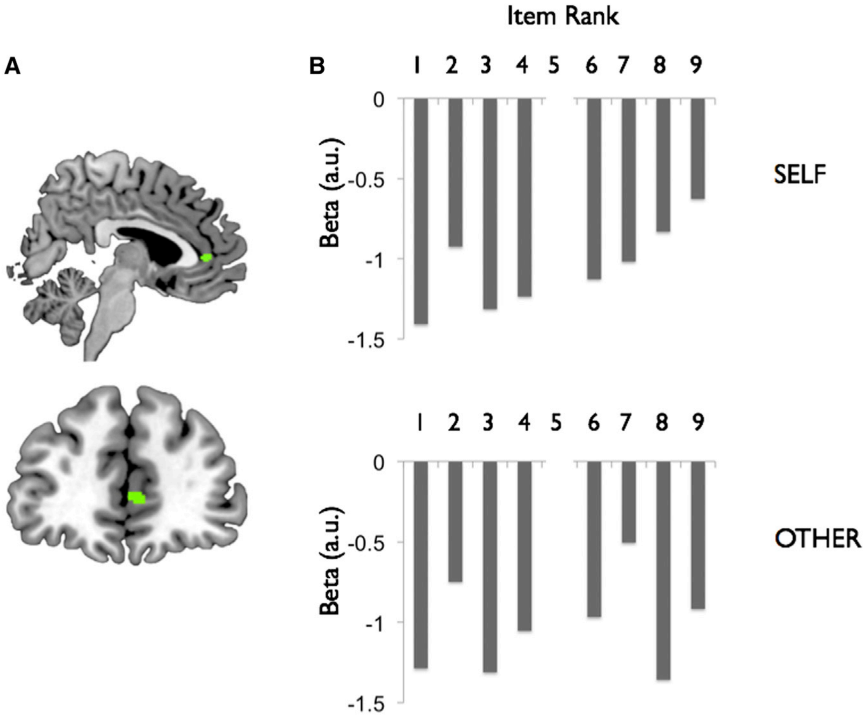

Figure 7. Activity in MPFC Shows a Linear Correlation with Rank in the Self Condition during the Categorization Phase

(A) Region of MPFC defined on the basis of the results of a separate fMRI phase (i.e., learning; see Supplemental Experimental Procedures for details).

(B) Parameter estimates averaged across MPFC ROI in the Self condition (above) and Other condition (below). A significant linear correlation between neural activity and rank in the Self but not the Other condition is evident. These plots were derived from an "illustrative" model; however, statistical inference was based strictly on the parametric model (see Supplemental Experimental Procedures). Note that rank 5 is denoted by an empty slot, because the profile pictures denoting the participants themselves (Self condition) or friend (Other condition) were not presented.

a cardinal example of relational knowledge of the environment that can be flexibly accessed (e.g., for reasoning or recall) (Cohen and Eichenbaum, 1994; Eichenbaum, 2004). How this type of hippocampal representation for structural forms (i.e., hierarchies) accrued across multiple experiences is compatible with a key role for the hippocampus in supporting memory for individual episodes constitutes an important area for future research (Eichenbaum, 2004; Kumaran and McClelland, 2012; Zeithamova et al., 2012).

Our experimental design was specifically configured to address a key question that has not been explored in previous studies, namely, whether learning about a hierarchy of which one is a part recruits similar or distinct neural mechanisms than learning about a hierarchy that is composed entirely of other individuals. The difference had notable behavioral signatures, both in the congruency RT effect observed in the IAT and from information obtained at debriefing, which suggested that the participants found the scenario naturalistic. At a neural level, in contrast to the shared representations for hierarchies in the Self and Other condition in the amygdala and anterior hippocampus, we found signatures of MPFC activity that were selective to the Self condition, a finding that cannot be attributed to differences in performance, because this was comparable across conditions. Specifically, MPFC activity showed a robust correlation with the degree to which participants updated their knowledge of the hierarchy from trial to trial. The relevant area of the MPFC was the only region to show a significantly greater correlation between neural activity and this SMC model-derived hierarchy update signal in the Self condition than the Other condition (i.e., no significant difference was observed in the amygdala or hippocampus). Furthermore, we found evidence for a selective coupling between the MPFC and the amygdala and hippocampus during updating of hierarchy knowledge in the Self condition, consistent with the anatomical connectivity between these regions (Beckmann et al., 2009; Carmichael and Price, 1995). A nearby MPFC region also exhibited significantly greater correlation between neural activity and SMC model-estimated chosen power in the Self compared with the Other condition (i.e., in both training and test trials). Interestingly, this dissociation between the amygdala and anterior hippocampus and the MPFC was also evident under very different circumstances: when participants were performing an incidental categorization task (i.e., scanning phase 2: Figures 6 and 7). Even though information concerning the power of individuals was irrelevant in this setting, we observed specific coding of self-related information about power, and domain-general coding of power, in the MPFC and amygdala and anterior hippocampus, respectively.

Our results, therefore, align with several strands of research that have implicated a similar ventral region of the MPFC in the representation and processing of self-relevant information (Adolphs, 2009; Denny et al., 2012; Jenkins et al., 2008; Kelley et al., 2002; Kumaran and Maguire, 2005; Macrae et al., 2004; Mitchell et al., 2005, 2006; Mobbs et al., 2009; Ochsner et al., 2004; Tamir and Mitchell, 2011, 2012; Wittmann et al., 2016). This evidence has come from a range of studies: experiments in which participants are asked to judge the applicability of traits to themselves compared with others (Denny et al., 2012; Kelley et al., 2002; Mitchell et al., 2006), work suggesting that items subjected to self-related processing are afforded privileged status in memory (Macrae et al., 2004), and research on constructing imagined scenarios involving either oneself or others (De Brigard et al., 2015; FeldmanHall et al., 2012; Hassabis et al., 2014; Schacter and Addis, 2007). A natural constraint of this previous body of work (see Denny et al., 2012, for a meta-analysis), however, is that MPFC activity elicited in relation to self-attributions (e.g., trait judgments such as "am I trustworthy?"), compared with other-related judgments (e.g., "is Bill Clinton

trustworthy?"), could reflect either differences in prior knowledge about oneself or instead unique aspects of self-related representation and processing. Critically, our experimental design allowed us to effectively isolate the learning and representation of self-related information from prior knowledge in two ways. First, the power of individuals in the Self and Other hierarchies was arbitrary and needed to be learned "from scratch," rendering prior knowledge concerning oneself irrelevant. Second, our paradigm allowed us to demonstrate that the MPFC effects reported were robust to the exclusion of trials involving the "you" and "him or her" profile pictures (i.e., trials in which the participant or his or her friend was directly involved, such as the P5 versus P6 training trial), providing evidence that the MPFC represents the power of individuals in one's own hierarchy, a dimension that is known to exert significant behavioral influences (Chang et al., 2011; Cheney and Seyfarth, 1990). Hence, our results provide compelling evidence that neural mechanisms operating in the MPFC are distinctive with respect to self-related information and accordingly suggest that self-relevant information may indeed be uniquely represented in the brain, with consequent implications for the regulation of cooperative and competitive interactions (Apps et al., 2016; Crockett et al., 2014; Seyfarth and Cheney, 2012).

It is interesting to relate our work to evidence suggesting that the anterior cingulate cortex gyrus (ACCg), a region that contains Brodmann areas 24 a/b and 32 (Apps et al., 2016) and whose anterior portion overlaps with the MPFC region identified in our study, makes an important contribution to social cognition (Apps et al., 2016; Rudebeck, 2006). An emerging perspective suggests that the ACCg plays a key role in facilitating cooperative and competitive interactions, by tracking parameters such as the value and cost of options to others (Apps et al., 2016; Chang et al., 2013). As such, neuronal activity in ACCg has been referred to as situated in an Other-centric reference frame, with neurons in this region coding specifically for reward receipt by the other individual in a dictator game, contrasting with the self-centric coding (i.e., for reward receipt by self) of neurons in other regions such as the orbitofrontal cortex (Chang et al., 2013). On the face of it, evidence for other-referenced coding in the ACCg runs contrary to our MPFC findings; in fact, our results are entirely consistent with this perspective. Specifically, we show that the MPFC supports the learning and representation of power information about other individuals, when these individuals are part of our group rather than another group (i.e., Self > Other). As noted previously, our findings were robust to the exclusion of trials in which participants themselves were involved. As such, our results suggest that the ACCg/MPFC tracks the motivation of others through representations that not only code for the values and costs of reward to other individuals (Apps et al., 2016), but also incorporates rank information, particularly within one's own social group, dovetailing with behavioral evidence concerning the influence of social dominance and familiarity on cooperative behavior (Chang et al., 2011; Molenberghs, 2013; Seyfarth and Cheney, 2012).

Our results, however, do provide an apparent contrast with recent work arguing that MPFC representations do not inherently distinguish between self-related and other-related information (i.e., are "agent independent") (Garvert et al., 2015; Nicolle et al., 2012). Specifically, one study (Nicolle et al., 2012) argued that different MPFC regions may represent value in the context of a temporal discounting task as a function of whether this information is directly relevant to the choice to be executed or not, rather than whether it is self relevant or other relevant. Several factors may account for this discrepancy: one key difference is that in our study, the power of individuals in both the Self and Other hierarchies was arbitrary and needed to be learned through experience. Hence in our study, the emphasis was on learning new self-relevant information rather than on simulation, whereby another's preferences could be simulated using one's own preexisting preferences as an anchor (Garvert et al., 2015). Such a simulation would have been futile in our paradigm, because the power of individuals in the Other hierarchy could not be assessed using oneself as a template. One hypothesis, therefore, is that the learning and representation of recently experienced self-related information is subserved by a distinct ventral part of the MPFC, which if appropriate can be leveraged to simulate the preferences and behavior of others, a notion that aligns with the proposal that the simulation of other individuals similar to oneself recruits this region of MPFC (Jenkins et al., 2008; Mitchell et al., 2006).

Conclusions

Linear hierarchies and related structures (e.g., trees) are pervasive throughout the social domain but are also of more general importance in organizing information in an efficient way to facilitate inductive inferences (Kemp and Tenenbaum, 2008). Our study reveals neural computations by which observations of pairwise "contests" are used to update estimates of individuals' power within a hierarchy and provides compelling evidence that a Bayesian inference scheme, which has certain parallels with the Trueskill ratings system (Herbrich et al., 2006) used in large-scale multiplayer games, underlies this process, rather than a simpler RL mechanism. At the same time, our results, in ascribing a specific role to the MPFC in the learning of one's own social hierarchy under tightly controlled experimental conditions, invigorate the debate concerning whether self-relevant information is indeed afforded a unique representational status in the brain.

EXPERIMENTAL PROCEDURES

See Supplemental Experimental Procedures for a full description of task, computational models, and fMRI analysis procedures.

Phase 1: Learn, Scanned

In this phase of the experiment, participants acquired knowledge about the Self and Other hierarchies in parallel, with blocks of Self trials alternating with blocks of Other trials and training trial blocks alternating with test trial blocks (see Figure 1 and Supplemental Experimental Procedures for details of trial schedule).

Phase 2: Categorization, Scanned

In this phase, participants were presented with individual face pictures from the Self and Other hierarchies (excluding the profile pictures depicting themselves in the Self condition or their friends in the Other condition) while performing an incidental categorization judgement (see Figure 6 and Supplemental Experimental Procedures).

SUPPLEMENTAL INFORMATION

Supplemental Information includes Supplemental Experimental Procedures, three figures, and eight tables and can be found with this article online at http://dx.doi.org/10.1016/j.neuron.2016.10.052.

AUTHOR CONTRIBUTIONS

D.K., A.B., and D.H. designed the study. A.B. and D.K. collected the data. D.K., C.B., and P.D. formulated the computational models. D.K. performed the model fitting. D.K. and A.B. analyzed the data. D.K. and P.D. wrote the paper together with C.B., D.H., and A.B. A.B. and C.B. contributed equally to the study.

ACKNOWLEDGMENTS

We thank Chris Summerfield and Benedetto de Martino for comments on a previous version of the manuscript. This work was funded by a Wellcome Intermediate Fellowship (grant number is WT085189MA) to D.K.

Received: July 10, 2016
Revised: September 17, 2016
Accepted: October 21, 2016
Published: December 7, 2016

REFERENCES

Adolphs, R. (2009). The social brain: neural basis of social knowledge. Annu. Rev. Psychol. *60*, 693–716.

Amodio, D.M., and Frith, C.D. (2006). Meeting of minds: the medial frontal cortex and social cognition. Nat. Rev. Neurosci. *7*, 268–277.

Apps, M.A.J., Rushworth, M.F.S., and Chang, S.W.C. (2016). The anterior cingulate gyrus and social cognition: tracking the motivation of others. Neuron *90*, 692–707.

Ashby, F.G., and Waldschmidt, J.G. (2008). Fitting computational models to fMRI. Behav. Res. Methods *40*, 713–721.

Beckmann, M., Johansen-Berg, H., and Rushworth, M.F.S. (2009). Connectivity-based parcellation of human cingulate cortex and its relation to functional specialization. J. Neurosci. *29*, 1175–1190.

Behrens, T.E.J., Hunt, L.T., and Rushworth, M.F.S. (2009). The computation of social behavior. Science *324*, 1160–1164.

Burke, C.J., Tobler, P.N., Baddeley, M., and Schultz, W. (2010). Neural mechanisms of observational learning. Proc. Natl. Acad. Sci. U S A *107*, 14431–14436.

Byrne, R.W., and Bates, L.A. (2010). Primate social cognition: uniquely primate, uniquely social, or just unique? Neuron *65*, 815–830.

Carmichael, S.T., and Price, J.L. (1995). Limbic connections of the orbital and medial prefrontal cortex in macaque monkeys. J. Comp. Neurol. *363*, 615–641.

Chang, S.W.C., Winecoff, A.A., and Platt, M.L. (2011). Vicarious reinforcement in rhesus macaques (macaca mulatta). Front. Neurosci. *5*, 27.

Chang, S.W.C., Gariépy, J.-F., and Platt, M.L. (2013). Neuronal reference frames for social decisions in primate frontal cortex. Nat. Neurosci. *16*, 243–250.

Cheney, D.L., and Seyfarth, R.M. (1990). How Monkeys See the World: Inside the Mind of Another Species (University of Chicago Press).

Chiao, J.Y., Adams, R.B., Tse, P.U., Lowenthal, L., Richeson, J.A., and Ambady, N. (2008). Knowing who's boss: fMRI and ERP investigations of social dominance perception. Group Process. Intergroup Relat. *11*, 201–214.

Cohen, N.J., and Eichenbaum, H.B. (1994). Memory, Amnesia and the Hippocampal System (MIT Press).

Crockett, M.J., Kurth-Nelson, Z., Siegel, J.Z., Dayan, P., and Dolan, R.J. (2014). Harm to others outweighs harm to self in moral decision making. Proc. Natl. Acad. Sci. U S A *111*, 17320–17325.

Daw, N.D. (2011). Trial-by-trial data analysis using computational models. Decis. Making Affect. Learn. Atten. Perform. *23*, 555.

De Brigard, F., Nathan Spreng, R., Mitchell, J.P., and Schacter, D.L. (2015). Neural activity associated with self, other, and object-based counterfactual thinking. Neuroimage *109*, 12–26.

Denny, B.T., Kober, H., Wager, T.D., and Ochsner, K.N. (2012). A meta-analysis of functional neuroimaging studies of self- and other judgments reveals a spatial gradient for mentalizing in medial prefrontal cortex. J. Cogn. Neurosci. *24*, 1742–1752.

Doucet, A., Godsill, S., and Andrieu, C. (2000). On sequential Monte Carlo sampling methods for Bayesian filtering. Stat. Comput. *10*, 197–208.

Eichenbaum, H. (2004). Hippocampus: cognitive processes and neural representations that underlie declarative memory. Neuron *44*, 109–120.

Engell, A.D., Haxby, J.V., and Todorov, A. (2007). Implicit trustworthiness decisions: automatic coding of face properties in the human amygdala. J. Cogn. Neurosci. *19*, 1508–1519.

Fanselow, M.S., and Dong, H.-W. (2010). Are the dorsal and ventral hippocampus functionally distinct structures? Neuron *65*, 7–19.

FeldmanHall, O., Dalgleish, T., Thompson, R., Evans, D., Schweizer, S., and Mobbs, D. (2012). Differential neural circuitry and self-interest in real vs hypothetical moral decisions. Soc. Cogn. Affect. Neurosci. *7*, 743–751.

Frank, M.J., Rudy, J.W., and O'Reilly, R.C. (2003). Transitivity, flexibility, conjunctive representations, and the hippocampus. II. A computational analysis. Hippocampus *13*, 341–354.

Friston, K.J., Buechel, C., Fink, G.R., Morris, J., Rolls, E., and Dolan, R.J. (1997). Psychophysiological and modulatory interactions in neuroimaging. Neuroimage *6*, 218–229.

Garvert, M.M., Moutoussis, M., Kurth-Nelson, Z., Behrens, T.E.J., and Dolan, R.J. (2015). Learning-induced plasticity in medial prefrontal cortex predicts preference malleability. Neuron *85*, 418–428.

Gershman, S.J. (2015). A unifying probabilistic view of associative learning. PLoS Comput. Biol. *11*, e1004567.

Greenwald, A.G., McGhee, D.E., and Schwartz, J.L. (1998). Measuring individual differences in implicit cognition: the implicit association test. J. Pers. Soc. Psychol. *74*, 1464–1480.

Grosenick, L., Clement, T.S., and Fernald, R.D. (2007). Fish can infer social rank by observation alone. Nature *445*, 429–432.

Hackel, L.M., Doll, B.B., and Amodio, D.M. (2015). Instrumental learning of traits versus rewards: dissociable neural correlates and effects on choice. Nat. Neurosci. *18*, 1233–1235.

Hampton, A.N., Bossaerts, P., and O'Doherty, J.P. (2008). Neural correlates of mentalizing-related computations during strategic interactions in humans. Proc. Natl. Acad. Sci. U S A *105*, 6741–6746.

Hassabis, D., Spreng, R.N., Rusu, A.A., Robbins, C.A., Mar, R.A., and Schacter, D.L. (2014). Imagine all the people: how the brain creates and uses personality models to predict behavior. Cereb. Cortex *24*, 1979–1987.

Herbrich, R., Minka, T., and Graepel, T. (2006). Trueskill™: A Bayesian skill rating system. In Advances in Neural Information Processing Systems: Proceedings of the First 12 Conferences, M.I. Jordan, Y. LeCun, and S.A. Solla, eds. (MIT Press), pp. 569–576.

Jenkins, A.C., Macrae, C.N., and Mitchell, J.P. (2008). Repetition suppression of ventromedial prefrontal activity during judgments of self and others. Proc. Natl. Acad. Sci. U S A *105*, 4507–4512.

Kelley, W.M., Macrae, C.N., Wyland, C.L., Caglar, S., Inati, S., and Heatherton, T.F. (2002). Finding the self? An event-related fMRI study. J. Cogn. Neurosci. *14*, 785–794.

Kemp, C., and Tenenbaum, J.B. (2008). The discovery of structural form. Proc. Natl. Acad. Sci. U S A *105*, 10687–10692.

King-Casas, B. (2005). Getting to know you: reputation and trust in a two-person economic exchange. Science *308*, 78–83.

Kumaran, D., and Maguire, E.A. (2005). The human hippocampus: cognitive maps or relational memory? J. Neurosci. *25*, 7254–7259.

Kumaran, D., and McClelland, J.L. (2012). Generalization through the recurrent interaction of episodic memories: a model of the hippocampal system. Psychol. Rev. *119*, 573–616.

Kumaran, D., Melo, H.L., and Duzel, E. (2012). The emergence and representation of knowledge about social and nonsocial hierarchies. Neuron *76*, 653–666.

Lebreton, M., Jorge, S., Michel, V., Thirion, B., and Pessiglione, M. (2009). An automatic valuation system in the human brain: evidence from functional neuroimaging. Neuron *64*, 431–439.

Macrae, C.N., Moran, J.M., Heatherton, T.F., Banfield, J.F., and Kelley, W.M. (2004). Medial prefrontal activity predicts memory for self. Cereb. Cortex *14*, 647–654.

Marsh, A.A., Blair, K.S., Jones, M.M., Soliman, N., and Blair, R.J.R. (2004). Dominance and submission: the ventrolateral prefrontal cortex and responses to status cues. J. Cogn. Neurosci. *21*, 713–724.

Mitchell, J.P., Banaji, M.R., and Macrae, C.N. (2005). The link between social cognition and self-referential thought in the medial prefrontal cortex. J. Cogn. Neurosci. *17*, 1306–1315.

Mitchell, J.P., Macrae, C.N., and Banaji, M.R. (2006). Dissociable medial prefrontal contributions to judgments of similar and dissimilar others. Neuron *50*, 655–663.

Mobbs, D., Yu, R., Meyer, M., Passamonti, L., Seymour, B., Calder, A.J., Schweizer, S., Frith, C.D., and Dalgleish, T. (2009). A key role for similarity in vicarious reward. Science *324*, 900.

Molenberghs, P. (2013). The neuroscience of in-group bias. Neurosci. Biobehav. Rev. *37*, 1530–1536.

Nicolle, A., Klein-Flügge, M.C., Hunt, L.T., Vlaev, I., Dolan, R.J., and Behrens, T.E. (2012). An agent independent axis for executed and modeled choice in medial prefrontal cortex. Neuron *75*, 1114–1121.

Niv, Y., Joel, D., and Dayan, P. (2006). A normative perspective on motivation. Trends Cogn. Sci. *10*, 375–381.

Niv, Y., Daniel, R., Geana, A., Gershman, S.J., Leong, Y.C., Radulescu, A., and Wilson, R.C. (2015). Reinforcement learning in multidimensional environments relies on attention mechanisms. J. Neurosci. *35*, 8145–8157.

Noonan, M.P., Sallet, J., Mars, R.B., Neubert, F.X., O'Reilly, J.X., Andersson, J.L., Mitchell, A.S., Bell, A.H., Miller, K.L., and Rushworth, M.F. (2014). A neural circuit covarying with social hierarchy in macaques. PLoS Biol. *12*, e1001940.

O'Reilly, J.X., Woolrich, M.W., Behrens, T.E.J., Smith, S.M., and Johansen-Berg, H. (2012). Tools of the trade: psychophysiological interactions and functional connectivity. Soc. Cogn. Affect. Neurosci. *7*, 604–609.

Ochsner, K.N., Knierim, K., Ludlow, D.H., Hanelin, J., Ramachandran, T., Glover, G., and Mackey, S.C. (2004). Reflecting upon feelings: an fMRI study of neural systems supporting the attribution of emotion to self and other. J. Cogn. Neurosci. *16*, 1746–1772.

Paz-Y-Miño C, G., Bond, A.B., Kamil, A.C., and Balda, R.P. (2004). Pinyon jays use transitive inference to predict social dominance. Nature *430*, 778–781.

Raftery, A.E. (1995). Bayesian model selection in social research. Sociol. Methodol. *25*, 111–163.

Rudebeck, P.H. (2006). A role for the macaque anterior cingulate gyrus in social valuation. Science *313*, 1310–1312.

Rushworth, M.F.S., Mars, R.B., and Sallet, J. (2013). Are there specialized circuits for social cognition and are they unique to humans? Curr. Opin. Neurobiol. *23*, 436–442.

Schacter, D.L., and Addis, D.R. (2007). The cognitive neuroscience of constructive memory: remembering the past and imagining the future. Philos. Trans. R. Soc. B Biol. Sci. *362*, 773–786.

Schultz, W., Dayan, P., and Montague, P.R. (1997). A neural substrate of prediction and reward. Science *275*, 1593–1599.

Seyfarth, R.M., and Cheney, D.L. (2012). The evolutionary origins of friendship. Annu. Rev. Psychol. *63*, 153–177.

Suzuki, S., Harasawa, N., Ueno, K., Gardner, J.L., Ichinohe, N., Haruno, M., Cheng, K., and Nakahara, H. (2012). Learning to simulate others' decisions. Neuron *74*, 1125–1137.

Tamir, D.I., and Mitchell, J.P. (2011). The default network distinguishes construals of proximal versus distal events. J. Cogn. Neurosci. *23*, 2945–2955.

Tamir, D.I., and Mitchell, J.P. (2012). Disclosing information about the self is intrinsically rewarding. Proc. Natl. Acad. Sci. U S A *109*, 8038–8043.

Tervo, D.G.R., Tenenbaum, J.B., and Gershman, S.J. (2016). Toward the neural implementation of structure learning. Curr. Opin. Neurobiol. *37*, 99–105.

Todorov, A., Said, C.P., Engell, A.D., and Oosterhof, N.N. (2008). Understanding evaluation of faces on social dimensions. Trends Cogn. Sci. *12*, 455–460.

Todorov, A., Said, C.P., Oosterhof, N.N., and Engell, A.D. (2011). Task-invariant brain responses to the social value of faces. J. Cogn. Neurosci. *23*, 2766–2781.

Volz, K.G., Kessler, T., and von Cramon, D.Y. (2009). In-group as part of the self: in-group favoritism is mediated by medial prefrontal cortex activation. Soc. Neurosci. *4*, 244–260.

von Fersen, L., Wynne, C.D., Delius, J.D., and Staddon, J.E. (1991). Transitive inference formation in pigeons. J. Exp. Psychol. Anim. Behav. Process. *17*, 334–341.

Watkins, C.J.C.H., and Dayan, P. (1992). Q-learning. Mach. Learn. *8*, 279–292.

Whalen, P.J. (1998). Fear, vigilance, and ambiguity: initial neuroimaging studies of the human amygdala. Curr. Dir. Psychol. Sci. *7*, 177–188.

Wilson, R.C., and Niv, Y. (2015). Is model fitting necessary for model-based fMRI? PLoS Comput. Biol. *11*, e1004237.

Wimmer, G.E., Daw, N.D., and Shohamy, D. (2012). Generalization of value in reinforcement learning by humans. Eur. J. Neurosci. *35*, 1092–1104.

Winston, J.S., Strange, B.A., O'Doherty, J., and Dolan, R.J. (2002). Automatic and intentional brain responses during evaluation of trustworthiness of faces. Nat Neurosci. *5*, 277–283.

Wittmann, M.K., Kolling, N., Faber, N.S., Scholl, J., Nelissen, N., and Rushworth, M.F. (2016). Self-other mergence in the frontal cortex during cooperation and competition. Neuron *91*, 482–493.

Yliniemi, L., and Tumer, K. (2013). ELO ratings for structural credit assignment in multiagent systems. In AAAI Workshop—Technical Report, pp. 152–154.

Zeithamova, D., Schlichting, M.L., and Preston, A.R. (2012). The hippocampus and inferential reasoning: building memories to navigate future decisions. Front. Hum. Neurosci. *6*, 70.

Zink, C.F., Tong, Y., Chen, Q., Bassett, D.S., Stein, J.L., and Meyer-Lindenberg, A. (2008). Know your place: neural processing of social hierarchy in humans. Neuron *58*, 273–283.

Neuron, Volume *92*

Supplemental Information

Computations Underlying Social Hierarchy Learning: Distinct Neural Mechanisms for Updating and Representing Self-Relevant Information

Dharshan Kumaran, Andrea Banino, Charles Blundell, Demis Hassabis, and Peter Dayan

SUPPLEMENTAL INFORMATION.

Computations underlying Social Hierarchy Learning: Distinct Neural Mechanisms for Updating and Representing Self-Relevant Information

Dharshan Kumaran[1,3], Andrea Banino[1], Charles Blundell[1], Demis Hassabis[1,2], Peter Dayan[2]

[1]*Google DeepMind, 5 New Street Square, London EC4A 3TW.*

[2]*Gatsby Computational Neuroscience Unit, 25 Howland St, London W1T 4JG, UK.*

[3]*Institute of Cognitive Neuroscience, University College London, 17 Queen Square, WC1N 3AR, UK*

Contains:

3 Supplemental Figures

8 Supplemental Tables

Supplemental Experimental Procedures

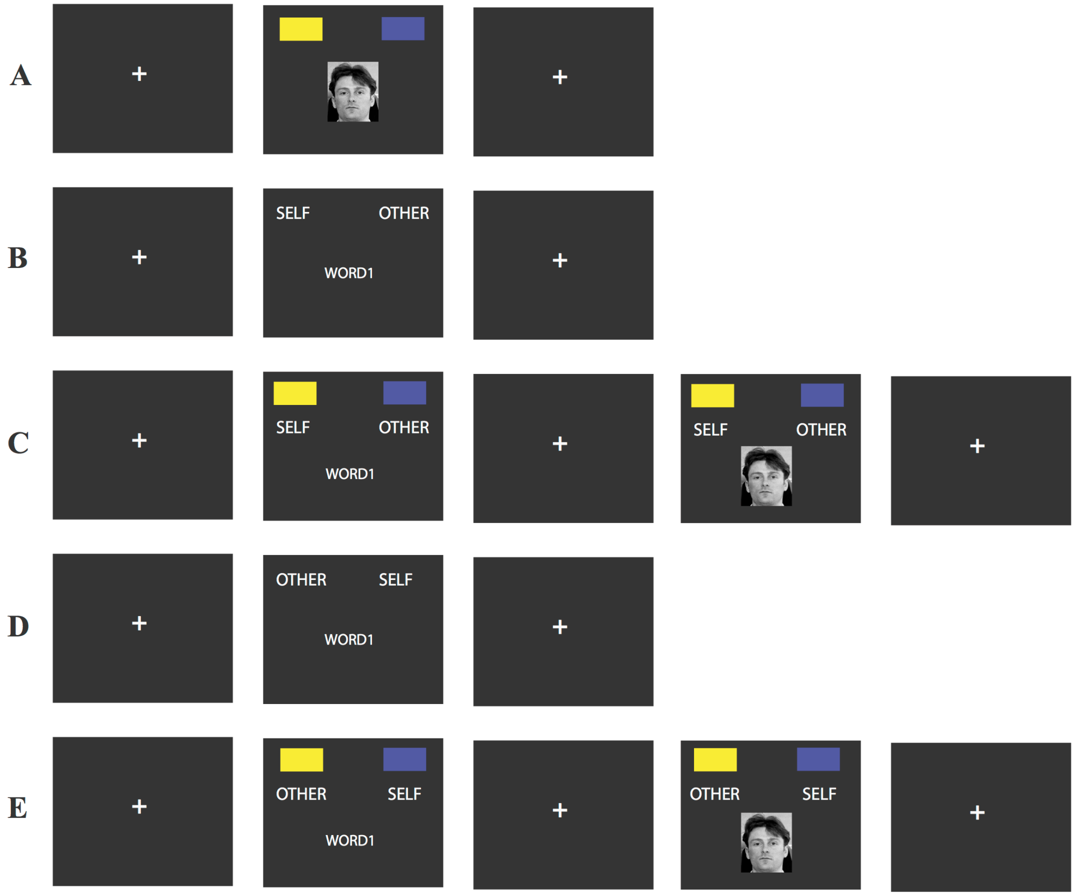

Supplemental Figure 1 (Related to Fig 2). Full Implicit Association Test (IAT) experimental protocol. Example of a sequence of IAT shown. The first step **(A)** introduced the target-concept discrimination. The second step **(B)** introduced the attribute discrimination. In the third step **(C)** congruent trials are presented. In the fourth step **(D)**, participants learned reversal attribute discrimination. In the fifth block **(E)** incongruent trials are presented. The fact of whether participants performed the congruent trials or the incongruent trials first was counterbalanced across participants. See Supplemental Experimental Procedures for details.

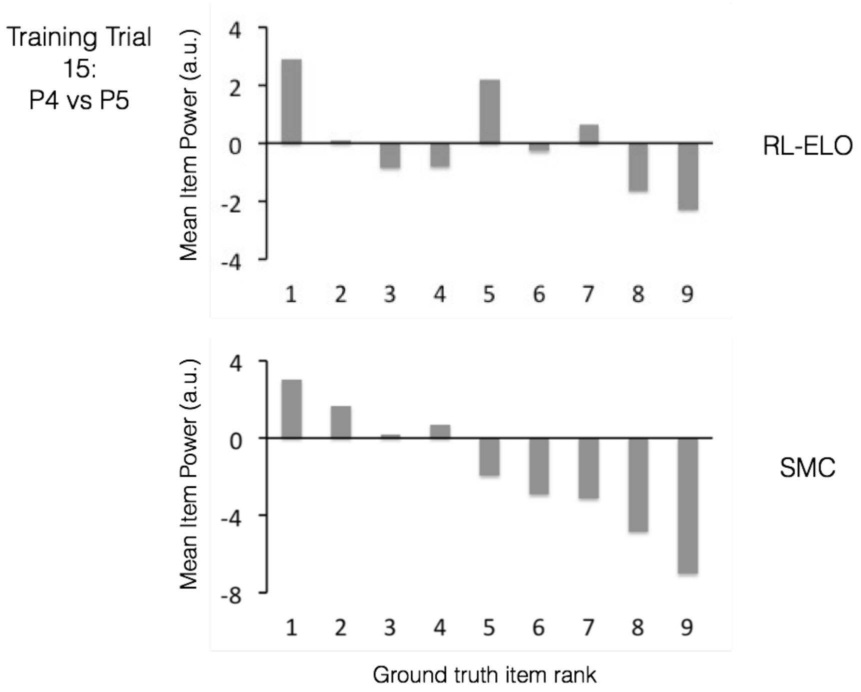

Figure S2A (Related to Fig 4): **Illustrative subject**, mean item powers shown at training trial 15 (i.e. first training trial block). For this case study we describe a high performing subject (average correct overall 92% on training/test trials) who showed a large difference in model fit between the SMC and RL-ELO models: negative log likelihood (NLL) for first ½ of experiment was 53 vs 75 respectively, and for second ½ experiment 23.6 vs 22.6, respectively. We focus on the first block of 16 training trials in the Self condition, where each of the 8 training pairs (e.g. P1 vs P2…P8 vs P9) occurs twice in pseudorandom order (different across subjects). We consider trial 15, which involves items P4 and P5, and the subject responds correctly (i.e. chooses P4). Due to the trial history, there is a transient imbalance in reinforcement associated with the 2 items: item P4 has been the winning item in 1 trial, P5 in 2 trials (i.e. imbalance = -1). The RL-ELO model, with parameters best fit to this subject/condition, is associated with a large NLL due to the imbalance in reinforcement (i.e. NLL for trial 15 =3.1). In contrast, the SMC NLL is 0.1. The mean power of each item at this point (i.e. prior to feedback on trial 15) is shown for the RL-ELO and SMC models (see Fig S2A): the SMC model mean item power already reflect the hierarchy largely correctly, whilst the RL-ELO item powers do not. Note that the SMC model is much less but not entirely insensitive to reinforcement imbalance (see below for systematic analysis): the incorrect ordering of item 3 reflects that this item was the winner in only one previous (P3 vs P4) trial, and was the loser in 2 trials (i.e. P2 vs P3).

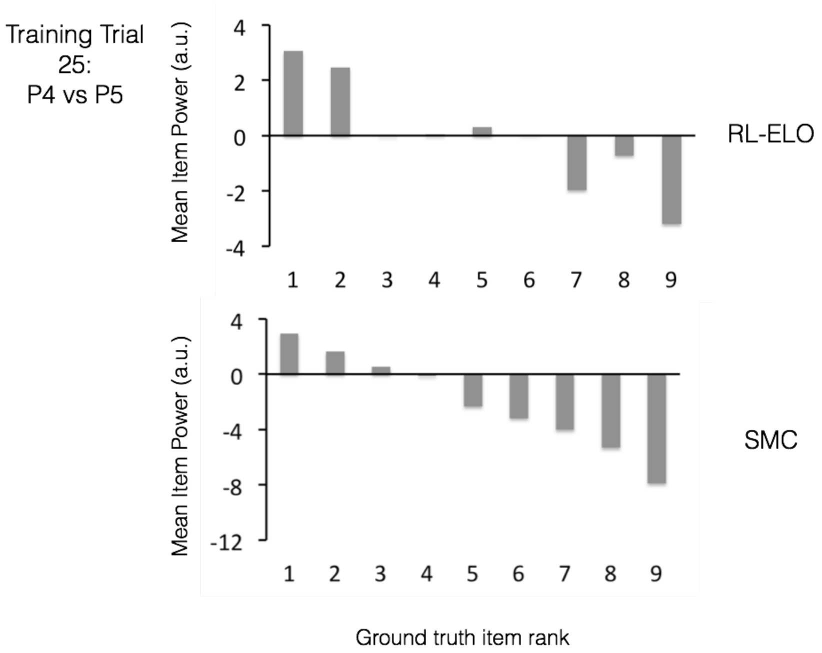

Figure S2B (Related to Fig 4): **Illustrative subject**, mean item powers shown at training trial 25 (i.e. second training trial block). This demonstrates the superior ability of the SMC model to converge on the true rank ordering with less experience. We next carried out a ***systematic analysis*** to assess the differential sensitivity of the SMC and RL-ELO models to transient imbalances in reinforcement history of individual items. To do this, we used the trial orders generated for the actual subjects and focussed on training trials where the reinforcement associated with each item is equated within each block: i.e. training pairs P2 vs P3, P3 vs P4, …., P7 vs P8 (i.e excluding the outer training pairs P1 vs P2 and P8 vs P9 since P1 is always positively reinforced and P9 negatively reinforced). We focussed on the first half of the experiment, and determined the set of parameters that best fit synthetic choice data consisting of all correct responses. We discarded the first set of 7 training trials since the correct response is unknowable at this stage (i.e. even by an "optimal" model). The SMC model NLL was 39.3 (average across Self and Other conditions), whilst the RL-ELO model NLL was 77.2. This large difference in NLL between the SMC and RL-ELO models in fitting this synthetic dataset demonstrates the superior ability of the SMC model to converge on the true rank ordering with less experience (e.g. by trial 25 in the illustrated subject in figure above). We then ran separate linear regression models to quantify the relationship between trial-by-trial reinforcement imbalance (independent variable) and NLL (dependent variable), for the SMC and RL models – where the data was combined across all simulated subjects, with trial type entered as additional independent variable. We ran separate regression analyses for the first and second quarters of the experiment. We found a significant effect of imbalance on NLL for the RL model in the first quarter of the experiment (slope coefficient $b=-0.24$, standard error (se) = 0.0076, $p<0.001$; adj-$R2 = 0.47$) – that was similar for the second quarter ($b=-0.197$, se 0.0072, $p<0.001$ adj-$R2=0.40$). We found a significant but much smaller effect of imbalance in the SMC model in the first quarter ($b=-0.073$, se = 0.0099, $p<0.001$; adj $R2 = 0.046$;) and second quarter ($b=-0.050$, se 0.003, $p<0.001$; adj $R2 = 0.041$). The RL model was associated with a significantly greater effect of imbalance on NLL: ($Z=24.8$, $p<0.0001$; across both quarters). Further, there was a significantly greater effect of imbalance of the SMC model in the first quarter as compared to the second quarter ($Z=2.5$ $p<0.01$). The SMC and RL-ELO models, therefore, have qualitative different updating mechanisms: during a given training trial, the SMC model updates its posterior distribution over item values based on all the available experience – rather than basing its update solely on the current value of the items presented in the trial as in the RL-ELO model. This difference leads the RL-ELO model to be much more sensitive to transient imbalances in the reinforcement history of individual items, as compared to the SMC model – which shows a small sensitivity to reinforcement imbalance that decreased with increasing experience. The difference in updating mechanism results in the SMC model being able to more

rapidly converge on the correct rank ordering: indeed, the difference between model fits (NLL) showed a highly significant correlation with subjects' performance, with high performing subject showing the greatest advantage for the SMC model (pearson's correlation: r = -0.75, -0.79; p<0.0001 for both self and other conditions). Notably, the vast majority of subjects were better fit by the SMC model (22 and 23 out of 28 subjects in Self/Other condition).

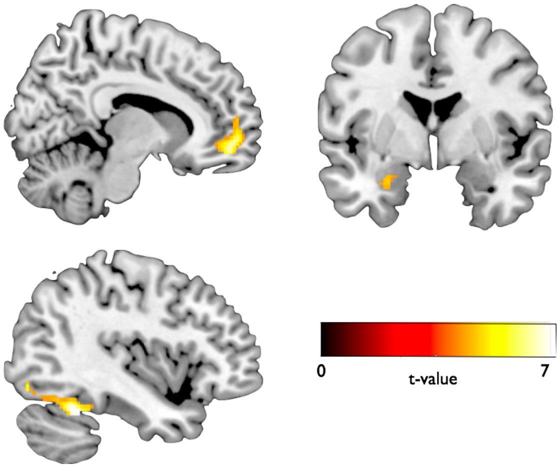

Figure S3A (Related to Fig 5)**:** Learn Phase: Brain regions where activity negatively correlates with SMC-modelled entropy during training trials (Main effect: Self and Other). In an analysis where RT was included in the GLM (correlation with entropy regressors ~ 0.25 across subjects), we found significant negative correlations with the entropy (i.e relating to participants' estimates of the probability of each item winning against the other) in the left amygdala/anterior hippocampus as well as FFA-proximate area and vMPFC. Whole brain analysis: significant correlation between activity in vmPFC (top left), amygdala (top right), FFA-proximate region evident (bottom left) (see Table S5A). Display p<0.005 corrected.

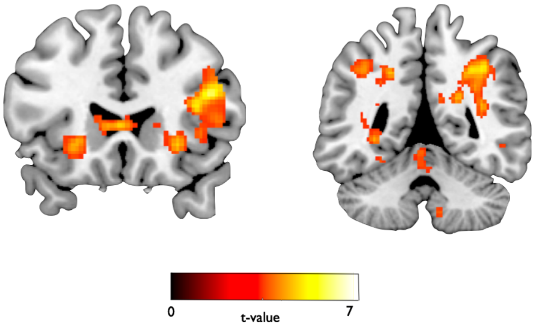

Figure S3B (Related to Fig 5)**:** Learn Phase: Brain regions where activity positively correlates with SMC-modelled entropy during training trials (Main effect: Self and Other). Positive correlations with entropy were found in the dorsolateral prefrontal cortex, insula, medial parietal cortex and intraparietal sulcus. Whole brain analysis: significant correlation between activity in DLPFC (left panel), insula (left panel), intraparietal sulcus (right panel) evident (see Table S5B). Display p<0.005 uncorrected.

Table S1 (Related to Fig 1)**:** Brain areas whose activity significantly correlated with the SMC modelled difference in power during test trials: Main effect across Self and Other conditions

Region	*x*	*y*	*z*	*z-score*	
Amygdala/Anterior HC	-18	-2	-24	5.44	FWE_p p=0.003
Ventromedial PFC	4	60	-10	5.10	FWE_p p=0.013
Posterior Cingulate	0	-32	36	4.45	FWE_c p=0.017
Fusiform	-40	-50	-22	5.78	FWE_p p=0.003
Orbitofrontal	-32	36	-14	4.13	p<0.001 unc

HC = hippocampus, PFC = prefrontal cortex. FWE_p is whole brain FWE corrected at peak-level, FWE_c is whole brain FWE corrected at cluster level (cluster threshold: p=0.001), SVC is small volume corrected (see Supplemental Experimental Procedures for details).

Table S2 (Related to Fig 4)**:** Results of Neural Model Comparison, Relative BIC differences between SMC and RL-ELO models.

**L AMY**	**R AMY**	**L HC**	**R HC**	**MPFC**	**FFA**	**vMPFC**
**41.6***	**38.4**	**46.4***	**43.8**	**32.0****	**17.0***	**3.2**

Numbers reflect relative BIC differences between models (i.e. BIC_RL-ELO – BIC_SMC). Values are summed across participants. Single set of parameters for each model across subjects (i.e. best fit to behavioural data). AMY = amygdala, HC = hippocampus, MPFC and vMFPC are functionally defined regions of medial and ventromedial prefrontal cortex; FFA is FFA-proximate region as referred to in main text, functionally defined (see Supplemental Experimental Procedures). Following previous work (Niv et al., 2015) we also report significance of the difference between the log likelihoods of SMC and RL-ELO, using permutation testing. *significant at p<0.05; ** significant at p<0.01. R HC p=0.068; vmPFC p>0.3.

Table S3A (Related to Fig 4)**:** Brain areas whose activity significantly correlated with the SMC modelled hierarchy update index during training trials: Main effect across Self and Other conditions.

Region	*x*	*y*	*z*	*z-score*	
MPFC	-8	44	10	5.04	FWE_p p=0.022
Hippocampus	-26	-22	-14	3.45	SVC p=0.041
Hippocampus	30	-36	-2	4.51	SVC p = 0.003
Orbitofrontal	30	36	-8	3.61	p<0.001 uncorr
Insula	-28	16	12	3.91	p<0.001 uncorr
Fusiform	-42	-50	-18	4.63	FWEc p=0.014

Table S3B (Related to Fig 4)**:** Brain areas whose activity significantly correlated with the SMC modelled hierarchy update index in Self condition during training trials.

Region	*x*	*y*	*z*	*z-score*	
MPFC	-6	46	12	4.22	SVC p=0.0040
Hippocampus	-24	-12	-16	3.32	SVC p=0.05
Posterior Cingulate	-6	-44	34	4.51	p<0.001 uncorr
Orbitofrontal	38	44	-14	4.21	p<0.001 uncorr

Table S4 (Related to Fig 4)**:** Brain areas whose activity shows significantly greater coupling with functionally defined MPFC seed region during updating of hierarchy knowledge in the Self, as compared to Other, condition.

Region	*x*	*y*	*z*	*z-score*	
Hippocampus	-26	-24	-14	3.25	SVC p=0.042
Amygdala	-18	0	-22	3.04	SVC p=0.040
Orbitofrontal	34	32	-16	3.62	p<0.001 uncorr
Orbitofrontal	-24	38	-8	3.25	p<0.001 uncorr

Table S5A (Related to Fig 5)**:** Brain areas whose activity showed a negative correlation with entropy over item pairs (i.e. probability of item being correct) during training trials: Main effect Self and Other.

Region	*x*	*y*	*z*	*z-score*	
Amygdala/anterior HC	-22	-8	-24	3.51	SVC p=0.032
Amygdala/anterior HC	24	-12	-26	3.42	p= 0.001 uncorr
Fusiform	-44	-52	-24	5.47	FWE_p p<0.003
vmPFC	14	50	-6	3.98	FWE_c p<0.002

Table S5B (Related to Fig 5)**:** Brain areas whose activity showed a positive correlation with entropy over item pairs (i.e. probability of item being correct) during training trials: Main effect Self and Other.

Region	*x*	*y*	*z*	*z-score*	
Dorsolateral PFC	44	22	22	4.65	FWE_c p<0.001
Insula	-26	24	-4	3.92	p<0.001 uncorr
Insula	26	20	-6	4.14	p<0.001 uncorr
Parietal cortex	2	-66	44	5.71	FWE_p p<0.001
Intraparietal sulcus	30	-54	46	5.12	FWE_p p<0.001

Table S6A (Related to Fig 5)**:** Brain areas whose activity significantly correlated with the SMC modelled chosen power during training trials: *main effect of Self and Other conditions*

Region	*x*	*y*	*z*	*z-score*	
vmPFC	-8	52	-12	3.74	SVC p=0.005
Hippocampus	16	-16	20	3.91	p<0.001 uncorr
Hippocampus	-24	-40	18	3.51	p<0.001 uncorr
Amygdala	-18	-4	20	3.13	p<0.001 uncorr
Striatum	14	2	-16	3.71	p<0.001 uncorr
Striatum	-22	10	-6	3.42	p<0.001 uncorr
Orbitofrontal	18	42	-8	3.91	p<0.001 uncorr

Table S6B (Related to Fig 5)**:** Brain areas whose activity significantly correlated with the SMC modelled chosen power during training trials in the *Self* condition

Region	*x*	*y*	*z*	*z-score*	
MPFC	4	44	2	3.01	SVC p=0.037
Striatum	16	8	-12	3.82	p<0.001 uncorr
Striatum	-8	10	-12	4.25	p<0.001 uncorr

Table S6C (Related to Fig 5)**:** Brain areas whose activity significantly correlated with the SMC modelled chosen power during training trials in the *Other* condition

Region	*x*	*y*	*z*	*z-score*	
vmPFC	-12	52	-10	3.86	SVC p=0.016

Table S6D (Related to Fig 5)**:** Brain areas whose activity significantly correlated with the SMC modelled chosen power during training trials: *Self* > *Other* condition

Region	*x*	*y*	*z*	*z-score*	
MPFC	6	42	4	4.23	SVC p=0.001

Table S7 (Related to Fig 6)**:** Brain areas whose activity showed a linear correlation with person rank in Categorization phase: Main effect Self and Other.

Region	*x*	*y*	*z*	*z-score*	
Amygdala	-32	-4	-22	3.72	SVC p=0.025
Hippocampus (anterior)	-32	-8	-20	3.64	SVC p=0.034

Scores	Mean	SD
**Power Explicit Scores:** Can you tell me – use your gut instinct – how powerful each of these individuals feels to you? [a]		
Top Ranked Individuals in the Self Hierarchy	9.07	1.60
Top Ranked Individuals in the Other Hierarchy	8.60	2.90
Bottom Ranked Individuals in the Self Hierarchy	2.28	1.99
Bottom Ranked Individuals in the Self Hierarchy	2.28	2.28
**Social Realism Scores** [a]		
**Top:** How realistic does it feel when you see "X" that he/she is the head of the company/top dog?		
Top Ranked Individuals in the Self Hierarchy	6.55	2.74
Top Ranked Individuals in the Other Hierarchy	6.63	2.43
**Bottom:** How realistic does it feel when you see "X" that he/she is the loser/lowest person in the company?		
Bottom Ranked Individuals in the Self Hierarchy	6.10	2.74
Bottom Ranked Individuals in the Self Hierarchy	6.52	2.43
**Explicit Identification Scores** [a]		
How much did you feel YOU were part of the company in the YOU trials?	5.57	2.34
How much did you feel your FRIEND was part of the company in the HIM/HER trials?	5.33	2.18
How much did you feel YOU were part of the company in the HIM/HER trials?	2.03	2.97
How much did you feel that you FRIEND was part of the company in the YOU trials?	0.83	1.53
**Friend Information** [b]		
How much do you like them?	9.57	0.72
How similar would you say they are to you in terms of overall perspective on life, personality, hobbies?	6.80	1.60

[a] on a scale from 0 (low) to 10 (high)

[b] on a scale from 1 (a little) to 10 (a lot)

Table S8 (Related to Fig 2)**: Results of debriefing session performed at end of experiment.** See Supplemental Experimental Procedures for details.

Supplemental Experimental Procedures.

Participants. Thirty healthy, right-handed individuals who were currently undertaking or had recently completed a university degree, participated in this experiment (age range 19-29; 18 female). Two of these participants performed at chance levels and were therefore excluded from the fMRI analyses. All participants gave informed written consent to participation in accordance with the local research ethics committee.

Stimuli: Faces. Face pictures were obtained from a widely used database (Stirling database: http://pics.stir.ac.uk): pictures are rendered in grayscale and depict male individuals sitting on a chair, with a neutral expression. Images were cropped below the chin line and resized, though hair was retained to preserve the naturalistic properties of the stimuli. Male participants learnt about hierarchies comprised of male individuals, and vice versa for female participants. To represent the participant themselves (i.e. Self condition) and a close friend (i.e. Other condition – see below for procedure for eliciting the friend), two profile pictures were created in Adobe Photoshop CS5. The profile pictures depicted a black silhouette, with the same background, size and colour of the other face pictures. Profile pictures incorporated the pronoun "You", and the pronoun "Him" or "Her" (i.e. for male/female participants, respectively: see Figure 1).

Hierarchies. Self and Other hierarchies were each comprised of 9 items (i.e. P1-P2-P3-P4-P5-P6-P7-P8-P9; where P=person and 1 is the highest ranking person and 9 the lowest ranking - Figure 1). The participant themself, denoted by the "You" profile picture – and their friend, denoted by "Him" or "Her" profile picture – always occupied the middle position in the hierarchy (i.e. P5). This was done to ensure that the rank of the participant and their friend was equated, and allowed us to create a controlled set of transitive inference test trials (see below). Apart from the profile pictures, the allocation of individual pictures to position in the hierarchy was randomized across the group of participants. No significant correlation was found between post-scanning ratings of attractiveness or dominance and rank order (ps > 0.1).

Prior to each scanning session, participants briefly performed a simple 1-back task in which they viewed each individual face three times – a procedure which is known to minimize stimulus novelty effects during scanning based on previous data (e.g. (Johnson et al., 2008)). Examples of faces used are shown in Figure 1.

Tasks and Procedures. Participants were first asked the name of a close friend of theirs that fulfilled two requirements: the friend had to be of the same sex and they had to have known them for more than three months. Participants were asked to imagine that they and their friend had recently joined two different companies. They were informed that the individuals within each company were distinct (i.e. no individual belonged to both companies), and that each company had a distinct coloured logo (i.e. either yellow or blue, assignment to Self or Other condition counterbalanced). As such, the coloured border (e.g. yellow) surrounding a face picture would indicate which company the individual belonged to (i.e. Self or Other). Notably, our experimental design incorporated a close friend in the Other condition – rather than an acquaintance or unfamiliar other individual – in order to render these conditions as similar as possible, thereby isolating the self/other dimension (e.g. see (Mitchell et al., 2006)).

They were informed that there would be two parts to the experiment: in the first phase ("Learn" phase) they would need to learn which individuals have more power within each company. In phase two ("Categorization" phase), they were told that they would need to use knowledge acquired during phase 1 to make judgements about individuals. Participants were told that they would be renumerated based on their performance in the Learn and Categorization phases. Our aim, therefore, was to develop a naturalistic experimental scenario in which subjects would develop knowledge of a social hierarchy that either involved themselves (i.e. Self condition) or a close friend (i.e. Other condition).

Phase 1 (Learn) In this phase of the experiment participants acquired knowledge about the Self and Other hierarchies in parallel.

Training trials (Figure 1). During a training trial, participants viewed adjacent individuals in each of the Self and Other hierarchies displayed on either side of the screen (i.e. 8 training pairs in Self and Other

conditions: i.e. P1 vs P2, P2 vs P3, P3 vs P4, P4 vs P5, P5 vs P6, P6 vs P7, P8 vs P9). The left-right position of an item on the screen was randomized across trials. They had 3 seconds in which to choose, via button press (i.e. left or right, index or middle finger of right hand respectively), the item which had "more power". After 3 seconds, a feedback screen appeared (2 second duration): this consisted of white circle below the chosen stimulus together with either "+20 points" or "-20 points", for a correct or incorrect response (in green/red color, respectively). A fixation cross of 1.5 seconds duration preceded the onset of the next trial. The renumeration received by participants for this phase of the experiment was determined directly from the number of points won.

Test trials (Figure 1B). During test trials, participants viewed pairs of non-adjacent individuals in the hierarchy (i.e. 8 inference pairs: P2 vs. P4, P2 vs. P5, P3 vs. P5, P3 vs. P6, P4 vs. P6, P4 vs. P7, P5 vs. P7, P5 vs. P8). Note that 4 of the inference pairs included the participant or their friend (e.g. P2 vs P5), and 4 did not (e.g. P3 vs P6). As in training trials, participants had 3 seconds in which to choose, via button press (i.e. left or right), the person which they thought had more power in either the hierarchy of which they were a part (i.e. Self condition), or that incorporated their friend (i.e. Other condition). Importantly, however, no feedback was presented during test trials, though participants were instructed that their choices would still count towards their final payout. Instead, after 3 seconds, a screen appeared which required participants to rate (on a scale of 1 to 3) their confidence in their decision: participants were carefully instructed to enter a "1" response if they were guessing entirely, a "2" response if they were "had some idea but were not sure" about their choice, and to reserve a "3" response until they were "more than 90% certain" that their choice was the correct one. Participants were told that though their confidence responses would not count towards their final payout, they should still answer as accurately as possible.

Schedule of trial presentation.

Blocks of Self trials alternated with blocks of Other trials, with block order (i.e. whether Self or Other condition appeared as the first block) counterbalanced across subjects. Each block was comprised of a 16 trial miniblock made up of each of the 8 training trial types repeated twice, followed by a 8 trial miniblock of each of the test trial types. The order of training and test trials was pseudorandomized and varied across blocks. The start of each miniblock was preceded with the relevant instruction which was presented for 2 seconds (i.e. "You Training trials", "Him" or "Her" Test trials). At the end of each training and test block, participants received cumulative feedback indicating their performance during that block (2 seconds).

In total, there were 12 blocks for each of the Self and Other conditions – i.e. 192 training trials, and 92 test trials, in each condition. Phase one consisted of three sessions of approximately 20 minutes each, separated by a 1 minute break during which time participants remained inside the MRI scanner.

Phase 2: Categorization (scanned)

In this phase, participants were presented with individual face pictures from the Self and Other hierarchies (excluding the profile picture depicting themself in the Self condition, or the friend in the Other condition). Each picture was repeated 4 times, duration 2.3 seconds with 0.8 seconds fixation cross between stimuli. Participants had to make a categorization judgment i.e. to determine whether the person belonged to the company with the yellow or blue logo. Presentation order was pseudorandom. 64 trials were divided over 2 experimental sessions lasting approximately minutes each. Participants had a 1 minute break between sessions during which time they remained inside the scanner.

Implicit Association Test (IAT: not scanned).

Following the end of the scanning part of the experiment, participants completed a version of the IAT test tailored to address our question of interest (Greenwald et al., 1998; Greenwald & Farnham, 2000) to probe the effectiveness of our experimental manipulation: i.e. the extent to which subjects incorporated themselves and their friend into the hierarchies.

We describe the task in detail below. Briefly, the rationale behind this paradigm is as follows and is broadly analogous to the Stroop effect (e.g. (Cohen et al., 1990)): consider that participants have actually incorporated themselves into the Self hierarchy, and that this company has a yellow logo (note color counterbalanced across participants). When participants view face pictures, they should be faster to

categorize in the **congruent** condition: when the yellow logo is displayed on the *same* side as the word "self" and when the blue logo is displayed on the *same* side as the word "other". In contrast, RTs should be slower in the **incongruent** condition: where the yellow logo is displayed above the word "other". Note that the words used in the IAT test – "self" and "other" were not used in other parts of the experiment (e.g. "You" trials and "Him" or "Her" trials in the Learning phase).

IAT Stimuli: The stimuli used during the IAT test consisted of the face pictures used in the main experiment, and also word stimuli**.**
Word stimuli**:** A set of 8 English pronouns were used: 4 of these pronouns – me, mine, myself, my – are known to be associated with the self concept and 4 pronouns – theirs, they, them, themselves – are known to be associated with the concept of another person. These pronouns were selected based on a pilot study to equate behavioural performance across both the Self and the Other conditions.

Trial Blocks (Figure below) As in the typical IAT test, the task was divided into several distinct blocks – 5 in our case.

The first block (Figure S1A) introduced the so-called "target-concept discrimination"; in this block participants viewed two rectangles in the top of the screen – one on the left, and one on the right – representing the respective colours of the logo of their company and their friend's company. The left-right position of the Self-colour and Other-colour logos was counterbalanced between participants. Participants viewed pictures of individuals from the Self and Other companies (with the exception of the profile pictures), and had 3 seconds to respond by button press (either Q or P, corresponding to left or right response) whether the individual was a member of the blue or the yellow company. A fixation cross, presented for 1 second, preceded the presentation of the next trial. There were 64 trials in this block: each picture presented 4 times. Order of stimulus presentation was pseudo-randomised across participants, in all blocks.

The starting position of the Self-colour (e.g. yellow) and Other-colour (e.g. blue) logos were counterbalanced between participants. This, in combination with the counterbalanced allocation of color (yellow/blue) to Self/Other hierarcy, allowed us to counterbalance whether participants performed the congruent trials – i.e. the word "self" and "other" positioned on the *same* side of the Self company colour logo as experienced during the first hierarchy learning phase – or the incongruent trials first.

The second block introduced the "attribute discrimination" (Figure S1B). Participants were presented with the word "self" on the left and the word "other" on the right side of the screen. In this section, participants were presented with one of the 8 word stimuli – pronouns (see above) – and had 3 seconds to respond as to whether they were related to the concept of the self or another person. A fixation cross, presented for 1 second, preceded the presentation of the next trial. 16 trials in this block, each pronoun repeated twice.

In the third block (Figure S1C) both the target concepts (i.e. colored logos) and the attributes (i.e. "self" or "other") were presented on the screen. The words ("self" and "other") and logos (yellow/blue) were presented on the same sides as in the preceding blocks (i.e. blocks 1 and 2). Participants viewed alternating pronouns and face pictures, and were required to respond according to whether they related to self/other concept, or yellow/blue logos, respectively. 96 trials in this block: 48 pronouns (each pronoun repeated 3 times) and 48 pictures (each picture repeated 3 times).

In the fourth block (Figure S1D) participants performed the same task as in block 2, but the position of the words "self" and "other" were reversed. 16 trials in this block, each pronoun repeated twice.

Finally, in the fifth block (Figure S1D) participants performed the same task as in block 3 with the exception that the position of the words "self" and "other" were as in block 4, and therefore swapped in side compared to block 3. 96 trials in this block: 48 pronouns (each pronoun repeated 3 times) and 48 pictures (each picture repeated 3 times).

Post-Experimental Debriefing (after completion of IAT test). Participants were carefully debriefing following the end of the IAT test. Included in this assessment was a test assessing participants' declarative knowledge of the hierarchy: pictures of the two sets of people were presented to participants, and they were asked to rank them in terms of their order in the hierarchy, with their performance timed.

Debriefing scores: Participants were also asked to evaluate how “real” the social rank dimension seemed – and to rate how much they felt part of the Self hierarchy, and how much they felt their friend was part of the Other hierarchy (see Table S8 for full details: appended below).
Then participants responded to a question that assessed their feelings about the level of power of the first-ranked and last-ranked individuals in both hierarchies, the Power Explicit Scores: “Can you tell me – use your gut instinct – how powerful each of these individuals feels to you on a scale from 0-low to 10-high”. After that, they were also asked to evaluate the level of realism of the social rank, the Social Realism Scores: 1) “How realistic does it feel when you see this person – the two individuals at the top of the hierarchies presented – that he/she is the head of the company/top dog on scale from 0 to 10 (10=realistic, 0 = unrealistic) and 2) “How realistic does it feel when you see this person – the two individuals at the bottom of the hierarchies presented – that he/she is the loser/lowest person on a scale from 0 to 10 (10=realistic, 0 = unrealistic)”. Then, we obtained four explicit measures on how much participants identified themselves and their friend with the respective hierarchies, the Explicit Identification Scores: 1) "How much on a scale from 0-not at all to 10-a lot did you feel you were part of the company in the YOU trials", 2) “How much on a scale from 0-not at all to 10-a lot did you feel your friend was part of the company in the HIM/HER trials", 3) “How much on a scale from 0-not at all to 10-a lot did you feel YOU were part of the company in the HIM/HER trials”, and 4) “How much on a scale from 0-not at all to 10-a lot, did you feel your FRIEND was part of the company in the YOU trials". Next, we asked some general questions about their friend: “How much do you like them? (on a scale 1 to 10, 1 is a little, 10 is a lot), and “How similar would you say they are to you in terms of overall perspective on life, personality, hobbies ? (from 1 to 10)”. Finally, participants rated all the face stimuli according to three traits: dominance, trustworthiness, attractiveness – i.e. How “trait” is this person? (1=not at all, 9=extremely), use your gut feelings.

"In phase 2, when you saw a picture of an individual who was more highly ranked in the company, how "real" did it seem that they were more highly ranked or had more power in the company etc? Please rate this on a scale of 1-10 (10 = a lot, 1 = not at all)- as an example, if when you saw the most highly ranked guy you thought to yourself that's the topdog/head-guy, then your answer is likely to be nearer the 10 end of the scale”

“How much on a scale from 0-not at all to 10-a lot did you feel you were part of the hierarchy in the YOU trials?

“How much on a scale from 0-not at all to 10-a lot did you feel your friend was part of the company in the hierarchy in the HIM/HER trials?”

Behavioral analyses. Analyses were conducted using SPSS software (www.spss.com), Matlab 7.0 (www.mathworks.com/products/matlab).
Implicit Association Test. Before analysing the data from the IAT, we applied the reduction-data procedure describe by (Greenwald et al., 2003). Specifically we followed these steps: 1) subjects for whom more than 10% of trials had a latency less than 300ms were eliminated; 2) subjects for whom more than 20% of trials were error trials were eliminated; 3) the means of only the correct latencies for block 3 and block 5 were computed; 4) the pooled standard deviation for all trials in block 3 and block 5 was computed; 5) Each error latency in block 3 and 5 was replaced by the block mean computed at point 3 plus 600ms; 6) The difference between the mean latencies of block 3 and block 5 was computed; 7) The difference computed in step 6 was divided by the pooled standard deviation calculated in step 4 to obtain the correct IAT measure – the D measure – used in all the analyses. Following this procedure, 6 participants were excluded from the analysis.

Computational Models.

Sequential Monte-Carlo (SMC) model. SMC models (Doucet et al., 2000)are a type of state-space inference model that aims to infer the underlying state of an evolving dynamic system: in this case the level of power of a set of individuals. These hidden states (i.e. power levels) diffuse across training trials according to a Gaussian random-walk model (a non-zero variance for which models forgetting). Dominance decisions are assumed to be generated according to an observation process – here a random choice based on a sigmoid of the discrepancy in power between the individuals. The SMC method, which is a form of Bayesian filter, captures the way that a participant can use information about the hierarchy that is acquired over the course of the experiment to make inferences about these powers. We consider an on-line filter, according to which estimates of the powers are based on past data up to the current trial.

SMC models – also known as particle filters – relax the conventional assumption for linear, Gaussian, Kalman filters, that the probability density function (pdf) of the inferred variables (i.e. power) is a normal distribution, thereby extending their flexibility in modelling complex multi-modal distributions in a range of domains (e.g (Doucet et al., 2000)). In the SMC model, each particle (here, *N*=10,000) contains one set of values for the hidden state variable (i.e. power). Hence a particle can be viewed as representing a hypothesis about the rank ordering of items within the hierarchy: the population of particles, therefore, constitutes a multimodal (i.e. 9-dimensional, given 9 items in each hierarchy) pdf of rank. Particles are initialized with equal weight (see below), with their weights being updated on each training trial depending on the likelihood of the trial outcome given the hypothesis concerning rank ordering they represent. A particle resampling step ensures that the density of particles is highest in regions of the (9-dimensional) space that are likely given the history of observed data (i.e. have highest weights), by tending to replace conditionally unlikely particles (i.e. with low weights) with new, more appropriate, ones.

Prior model: the state variable (i.e. $\vec{x}_0$, denoting power) is initialized a normal distribution with fixed initial variance σ_0^2 (=10), and zero mean (Eq 1). The state process is described as a Gaussian random walk with evolution variance σ^2 (free parameter)(Eqn 2) – this instantiates a form of imperfect memory (i.e. forgetting), in order to account for participants needing ~ 200 trials to achieve proficiency on the task. The observation model (Eqn 3) is a sigmoid function of the difference between the distributions of the two items presented in a given training trial *t*: parameterized by beta i.e. the item with current highest expected value (a_t) and that with the lower value (b_t). y_t = 1 denotes the situation when the highest valued item is the correct response.

$$\vec{x}_0 \sim \mathcal{N}\left(\vec{0}, \sigma_0^2 \mathbf{I}\right) \quad (1)$$

$$\vec{x}_t | \vec{x}_{t-1} \sim \mathcal{N}\left(\vec{x}_{t-1}, \sigma^2 \mathbf{I}\right) \quad (2)$$

$$g(y_t = 1 | a_t, b_t, x_t) = \frac{1}{\left(1 + e^{-\beta * (x_{a_t t} - x_{b_t t})}\right)} \quad (3)$$

Particle filter: Let i index particles, of which there are N $(= 10{,}000)$. Particles are initialized as samples from a normal distribution, with zero mean and variance σ_0^2 (Eqn 4), each with an equal weight ($\widetilde{w}^{(i)}$), which are then normalized (Eqn 5). State process equation for the particles – note since no feedback was provided during test trials, we assumed that this process only occurred during training trials (Eqn 6). The unnormalized weights ($w_{t+1}^{(i)}$) of the particles are updated using the observation model and the normalized weights from the previous timestep (Eqn 7).

$$\vec{x}_0^{(i)} \sim \mathcal{N}(0, \sigma_0^2 \mathbf{I}) \quad (4)$$

$$\widetilde{w}^{(i)}{}_0 = \frac{1}{N} \quad (5)$$

$$\vec{x}_t^{(i)} \sim \mathcal{N}\left(\vec{x}_{t-1}^{(i)}, \sigma^2 \mathbf{I}\right) \quad (6)$$

$$w_{t+1}^{(i)} = g\left(y_t \middle| a_t, b_t, \vec{x}_t^{(i)}\right)\widetilde{w}_t^{(i)} \quad (7)$$

N_{eff} determines the threshold for resampling (Eqn 8).

$$N_{eff} = \frac{1}{\sum_{i=1}^{N} \widetilde{w}_t^{(i)} \cdot \widetilde{w}_t^{(i)}} \quad < 0.25N \quad (8)$$

If $N_{eff} \quad < 0.25N$

then resample (with replacement):

$$a_t^{(i)} \sim \text{Categorical}\left(\left\{\frac{w_t^{(i)}}{\sum_{i=1}^{N} w_t^{(i)}}\right\}_{i=1}^{N}\right) \quad (9)$$

$$x_t^{(i)} = x_t^{(a_t^{(i)})} \quad (10)$$

$$\widetilde{w}_t^{(i)} = \frac{1}{N} \quad (11)$$

$$Otherwise\ \widetilde{w}_t^{(i)} = w_t^{(i)} \quad (12)$$

The estimate of marginal likelihood is:

$$\hat{p}(y_{1:T}) \quad = \prod_{t=1}^{T} \frac{1}{N} \sum_{i=1}^{N} w_t^{(i)} \quad (13)$$

Note: an internal variable of the model used to interrogate the fMRI data is the *SMC model choice entropy*: $H(y_t) = -p \log p - (1-p)\log(1-p)$; where $p = P(y_t = 1)$ the probability of selecting the correct option, given the weighted set of particles at the current trial.

RL model (RL-ELO).

In the RL-ELO model, rather than updating based on the difference between trial outcome and current value (as in Rescorla Wagner), the value update is a function of the difference in current values between the two items (i.e. indexed by their positions, left and right, which was randomized on every trial): $V_{L,t}$ & $V_{R,t}$ We term this model RL-ELO because of its relationship to algorithms used to update rankings (termed "ELO") in multiagent scenarios (e.g. chess) – though it should be noted that it can be shown to be a version of a policy gradient algorithm (Williams, 1992).

Initialize all ranks at V_0=0
free parameters: α = learning rate; β=temperature
Training trial at time *t* with items on left and right sides of screen
Probability of choosing left item, and right item:

$$p_{L,t} = \frac{1}{\left(1+e^{-\beta*(V_{L,t}\ -\ V_{R,t}\ )}\right)} \quad (1)$$

$$p_{R,t} = 1 - \ p_{L,t} \quad (2)$$

if L item is correct choice:

$$p_{win,t} = p_{L,t} \quad (3)$$

$$I_{L,t} = 1, I_{R,t} = -1 \quad (4)$$

if R item is correct choice:

$$p_{win,t} = p_{R,t} \quad (5)$$

$$I_{L,t} = -1, I_{R,t} = 1 \quad (6)$$

Update values of both items:

$$V_{L,t+1} = \alpha * I_{L,t} * (1 - p_{win,t}) + V_{L,t} \quad (7)$$

$$V_{R,t+1} = \alpha * I_{R,t} * (1 - p_{win,t}) + V_{R,t} \quad (8)$$

Note that we also examined a variant of the model, termed RL-ELO$_F$, which incorporated an extra free parameter (σ) that controlled the amount of Gaussian noise (i.e. mean = 0 , SD = σ) that was added to the values at each timestep. This parameter instantiated a form of forgetting, akin to the role of the evolution variance parameter in the SMC model.

Value Transfer model (von Fersen et al., 1991)
This model incorporates the standard update term from Rescorla Wagner, but also includes an indirect component: the incorrect item in a training trial has its value updated with a proportion (i.e. theta %) of the correct item.
Trial outcomes are +1 for correct choice, and -1 for incorrect choice.
3 free parameters: α = learning rate; β=temperature ; θ = transfer factor
Training trial at time *t* with items on left and right sides of screen
Probability of choosing left item, and right item:

$$p_{L,t} = \frac{1}{\left(1+e^{-\beta*(V_{L,t} - V_{R,t})}\right)} \quad (1)$$

$$p_{R,t} = 1 - p_{L,t} \quad (2)$$

if L item is correct choice, trial outcome & indicator variable (for indirect update):

$$O_{L,t} = 1, O_{R,t} = -1 \quad (3)$$

$$I_{L,t} = 0, I_{R,t} = 1 \quad (4)$$

If R item is correct choice, trial outcome & indicator variable:

$$O_{L,t} = -1, O_{R,t} = 1 \quad (5)$$

$$I_{L,t} = 1, I_{R,t} = 0 \quad (6)$$

Calculate direct component of update:

$$\delta V\_direct_{L,t} = (O_{L,t} - V_{L,t}) * \alpha \quad (7)$$

$$\delta V\_direct_{R,t} = (O_{R,t} - V_{R,t}) * \alpha \quad (8)$$

Calculate indirect component of update:

$$\delta V\_indirect_{L,t} = V_{R,t} * I_{L,t} * \theta \quad (9)$$

$$\delta V\_indirect_{R,t} = V_{L,t} * I_{R,t} * \theta \quad (10)$$

Total update:

$$V_{L,t+1} = \delta V\_direct_{L,t} + \delta V\_indirect_{L,t} \quad (11)$$

$$V_{R,t+1} = \delta V\_direct_{R,t} + \delta V\_indirect_{R,t} \quad (12)$$

Rescorla Wagner. As for Value transfer model, where theta parameter is set to zero.

Computational model fitting. We quantified the fit of all models and a base model (random choice) – to participant's choice behavior during training and test trials. We used a maximum likelihood estimation procedure and optimized a separate set of parameters for each participant (Wimmer et al., 2012). We report the negative log likelihood of each model, and the corresponding BIC measure which penalizes more complex models.

FMRI Design & Analysis.

fMRI design. The temporal pattern of stimulus presentation was designed to maximise statistical efficiency whilst preserving psychological validity, in line with established procedure (Frackowiak et al., 2004; Friston et al., 1998; Josephs and Henson, 1999). Importantly, the haemodynamic response to events that occur a few seconds apart is explicitly modelled (via a haemodynamic response function), and therefore can be estimated separately for each event type by implementing the general linear model as is standard when using statistical parametric mapping software (SPM8) (www.fil.ion.ucl.ac.uk/SPM) (also see below) (Friston et al., 1998).

Functional imaging acquisition parameters. T2 weighted gradient-echo planar images (EPI) with BOLD (blood oxygen level dependent) contrast were acquired on a 3.0 tesla Siemens Allegra MRI scanner using a specialized sequence to acquire whole brain coverage, whilst minimizing signal dropout in the medial temporal lobe and ventromedial prefrontal cortex(Weiskopf et al., 2006). We used the following scanning parameters to achieve whole brain coverage: 48 oblique axial slices angled at 30^0 in the anterior-posterior axis, TR 2.88 seconds, TE 30ms, 2mm thickness (1mm gap), in-plane resolution 3x3 mm, z-shim -0.4mT/m*ms, negative phase encoding direction. High-resolution (1x1x1mm) T1-weighted structural MRI scan were also acquired for each participant after functional scanning. These were coregistered to the functional EPIs, and averaged across participants to aid localization.

fMRI data preprocessing. Images were analyzed in a standard manner using the statistical parametric mapping software SPM8 (www.fil.ion.ucl.ac.uk/SPM). After the first six "dummy volumes" were discarded to permit T1 relaxation, EPI images were spatially realigned and unwarped using fieldmaps,

followed by spatial normalization to a standard EPI template. Normalized images were smoothed using a gaussian kernel with full width at half maximum of 8mm.

Phase 1 (Learn) fMRI data analysis. Following preprocessing, the event-related fMRI data were analyzed in SPM8 using the general linear model (GLM) following established procedures (Frackowiak et al., 2004; Friston et al., 1998). We targeted our analyses to detect brain regions whose activation pattern during test and training trials significantly correlated with participant-specific trial-by-trial parametric regressors: obtained from key hidden variables of the relevant computational models (SMC, RL-ELO). Note that following previous work (Daw et al., 2006; Wimmer et al., 2012) suggesting that using individually optimized parameters to analyse the fMRI data tends to lead to noisy fitting, we used a single set of parameters for the SMC, and the RL-ELO model (i.e. best fit parameters across the group).

Specification of first-level design matrix.
Test trials. As a first step, the 5 second period during which item pair and confidence rating were displayed during test trials was modeled as a boxcar function and convolved with the canonical haemodynamic response function (HRF) to create regressors of interest. All test trial types (i.e. 8 pairs: see above) were modeled within these regressors, with one regressor for the Self condition and one for the Other condition. These participant-specific parametric regressors were also convolved with the HRF, leading to the height of the HRF for a given event being modulated accordingly. Thus, these parametric regressors model BOLD signal changes that covary with a specific internal variable of the SMC model on a given trial (i.e. over and above non-specific effects captured by earlier parametric regressors such as RT). Further, participant-specific movement parameters were included as regressors of no interest. A high pass filter with a cutoff of 180 seconds was employed. Temporal autocorrelation was modelled using an AR(1) process.

In the first model, the parametric modulator was the difference between SMC model estimates of item power: this was a trial-by-trial variable obtained from the SMC model, by taking the expectation over the difference between estimated power of the items presented.

Training trials. As a first step, the 5 second period during which item pair and outcome was displayed during training trials was modeled as a boxcar function and convolved with the canonical haemodynamic response function (HRF) to create regressors of interest. All training trial types (i.e. 8 pairs: P1 vs P2, P2 vs P3....) were modeled within these regressors, with one regressor for the Self condition and one for the Other condition.

In this model, the parametric regressor included was termed the **hierarchy update index**: trial-by-trial estimates derived from the SMC model, to capture the change in hierarchy knowledge consequent on feedback on a given training trial.

For each pair of items (e.g. 1 & 2) we computed a trial-by-trial KL divergence measure, with respect to the probability of one item winning against the other (i.e. before and after feedback).

$$D_{KL}(p \mid q) = \sum_{i=1}^{2} p(i) \log \frac{p(i)}{q(i)}$$

where p(1) is the probability of item_1 winning against item_2 following updating after feedback, and q(1) is that before feedback; where the probability of winning was calculated as elsewhere using a sigmoid function parameterized by beta. Note p(2) = 1 – p(1), and similarly q(2) = 1 – q(1). Then the KL divergence was summed across all 36 pairs. This analysis, therefore, for set up to identify regions where neural activity correlated positively with a change in participants' hierarchy knowledge. Note that since no feedback was provided, there was no change in hierarchy knowledge during test trials.We also set up a model to identify neural regions that showed a correlation with the chosen power – computed from the SMC model as the expectation over distribution of the item power – during training trials. SMC estimated chosen power was the parametric modulator entered into the design matrix.

Neural model comparison: Following previous work (Ashby and Waldschmidt, 2008; Niv et al., 2015; Wilson and Niv, 2015), we ran separate GLMs for the SMC and RL-ELO models using using parametric regressors relating to the hierarchy update index (i.e. using a single set of parameters for each model best fit to the group behavioral data). The negative log likelihood (NLL) of each model, calculated separately for each region of interest (e.g. amygdala) was:

$$NLL = -n * (ln\,(\sqrt{2\pi\sigma^2} + 0.5)$$

where σ^2 is the variance of the residuals from the GLM, and n is the number of scans. We then calculated the relative difference in BIC between models: for completeness, and also following previous work (Niv et al., 2015) we tested whether the difference between model NLL was significantly different from zero using permutation testing.

Phase 2 (Categorization). fMRI data analysis. Following preprocessing, the event-related fMRI data were analyzed in SPM8 using the general linear model (GLM) following established procedures (Frackowiak et al., 2004; Friston et al., 1998). We set up a parametric model to detect brain regions whose activation pattern exhibited a significant linear correlation with the rank of person in the Self and Other hierarchies.

Specification of first-level design matrix. The 2 second trial period during which the face image was displayed on the screen and participants made their response, was modeled as a boxcar function and convolved with the canonical haemodynamic response function (HRF) to create regressors of interest.

Parametric model. Separate regressors were included for Self and Other conditions. **Rank**, from 1 to 9, was included as a parametric modulator in the GLM: linear and quadratic components were modeled. Note that rank 5 was not included in the model since participants did not view the profile pictures of themselves or their friends during this phase. Thus, these regressors model BOLD signal changes that covary with specific indices on a given trial (e.g. the rank of a person).

"Separate rank" model: This model was used for two purposes: i) firstly, it was used as an illustrative model to graphically represent the linear relationship between neural activity in a given brain region (e.g. amygdala) and person rank (see Figure 6; also see(Winston et al., 2002) & Kumaran et al 2012 for a similar useage). In this case, the parametric model specified above was used for statistical inference- i.e. to ask which brain regions show a significant linear correlation between the amplitude of neural activity and person rank. ii) this model was also used to ask whether activity in the MPFC ROI, defined based on orthogonal selection contrast (i.e. from during a separate scanning phase) distinguished rank extremes

Model Estimation. Model estimation proceeded in two stages. In the first stage, condition-specific experimental effects (parameter estimates, or regression coefficients, pertaining to the height of the canonical HRF) were obtained via the GLM in a voxel-wise manner for each participant. In the second (random-effects) stage, participant-specific linear contrasts of these parameter estimates, collapsed across the 2 sessions, were entered into a series of one-sample t tests [as is standard when using SPM(Frackowiak et al., 2004)], each constituting a group-level statistical parametric map.

Statistical inference.

Voxel-based analyses. ***Voxel-based analyses***. We report results in a priori regions of interest - the hippocampus, amygdala, vmPFC and MPFC - where activations are significant at $p<0.001$ uncorrected for multiple comparisons, and survive small volume correction (SVC) for multiple comparisons (at $p<0.05$ corrected) using SPM8. For the SVC procedure we used anatomical masks, for the bilateral hippocampus and amygdala. For the vmPFC, and MPFC we used 6mm spheres centred on coordinates (MNI x, y, z -4,

52, -14 and 6, 48, 4) derived from previous related studies: (Kumaran et al., 2012; Kumaran and Maguire, 2005).

Activations in other brain regions were only considered significant if they were significant at a level of $p<0.001$ uncorrected, and additionally survived whole brain FWE correction at the at the peak level or cluster level ($p<0.05$ corrected, with cluster threshold defined at $p<0.001$), in line with established procedures(Frackowiak et al., 2004).Reported voxels conform to MNI (Montreal Neurological Institute) coordinate space. Right side of the brain is displayed on the right side.

Region of Interest (ROI) analyses. We performed anatomically defined ROI analysis (using the MarsBar SPM toolbox: http://marsbar.sourceforge.net/) in the amygdala, hippocampus, and vMPFC (defined in the same way as for the SVC procedure outlined above). Further, we defined a functional MPFC ROI (Figure 7) based on the results of an analysis during the Learn phase (i.e. significantly greater correlation with chosen power during Self (cf Other) condition) that was subsequently used in a different scanning phase (i.e. the Categorization phase). ROI defined at a level of $p<0.001$ uncorrected. We also defined a ROI proximate to the fusiform face area based on a constrast defined on the categorization data (i.e. specifying the onset of face events vs implicit baseline, at $p<0.001$ uncorrected).

It is important to note that these analyses treat data from a ROI as if it was from a single voxel and hence no correction for multiple comparisons is necessary. Results, therefore, were considered statistically significant where they pass a threshold of $p<0.05$.

Selection contrast is unbiased with respect to contrasts of interest. ROI analyses are widely held to be a powerful tool for affording additional insights, above and beyond that provided by univariate fMRI analysis(Kriegeskorte et al., 2009). Recent work has highlighted potential shortcomings of previous work, and established a theoretically principled approach for carrying out an ROI analysis. Importantly, our analyses fulfil the criteria outlined by (Kriegeskorte et al., 2009): the definition of these ROI is *unbiased* – either based on a different portion of the data (i.e. training trials vs test trials), or on a different scanning phase (i.e. Learn phase vs Categorization phase) – and therefore statistically independent from the effects we examine.

Psychophysiological Interaction (PPI) Analysis
A PPI analysis is employed to identify the presence of functional coupling between different brain regions, by showing that activity in a distant region can be accounted for by an interaction between the influence of a source region and an experimental parameter (Friston et al., 1997). We followed established procedures (O'Reilly et al., 2012) to perform the PPI analysis by creating a GLM that included regressors capturing i) the physiological effect (here, the time series of activity in the MPFC seed region) ii) the psychological contrast of interest: here, hierarchy update: Self > Other (i.e. designed to identify regions showing a greater correlation with hierarchy update index in Self, as compared to Other, condition (see Main Text). iii) psychophysiological interaction term (i.e. physiological effect x psychological contrast of interest)

Specifically: we used SPM8 to first extract the time series (i.e. physiological effect) for the peak voxel in the MPFC (i.e., 6 mm sphere centered on peak coordinate in the group analysis x, y, z, = -6, 46, 12), identified in the correlation of training trial related activity in the Learn phase with the hierarchy update index, collapsed across both Self and Other conditions (see Figure 4B and Table S3A). Next, we calculated the psychological contrast of interest (i.e. hierarchy update: Self > Other). Finally, we calculated the product of the first two signals. The physiological effect and psychophysiological interaction were entered as regressors within the GLM: in addition, we entered a regressor capturing the psychological contrast of interest (i.e. hierarchy update: Self > Other) *without* the interaction with physiological effect. The effect of the psychophysiological interaction term was assessed for each participant and entered into a second level group-level analysis.
The configuration of the PPI GLM, therefore, allows us to ask in which brain regions the magnitude of functional coupling of neural activity with the MPFC seed region shows a significantly greater correlation with the amount by which hierarchy knowledge changes in the Self, as compared to the Other condition – *above and beyond that* explained by differences in the correlation between the hierarchy update index in the Self and Other conditions.

Supplemental References

Ashby, F.G., Waldschmidt, J.G., 2008. Fitting computational models to fMRI. Behav. Res. Methods 40, 713–21.

Cohen, J.D., Dunbar, K., McClelland, J.L., 1990. On the control of automatic processes: a parallel distributed processing account of the Stroop effect. Psychol. Rev. 97, 332–61.

Daw, N.D., O'Doherty, J.P., Dayan, P., Dolan, R.J., Seymour, B., 2006. Cortical substrates for exploratory decisions in humans. Nature 441, 876–9.

Doucet, A., Godsill, S., Andrieu, C., 2000. On sequential Monte Carlo sampling methods for Bayesian filtering. Stat. Comput. 197–208.

Frackowiak, R. S. 2004. Human brain function. K. J. Friston, C. D. Frith, R. J. Dolan, C. J. Price, S. Zeki, J. T. Ashburner, & W. D. Penny (Eds.). Academic press.

Friston, K.J., Buechel, C., Fink, G.R., Morris, J., Rolls, E., Dolan, R.J., 1997. Psychophysiological and modulatory interactions in neuroimaging. Neuroimage 6, 218–229.

Friston, K. J., Fletcher, P., Josephs, O., Holmes, A. N. D. R. E. W., Rugg, M. D., & Turner, R. ,1998. Event-related fMRI: characterizing differential responses. Neuroimage, 7(1), 30-40.

Greenwald, A.G., Farnham, S.D., 2000. Using the Implicit Association Test to measure self-esteem and self-concept. J. Pers. Soc. Psychol. 79, 1022–1038.

Greenwald, A.G., McGhee, D.E., Schwartz, J.L., 1998. Measuring individual differences in implicit cognition: the implicit association test. J. Pers. Soc. Psychol. 74, 1464–80.

Greenwald, A.G., Nosek, B. a., Banaji, M.R., 2003. Understanding and using the Implicit Association Test: I. An improved scoring algorithm. J. Pers. Soc. Psychol. 85, 197–216.

Johnson, J.D., Muftuler, L.T., Rugg, M.D., 2008. Multiple repetitions reveal functionally and anatomically distinct patterns of hippocampal activity during continuous recognition memory. Hippocampus 18, 975–980.

Josephs, O., & Henson, R. N. ,1999. Event-related functional magnetic resonance imaging: modelling, inference and optimization. Philosophical Transactions of the Royal Society B: Biological Sciences, 354(1387), 1215-1228.

Kriegeskorte, N., Simmons, W.K., Bellgowan, P.S., Baker, C.I., 2009. Circular analysis in systems neuroscience: the dangers of double dipping. Nat Neurosci 12, 535–540.

Mitchell, J.P., Macrae, C.N., Banaji, M.R., 2006. Dissociable Medial Prefrontal Contributions to Judgments of Similar and Dissimilar Others. Neuron 50, 655–663.

Niv, Y., Daniel, R., Geana, A., Gershman, S.J., Leong, Y.C., Radulescu, A., Wilson, R.C., 2015. Reinforcement learning in multidimensional environments relies on attention mechanisms. J. Neurosci. 35, 8145–8157.

O'Reilly, J.X., Woolrich, M.W., Behrens, T.E.J., Smith, S.M., Johansen-Berg, H., 2012. Tools of the trade: Psychophysiological interactions and functional connectivity. Soc. Cogn. Affect. Neurosci. 7, 604–609.

von Fersen, L., Wynne, C.D., Delius, J.D., Staddon, J.E., 1991. Transitive inference formation in pigeons. J. Exp. Psychol. Anim. Behav. Process. 17, 334–341.

Weiskopf, N., Hutton, C., Josephs, O., & Deichmann, R., 2006. Optimal EPI parameters for reduction of susceptibility-induced BOLD sensitivity losses: a whole-brain analysis at 3 T and 1.5 T. Neuroimage, 33(2), 493-504.

Williams, R.J., 1992. Simple statistical gradient-following algorithms for connectionist reinforcement learning. Mach. Learn. 8, 229–256.

Wilson, R.C., Niv, Y., 2015. Is Model Fitting Necessary for Model-Based fMRI? PLoS Comput. Biol. 11.

Wimmer, G.E., Daw, N.D., Shohamy, D., 2012. Generalization of value in reinforcement learning by humans. Eur. J. Neurosci. 35, 1092–1104.

Winston, J.S., Strange, B. a, O'Doherty, J., Dolan, R.J., 2002. Automatic and intentional brain responses during evaluation of trustworthiness of faces. Nat. Neurosci. 5, 277–283.
